# Welding Techniques for Magnesium Alloy Joints: A Comprehensive Review

**DOI:** 10.3390/ma19153355

**Published:** 2026-08-06

**Authors:** Milos Poliak, Piotr Czyzewski, Przemyslaw Kubiak, Damian Frej, Adam Rylski, Marek Wozniak, Krzysztof Siczek

**Affiliations:** 1 Department of Road and Urban Transport, University of Žilina, 010-26 Žilina, Slovakia; milos.poliak@fpedas.uniza.sk; 2 Division of Ecotechnics, Lodz University of Technology, 90-924 Łódź, Poland; piotr.czyzewski@poczta.onet.pl (P.C.); przemyslaw.kubiak@p.lodz.pl (P.K.); 3 Faculty of Mechatronics and Mechanical Engineering, Kielce University of Technology, 25-314 Kielce, Poland; dfrej@tu.kielce.pl; 4 Institute of Materials Science and Engineering, Lodz University of Technology, 90-924 Łódź, Poland; adam.rylski@p.lodz.pl; 5 Department of Vehicles and fundamentals of machine Design. Lodz University of Technology, 90-924 Łódź, Poland; marek.wozniak.1@p.lodz.pl

**Keywords:** Mg alloys, welding methods, filler material, Mg high-entropy alloys, metallic interlayers, preheating, friction stir welding, laser beam welding

## Abstract

**Highlights:**

- MIG, TIG, LBW, and FSW are most applied to Mg alloys, where TIG is favored for quality, and FSW is the top choice for solid-state welding.
- Common filler metals include AZ61A, AZ101A, AZ92A, and EZ33A, available as TIG rods or MIG wire spools.
- Laser–TIG and laser–MIG can give strong joints (95% to 100% of the BM’s tensile strength reaching up to 550 MPa), reduce gaps and defects, but are expensive; GMAW–GTAW improves deposition and stability.
- LBW, EBW, and FSW of Mg-inclusive HEAs provide low density and significant strength but face challenges with cracking and porosity of elements.

**Abstract:**

Magnesium (Mg) alloys are crucial for lightweight automotive design, underscoring the need for effective welding despite their poor weldability and susceptibility to defects such as cracks. The paper was prepared by reviewing available scientific databases of studies and patents using appropriate keywords. It reviews the properties, applications, and welding methods (fusion, friction, diffusion, explosive, and hybrid) of Mg alloys, assessing their advantages, disadvantages, and precautions. The importance of understanding the mechanical behavior and structural integrity of welded joints was highlighted. The impact of process variables on weld microstructure and properties, along with future research areas, is also addressed. AZ-series Mg alloys were found to be favored for weldability. For them, primary welding methods include GTAW, GMAW, LBW, and FSW. Trends emphasize solid-state techniques, advanced dissimilar metal joining, and AI optimization to enhance welding efficiency and quality. Welding of modern Mg-inclusive high-entropy alloys is under intensive development.

## 1. Introduction

Magnesium and its alloys offer a beneficial high strength-to-weight ratio [1], excellent electromagnetic interference (EMI) shielding capability, enhanced thermal conductivity, impressive damping properties [2], and are easy to recycle [3], which is essential to the circular economic model. These alloys are usable in the automotive, aerospace, and medical fields. The use of Mg alloys as biomaterials arises not only from their remarkable biocompatibility but also from their inherent characteristics, particularly the dissolution rate in bodily fluids [4]. The yield strengths (YSs) and density of these alloys are comparable to those of human bone [5,6]. The similarity in elastic moduli and densities of these alloys to human bone addresses the implant-bone mismatch [7]. Interestingly, Mg is a vital element for human metabolism and bone health, with shortages leading to osteoporosis [8], which can influence the implant design. Mg-based alloys are beneficial as implants due to their ability to dissolve in the bloodstream during healing [9,10]. The production of implants often requires joining components using, among other things, appropriately selected welding techniques [10,11].

Mg alloys are considered environmentally friendly [12], but current Mg alloys derived from primary sources used in automobiles do not meet true sustainability criteria, given their environmental impact [13]. Reducing life cycle environmental effects could enable these alloys to be labeled as green [13]. While Mg powder poses fire hazards [14], its use in alloys leads to reduced pollution and promotes sustainability [15,16,17]. Recycling of wastes from various welding processes of Mg alloys is an important problem in various production branches using these alloys [18]. Mg is becoming increasingly important in industrial applications [19], being significantly lighter than Al and steel [20], and is suited for biomaterials [21,22], sustainable engineering [13,23,24], and lightweight structures [21,25].

Alloys based on Mg exhibit certain disadvantages, notably a strong tendency for oxidation, low melting temperature (650 °C) during welding [26], and inadequate formability at ambient temperatures [27]. Weak formability at room temperature is linked to a dominant basal crystallographic texture due to significant basal slips during thermomechanical processing, or more precisely, by the severe shortage of alternative deformation mechanisms caused by the dominant texture [28,29,30,31]. The hexagonal close-packed (HCP) structure offers limited deformation modes and pathways, resulting in low formability at room temperature for Mg alloys, which suffer from high plastic anisotropy [27,32,33,34]. The low boiling point (1100 °C) and low melting point (650 °C) result from the weak intermolecular forces between the atoms, the hexagonal close-packed (HCP) arrangement, and the related crystallographic anisotropy [27]. The low melting point limits the application of these materials at higher temperatures. These alloys are temperature-sensitive as their strength and elastic modulus reduce with rising temperatures [35,36,37]. The percentage elongation rises with higher temperatures until reaching the melting point, then sharply declines to nearly zero. This has hindered the effective use of many Mg-based alloys in the industry.

Issues related to the welding of Mg-based alloys are examined. These consist of widening and swelling of the weld pool (especially for thick materials) [38], inclusions and contamination of the welding area with porous oxides, undercutting, a decrease in mechanical properties (strength–ductility balance), and cracking within the welded joints [38]. Spattering and non-continuous weld beads are likewise seen in Mg-based materials [39,40]. These difficulties impact the structural stability of the Mg-based alloys, leading to diminished mechanical properties.

The weldability and structural strength of Mg-based alloys can be enhanced through two methods. Initially, improving various welding methods and their impact on the mechanical characteristics of Mg-based alloys is crucial. The primary welding techniques are fusion welding and solid-state welding. The technique influences the adhesion at the fusion zone (FZ), heat-affected zones, and their effect on the microstructural and mechanical properties of the alloy.

Common fusion welding techniques include tungsten inert gas welding (TIG) [41,42,43,44], laser or electron beam welding (LBW or EBW) [42,44,45], and hybrid laser–arc welding [46,47]. Friction stir welding (FSW) [48] and resistance spot welding (RSW) [49] are the primary solid-state welding methods for Mg alloys [50]. The alternative method involves optimizing the welding process parameters to decrease defects and enhance weld quality [51]. The quality of the weldment is directly connected to structural integrity, which is influenced by the process parameters [50,52,53]. These factors encompass arc travel speed, welding current, electrode feed rate, wire electrode length, welding voltage, electrode angle, quality of shielding gases, electrode size, and polarity [54,55].

Limited studies have concentrated on various welding techniques for Mg-based alloys, their selection, and the impact on the mechanical properties and structural integrity of Mg alloys [52,56,57,58,59]. Currently, there are still only a limited number of thematic reviews and critical assessments regarding process variable parameters related to the mechanical and microstructural characterization of Mg alloys [52,59]. A summary of processing parameters and their impact on reducing weld defects, as well as their influence on mechanical properties and structural integrity, is provided in [50]. The authors examined welding techniques and process parameters and their influence on the mechanical properties and structural stability of Mg and its alloys. The processes of fusion and solid-state welding for these alloys were examined with a focus on mechanical characterization. Laser welding proved to be the most efficient fusion welding method for many Mg alloys, while the leading solid-state process was friction stir welding. The significance of process variables like heat inputs, welding speed, and post-weld treatments on the microstructural changes, as well as on the mechanical and physical properties of the different zones of the weld joints, was outlined. The weldment was the most likely to fail because of phase changes, imperfections like microporosity, and comparatively coarse grain sizes following solidification. The present article reviews how processes and variables impact microstructure and mechanical properties in welding Mg and other alloys. The discussion on the physical metallurgy of Mg and its alloys is followed by considerations on welding types of Mg and its alloys, including fusion welding methods like GMAW, GTAW, CMT, plasma-based techniques, RSW, LBW, EBW, and the use of filler materials. The next section of the review relates to solid-state welding, including USW, diffusion techniques, EXW, FSW, and FSSW. The following section discusses hybrid welding of Mg alloys, including laser–GMAW, laser–GTAW, GMAW–GTAW, laser–USW, and plasma–GMAW techniques. The next section of the review treated brazing of Mg alloys. The last section related to the welding of HEAs of Mg alloys, followed by conclusions and advice.

## 2. Physical Metallurgy of Mg and Its Alloys

Mg is the lightest structural metal with a density of 1.74 g/cm^3^ and a hexagonal close-packed (HCP) crystal structure. That single structure governs almost all characteristics of its physical metallurgy: limited room-temperature slip systems, pronounced deformation twinning, strong crystallographic texture, and the need for careful alloy design to unlock strength and formability simultaneously. Pure Mg has a c/a ratio of ≈1.624, leading to limited slip systems for formability. Basal slip is dominant at low strains, while prismatic and pyramidal slips activate at higher temperatures or strains, with texture reorientation influencing activation in twin lamellae [60].

Twinning, which is the most distinctive deformation mechanism in Mg, promotes strain hardening and yield asymmetry. The {$10\bar{1}2$}⟨$10\bar{1}1$⟩ twin reorients the c-axis by ~86°, nucleating and thickening with strain [60]. Contraction twins facilitate double twins and enhance crack nucleation, increasing fracture risk during tension [61]. Twin formation creates two separate domains in the grain, one that deforms elastically and the other that deforms plastically [62]. In situations where twinning is dominant, the yield stress remains constant from slow to very fast speeds (2800 s^−1^) because twinning needs little heat to happen [63]. At room temperature, many double twins turn into shear bands, while at very low temperatures, unique twin formations occur. The density of geometrically necessary dislocations is high near double twins and low near single tension twins [64]. Basal texture in wrought products arises from rolling and extrusion, leading to in-plane asymmetry. Rare-earth (RE) additions create a “RE texture” that enhances isotropy [65]. Pre-straining induces {$10\bar{1}2$} twins, improving formability [66]. Bimodal textures from equal-channel angular rolling enable significant reduction without cracking, outperforming conventional basal-textured sheets [67].

In Mg alloys, dynamic recrystallization (DRX) involves two main mechanisms: discontinuous DRX (DDRX), which happens at twin and grain boundaries, and continuous DRX (CDRX), which involves subgrain rotation. In Mg-RE alloys, twin boundaries formed during deformation promote DDRX, resulting in very fine grain sizes of about 3.4 µm after hot extrusion [68]. In Mg–Zn–Y alloys with Long-Period Stacking Ordered (LPSO) phases, DRX tends to start within twins and near grain boundary LPSO regions, with the DRX volume fraction increasing from 3.5% to 48.8% as strain rises from 36% to 74% [69]. Smaller initial grain sizes in these alloys reduce the deformation energy needed and lead to changes in slip patterns during deformation [70]. Recrystallization in AZ31 alloys happens mainly in deformation bands or around twin interactions, affecting grain size and texture uniformity [71].

Mg alloys primarily consist of Al and systems that do not include Al and Mg alloys, and more seldom, they can be met with RE metals [72]. The system based on Al is referred to as AZ (Mg–Zn–Al) and the AM (Mg–Mn–Al) series [73]. The low-melting intermetallic phase Mg_17_Al_12_ contributes to limited creep resistance at operating temperatures under 120 °C [74,75]. Likewise, there exist the AE (Mg–Al–RE) and AXJ (Mg–Al–Ca–Sr) series [50,76,77]. The AE series primarily contains rare earth elements, which provide significant creep resistance due to the presence of rare element precipitates at the grain boundaries [50,78,79,80]. These alloys function at temperatures around 180 °C. The Al-free systems primarily consist of Zr, identified as ZK (Mg–Z–Zn), WE (Mg–Zr–Nd–Y), QE (Mg–Nd–Zr–Ag), ZE (Mg–Zr–Zn–RE), and the Elektron 21 (Gd–Mg–Nd–Zr) series [50,81]. These alloys can operate at temperatures near 300 °C.

Grain boundaries in fine equiaxed grains enhance strength in wrought Mg alloys (according to a law: σ ∝ d^−1^/^2^). A comprehensive review of Mg–Al, Mg–Zn, and Mg–RE wrought alloys confirms that grain refinement, precipitation, and texture hardening are the three main strengthening mechanisms; RE additions are particularly effective in weakening basal texture and enabling non-basal slip [82]. Solid-solution mechanisms in RE, Zn, Al, and Mn in α-Mg moderately contribute to AZ, ZK, WE, and ZE strength. Precipitation hardening from β-Mg_17_Al_12_, MgZn_2_, β′ plates, and Mg_3_Al nanoparticles enhances AZ, ZK, and Mg–Li–Al strength. β-Mg_17_Al_12_ precipitates discontinuously at grain boundaries and continuously in the matrix. Dynamic aging precipitation of granular Mg_17_Al_12_ during multi-directional forging of AZ80 produces inhomogeneous fine-grain/coarse-grain microstructure; these precipitates pin DRX grains [83]. In BCC Mg–Li–Al alloys (lightest structural alloys), semi-coherent D0_3_-Mg_3_Al nanoparticles precipitate during rapid cooling, providing immediate strengthening; prolonged aging causes coarsening and coherency loss, reducing strength. Controlling lattice mismatch and volume fraction of Mg_3_Al nanoparticles enables one of the highest specific-strength engineering alloys ever reported [84]. LPSO phase in 18R/14H structures contributes significantly to strength in Mg–Zn–RE alloys. Kobe Steel/Kumamoto University patents describe Mg–Zn–RE alloys (0.5–3 at.% Zn, 1–5 at.% RE) with lamellar LPSO + α-Mg microstructures. The LPSO phase has curved/bent morphology with divided regions containing α-Mg grains ≤ 2 µm, enabling superior mechanical properties without special production equipment [85]. Kumamoto University reported that Ca additions (x ≤ 0.5 at.%) to Mg–Zn–RE (Gd, Tb, Tm, Lu, or Y) LPSO alloys raise ignition temperature to ≥800 °C while preserving LPSO mechanical properties [86]. Quasicrystal/i-phase dispersion in Mg–Zn–RE enhances the strength of such Mg alloys. Toyota’s patent covers Mg–Zn–X (X = Zr, Ti, Hf) alloys in which Mg–Zn–X quasi-crystals and approximant crystals are dispersed as fine particles in the Mg matrix, improving high-temperature strength without expensive RE elements [87]. Basal texture alignment enhances directional strength in Mg-based wrought sheets/extrusions. National Institute for Materials Science (NIMS) patents a fine-grained (≤1.5 µm) Mg alloy containing 0.03–0.54 at.% RE/Group 2 solute atoms (Ca, Sr, Y, La, Gd, Nd, etc.) segregated to grain boundaries at 1.5–10× the grain interior concentration, simultaneously achieving high strength and ductility [88]. Al–Zr–B master alloy (5–20 wt.% Zr, 0.5–4 wt.% B, balance Al) acts as a potent grain refiner for both casting and wrought Mg alloys via heterogeneous nucleation [89]. An earlier method used graphite + MnO_2_ additions to Mg–Al–Mn melts to refine cast grain structure [90].

In the Mg–Al system, the β-Mg_17_Al_12_ phase controls strengthening and serves as the grain boundary phase. In the Mg–Zn system, the phases MgZn, MgZn_2_, and Mg_7_Zn_3_ control precipitation hardening. In Mg–Zn–Y alloys, tailoring microstructure by solutionising at 480 °C (to dissolve i-phase), extruding at ~430 °C, and low-temperature aging yields tensile YSs > 370 MPa at 20 µm grain size; realized by chill casting + low-temperature extrusion (~250 °C) produces ultrafine grains (<1 µm) with YSs up to 400 MPa and 12–16% elongation [91]. A new Kumamoto University patent covers Mg–Zn–Y alloys containing Mg_3_Zn_3_Y_2_ and/or Mg_3_Zn_6_Y phases but deliberately excluding LPSO, targeting the combined high-strength + high-thermal-conductivity niche [92]. In the Mg–RE (Gd, Y, Nd) system, precipitation on non-basal planes is controlled by β″/β′/β plates and the Mg_5_RE, Mg_24_RE_5_ phases. In the Mg–Zn–RE system, LPSO (18R, 14H) i-phase phases offer increased strength and ductility. CALPHAD-based phase diagram calculations for Mg–RE multicomponent systems confirm that combined LPSO + precipitation networks effectively impede dislocation motion on both basal and non-basal planes, and that LPSO phases suppress precipitate coarsening, providing good thermal stability [93]. Mg–Lu–Ce–Al–Ca–Cu–Ni alloys with LPSO phases (Mg–Lu–Al and Mg–Ce–Al) and high-temperature phases (Lu_5_Mg_24_, Mg_2_Cu, Mg_2_Ni) achieve dissolution rates of 30–100 mg·cm^−2^·h^−1^ in 3% KCl at 93 °C for oilfield plug applications [94]. In Mg–Al–La die-casting alloys, aberration-corrected STEM + DFT reveals that the strengthening η-Al_3_La phase is metastable and transforms through an intermediate ε-Al_2_._12_La_0_._88_ phase to α-Al_11_La_3_ during annealing at 250 °C. A newly identified monolayer twin defect provides the nucleation site and fast diffusion path for this transformation, with the consequence that plasticity and creep resistance deteriorate [95]. In the Mg–Li system, the BCC matrix combined with the D0_3_-Mg_3_Al phase results in ultra-light, precipitation-hardened alloys.

Mg is inherently active (standard electrode potential ≈ −2.37 V vs. SHE), and its corrosion behavior is unusually complex and is affected or evaluated by

- Negative difference effect (NDE). Hydrogen evolution increases with anodic polarization—the opposite of most metals—due to univalent Mg^+^ intermediates [96].
- Micro-galvanic coupling. β-Mg_17_Al_12_ in AZ alloys acts as a galvanic cathode relative to the α-Mg matrix. Y additions to AZ63 transform continuous Mg_17_Al_12_ networks into discrete Al_2_Y particles, alleviating micro-galvanic corrosion; additionally, Y promotes Al_2_O_3_ enrichment in the surface oxide and forms a more compact, uniform corrosion-product layer [97].
- Surface protection. Plasma electrolytic oxidation (PEO) is the leading surface treatment; smart PEO + inhibitor + polymer composite coatings reduce corrosion current density by three orders of magnitude in 3 wt.% NaCl and show antibacterial activity against MRSA [98].

Corrosion behavior can be evaluated using scanning probe electrochemistry. Local electrochemical techniques (SVET, SIET, SECM, LEIS, SKP) with spatial resolution revealed the heterogeneous corrosion activity of bare and coated Mg surfaces and are now the standard toolkit for mechanistic corrosion studies [99].

Baoshan Steel patented Mg–Ge–Zn alloys (0.01–1.2 wt.% Ge, 0.01–1.2 wt.% Zn, with optional Mn/Ca/Zr/Sr/Gd) processed by smelting followed by solid solution and by extrusion (180–350 °C, extrusion ratio 10:1–30:1) targeting simultaneous strength and corrosion resistance [100].

Biomedical Mg–Nd–Zn–(Ca)–(Zr) alloy (1.0–4.5 wt.% Nd, 0.2–2.0 wt.% Zn) produced by vacuum semi-continuous casting followed by solid solution and extrusion achieved a balance of controlled degradation rate, non-toxicity, and sufficient strength for vascular stent applications [101].

It can be noticed that for Mg alloys, slip and twinning fundamentals are established, demonstrating that HCP restricts slip; while twinning is crucial, it leads to asymmetry and cracking. Texture control (RE alloying) is in a mature, advancing state and demonstrates that RE texture diminishes the basal fiber strength and enhances formability. DRX and grain refinement are in an advanced state and show that DDRX/CDRX are well-characterized; LPSO phases improve nucleation.

Precipitation (Mg–Al, Mg–Zn) occurs in the mature state and demonstrates that β-Mg_17_Al_12_ and MgZn_2_ are well-defined. LPSO-phase alloys are progressing in commercial applications and show the greatest strength–ductility and possess a dynamic IP landscape. Ultralight Mg–Li alloys are in a developmental phase and show a newly understood D0_3_-Mg_3_Al precipitation. Corrosion mechanisms are under active investigation, showing that NDE and localized corrosion remain not fully understood. Biomedical biodegradable alloys are in a clinically advancing phase and show a controlled degradation rate as a crucial design factor. Flame-resistant alloys are in a patented, scalable state and show a combination of Ca + LPSO. Their ignition temperature is ≥800 °C.

Typically, Table 1 presents the impact of different alloying elements on the mechanical and microstructural properties of Mg-based alloys. Mg alloys are manufactured and utilized for commercial applications [21,102]. Table 2 presents certain alloys along with their physical, mechanical, and corrosion characteristics. Certain possible areas for application have been proposed.

## 3. Welding Types of Mg and Its Alloys

The assembly of components made from Mg or its alloys to form complex and detailed shapes is increasingly required in the engineering design and manufacturing of diverse structures utilized in numerous industries. Joining falls into the categories of mechanical and liquid–solid-state processes. Mechanical joining comprises fasteners, bolts, and nuts, as well as screws. Mechanical joints do not involve temperature, so there is no phase transformation or microstructural evolution. Joining parts in liquid and solid states encompasses welding, adhesive bonding, brazing, and soldering [50,103,104]. Renish et al. [105] conducted a short overview of the welding of Mg alloys utilizing different welding methods, assessing parameters to evaluate the optimal welding condition and to analyze the material strength at the weld junction. Additionally, factors like cracks at welded joints, hardness, grain size, and the development of intermetallic compounds (IMCs) are also addressed.

### 3.1. Fusion Welding

**Figure 1:**
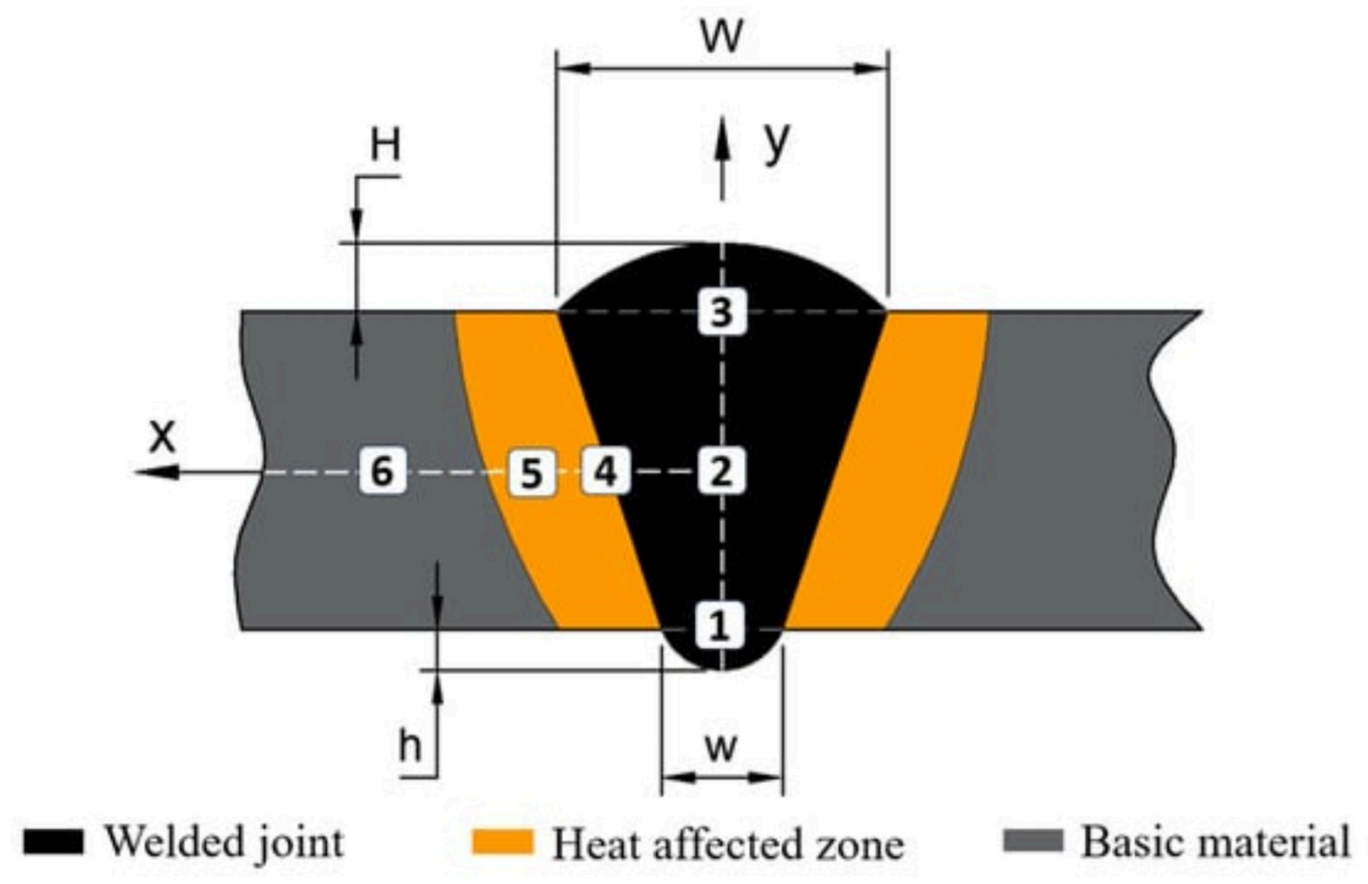
Various zones on a typical butt weld showing the HAZs and the FZ comprising welded filler material [106]. 1—the bottom root of the weld (welded filler material), 2—the middle zone of the weld (welded filler material), 3—the upper part of the welded seam (welded filler material), 4—the transition zone between the BM and the filler material, 5—HAZ, 6—BM.

Fusion welding involves applying heat to unite two or more materials or mechanical parts by melting them to their melting point [50]. This method utilizes filler or no filler metal based on the material, the welding design, and the assembly of the joint. This method does not necessitate any welding pressure to produce high-quality welds [106]. The process of phase transformation caused by excessive heating results in the creation of an FZ (weldment) and a heat-affected zone (HAZ) (Figure 1), which can negatively or positively influence mechanical properties and structural integrity [106]. The FZ is a microstructure formed by the solidification of deposited metal and a section of the molten parent or base metal (BM) during the welding process. The HAZ is the region near the FZ that remains solid but experiences microstructural changes due to the very high temperatures linked to the welding process [106]. Fusion welding techniques comprise electron beam welding (EBM) [107], arc welding [108], laser welding [109], gas metal arc welding (GMAW) [105,110], and hybrid welding [42]. Various alloying elements (Table 1) affect the properties of various Mg-based alloys (Table 2).

**Table 1:** Effect of alloying elements on specific properties of Mg-based alloys.

Alloy Element	Density [kg/m^3^]	Melting Temperature [°C]	Specific Heat [J/kg·K]	Coefficient of Thermal Expansion [×10^−6^ 1/K]	Thermal Conductivity [W/(m·K)]	Refs.
**Mg**	1740	650	1020	8.2	155–160	[111,112,113,114,115,116,117,118,119]
**Properties**	Casting behavior: The dimension of eutectic silicon particles rises with increased Mg concentration. The overall Mg concentration also affects the distribution and size of other phases, including the β-phase Mg_17_Al_12_. Mechanical properties: increases YS and tensile strength (TS) through mechanisms like solid solution strengthening and grain refinement by forming IMCs, but excessive Mg can lead to brittle intermetallic, increased porosity, and reduced ductility and TS. Corrosion properties: The high reactivity of Mg renders Mg alloys susceptible to rapid corrosion, frequently resulting in localized corrosion, pits, and considerable metal loss. The influence of Mg on corrosion is intricate: although its presence heightens the risk of corrosion, the quantity and arrangement of additional elements such as Al or Ca in an alloy can affect its corrosion resistance. For instance, incorporating Al to a specific percentage can enhance the microstructure and boost strength without compromising corrosion resistance, whereas a larger quantity can expedite corrosion. Likewise, the configuration of intermetallic phases, like Mg_2_Ca, significantly influences performance, as a more fragmented distribution offers superior resistance compared to a continuous arrangement.					
**Ag**	10,500	962	2370	18.92	429	[50,120,121,122,123,124,125,126,127,128,129,130]
**Properties**	Casting behavior: It can enhance the grain size of as-cast alloys, resulting in a more uniform microstructure. It aids in creating finely dispersed second-phase particles, which can improve hardness and strength. The creation of stable Mg_4_Ag particles can enhance creep resistance at elevated temperatures. It can enhance and speed up the aging process, enabling alloys to reach peak hardness more quickly. In certain situations, Ag can diminish porosity and vulnerability to cracking during casting and later heating. The inclusion of Ag can reduce the corrosion resistance of Mg alloys, particularly when Ag-rich intermetallics develop. Certain Mg–Ag alloys show a greater tendency to crack during heat treatment than alloys lacking Ag. Mechanical properties: Ag improves elevated-temperature tensile and creep resistance when alloyed with rare earth elements. Corrosion properties: Ag is detrimental to corrosion behavior.					
**Al**	2700	660.3	900	22.87	237	[50]
**Properties**	Casting behavior: Al increases casting behavior and leads to microporosity. Mechanical behavior: Solid solution and precipitation hardening. Corrosion properties: Minimum effect observed.					
**Ca**	1550	842	630	22.27	201	[50]
**Properties**	Casting behavior: Excellent grain refining; it suppresses oxidation. Mechanical behavior: Ca improves creep resistance. Corrosion behavior: Ca demonstrates poor corrosion resistance.					
**Li**	534	180.5	3489	46.56	85	[50]
**Properties**	Casting behavior: Li increases evaporation and burning behavior. Mechanical behavior: Solid-solution hardening effect; it reduces density and increases ductility. Corrosion behavior: Li decreases corrosion resistance.					
**Mn**	7476	1246	480	23.61	7.81	[50]
**Properties**	Casting behavior: Precipitation of fine Fe–Mn precipitates. Mechanical behavior: Mn increases creep resistance. Corrosion behavior: Mn improves corrosion resistance with Fe in the alloy.					
**Rare earth**	Gd: 7900, Ce: 6689, Nd: 7010, Y: 4472	Gd: 1313, Ce: 798, Nd: 1021, Y: 1526	Gd: 240, Ce: 192, Nd: 190, Y: 298	Gd: 9.4, Ce: 6.3, Nd: 9.6, Y: 10.6	Gd: 11, Ce: 11, Nd: 17, Y: 17	[50,117,131,132,133]
**Properties**	Casting behavior: REs improve castability and reduce microporosity. Mechanical behavior: Precipitation hardening and solid solution strengthening at both room and elevated temperatures, improving tensile and creep properties. Corrosion behavior: REs enhance corrosion resistance through reactions with impurities, generating protective passive layers, and producing IMCs that diminish micro-galvanic corrosion. The precise impact differs based on the type and quantity of REE; however, elements such as gadolinium (Gd) and cerium (Ce) create stable oxide and intermetallic layers that protect the Mg matrix. Additional REEs such as neodymium (Nd) and yttrium (Y) aid in eliminating impurities, improving grain structure, and stabilizing surface films, all of which boost corrosion resistance.					

**Table 2:** Commercial Mg-based alloys with specific property characteristics.

Alloy	Characteristics	ρ [kg/m^3^]	m.p. [°C]	c [kJ/kgK]	α_T_ [μm/mK]	λ_T_ [W/mK]	UTS [MPa]	YS [MPa]	E_t_ [%]	HB	E [GPa]	KV [J]	Refs.
**AM20**	Good impact strength + ductility	1.75	435–638	1.02	26	94	190	90	12	45	45	18	[134]
**AM50**	Suitable for high-pressure die castings	1.77	435–620	1.02	26	65	210	125	10	60	45	18	[134]
**AS41B-F** **AS21**	Creep resistant up to temperatures of 150 °C	1.77	NA	NA	NA	66.9–68	200–250	120–150	3–12	55–80	45	2.6–2.7	[135]
**AE42**		1.79	590–625	1.02	26.1	84	230	145	10	60	45	5	[134]
**AZ31**	Weldable + intermediate strength + excellent plasticity	1.77	605–630	1.05	26	96	260	200	15	49	44.8	4.3	[136]
**AZ61**	High strength + weldability	1.8	360–650	1.05	26	70	310	230	15	60	44.8	4.1	[136]
**AZ63**	Great RT ductility + toughness + strength	1.83	455–610	1.01	26.1	77	200	97	6	50	45	NA	[137]
**AZ81**	Suitable for pressure die casting	1.8	>421	1.02	27.2	51.1	275	83	15	55	45	NA	[138]
**AZ91**	Suitable for sand and die castings	1.81	435–598	1.02	26	51	240	160	3	70	45	6	[134]
**EZ33**	Castable + weldable + creep-resistant up to 250 °C	1.8	545–645	0.97	26.4	99.5	160	145	2	50	45	NA	[139]
**HK31**	Sand castable + weldable + creep-resistant to 350 °C	1.8	590–650	1.02	26.8	92	250	190	15	58	45	0.79	[140]
**QE22**	Weldable + high proof stress to 250 °C	1.82	640	1.04	26.7	113	260	195	3	49	45	NA	[141]
**QH21**	Weldable + good proof stress to 300 °C + creep-resistant	1.83	535–640	1.02	26.7	113	275	210	4	80–95	45	NA	[142]
**WE43**	Weldable + non- corrosive	1.8	540–640	1	26.7	51.3	250	150–162	2	70–105	44.2	3.8	[143]
**WE54**	Elevated and ambient temperature strengths	1.8	545–640	1	24.6	52	280	225	2	80–90	44–45	NA	[144]
**ZC63**	Pressure-tight castable + high temperature strength + weldable	1.87	465–635	1	26	122	210	125	4	60	45	NA	[145]
**ZE41/** **ZK51/ZK61**	Sand-castable + good ambient temperature strength + ductility	1.84/1.83/1.83	532–638/360–650/520–635	0.97/1.02/1.02	15.1/26/27	24/110/120	218/271/271	140/175/175	4.5/3.5/3.5	55–70/70–80/68	44.12/45/44–45	NA	[139]

ρ—density, m.p.—melting range, c—specific heat, α_T_—coefficient of thermal expansion, λ_T_—thermal conductivity, UTS—ultimate tensile strength, YS—yield strength, ε_t_—elongation, HB—Brinell hardness, E—elastic modulus, KV—Charpy impact, RT—room temperature.

Zibo [146] stated that most Mg alloys can be joined using gas–tungsten arc welding (GTAW) and gas–metal arc welding (GMAW). Singh et al. [147] pointed out that welding Mg alloys faces challenges like high reactivity with gases, hot cracking, porosity, and thermal distortion. While conventional GTAW methods yield satisfactory results, they may not meet high-precision requirements. Innovations like pulse-modified and hybrid GTAW techniques improve heat input control and reduce defects. Key factors, such as shielding gas choice, filler materials, and heat input, are essential for quality welds. Advancements in AI, real-time monitoring, and automation enhance GTAW’s accuracy and efficiency, with future trends including its integration with additive manufacturing for diverse applications.

#### 3.1.1. Gas Tungsten Arc Welding (GTAW) or Tungsten Inert Gas (TIG) Welding

The TIG welding process involves creating and utilizing heat generated by an electric arc. This process can be called gas tungsten arc welding (GTAW). Welding utilizes non-consumable tungsten electrodes. The neighboring metals are fused together, and the liquid pool is protected with inert gases like Ar, He, and N_2_ to avoid contamination from the atmosphere. Benefits of utilizing GTAW include the capability to weld with or without filler metal and the ease of transitioning between automatic and manual mode (eliminating the need for consumable electrodes). This results in robust, neat, and visually pleasing joints with reduced imperfections [50]. A significant limitation of GTAW is the occurrence of pores in the welds, which undermines weld quality and integrity. Coarse grains are likewise produced in the weldment. These coarse grains serve as locations for dislocation movement, facilitating the rapid spread of cracks. The procedure is also lengthy in comparison to other welding techniques and necessitates specialized training [50,148]. Table 3 contains some information about TIG welding techniques applied to Mg alloys.

Yogesh Dubey et al. [149] examined the influence of TIG welding on Mg and Al alloys, as well as stainless steel, along with its effect on mechanical properties and microstructural control, leading to enhanced characteristics of the alloys. The TS of weld metal was usually inferior to that of the BM in Al alloys. The hardness and crystal grains in the FZ were extremely fine and close to those of the BM in the Mg alloy. The fatigue life of weld material made from stainless steel was noted to be less than that of the BM. The presence of metal oxides was the main factor that affected the longevity of the weld.

According to [150], grain sizes are essential for enhancing the mechanical attributes of alloys and weldments.

The TS of LA141 Mg alloy reached approximately 95% of the BM after welding with a current of 60 A and a speed between 2.8 and 3.2 mm/s [151]. The butt joints exhibited small grain sizes, enhancing the strength. Positioning a magnetic arc oscillator in the welding path leads to the refinement of dendritic microstructures through mechanical mixing within the weld zone.

For an AZ31B gas-welded alloy, a magnetic oscillatory frequency between 1 and 3 Hz significantly enhanced tensile characteristics [152]. The tensile properties showed a steady increase with the rise in oscillation arc frequency from 1 to 1.5Hz, reached a maximum at 2 Hz, and then a sharp decrease was noted for frequencies exceeding 2 to 3 Hz. Low oscillatory frequency leads to a broad weld bead with reduced penetration because of diminished thermal and mechanical disturbances. The shift from fine grains to coarse, elongated grains at low oscillation frequencies and above 2 Hz leads to inferior tensile properties. The peak tensile characteristics observed at 2 Hz are marked by small grains and uniformly distributed precipitates.

**Figure 2:**
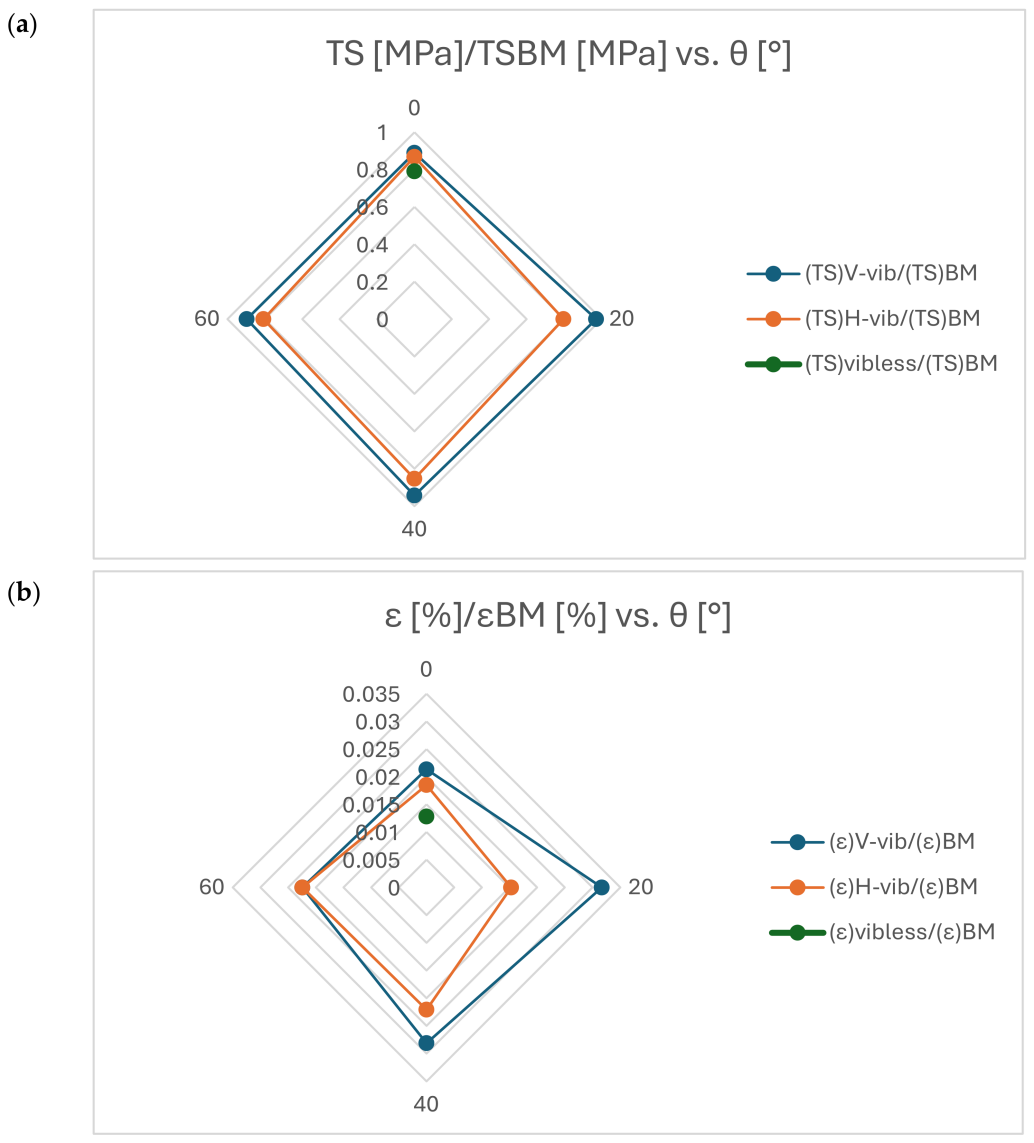
Effect of vibration parameters on (**a**) UTS of AZ31 alloy and (**b**) elongation of AZ31 alloy during TIG weldment (based on data from [50]).

Dynamic grain refinement via ultrasonic vibration-assisted treatment enhances the mechanical properties of welded joints [153,154]. According to [50], during the welding of the AZ31 alloy, which occurred at 20 cm/min and at 100 A, the grain size was markedly reduced under the conditions of vibration. The impact of vibration parameters on the phase ratio of the intermetallic β-Mg_17_Al_12_ in the welding zone was slight, yet there was a noticeable enhancement in micro-hardness and tensile characteristics (Figure 2). Vibration impacts the thickness of the weld as well. The influence of vibration on the microstructure and mechanical characteristics of the weld joint is influenced by the level of energy supplied. This also depends on the amplitude, vibration orientation, depth, and direction of the groove. Vertical vibrations significantly impacted thick weldments, affecting performance based on processing parameters like amplitude and groove angles. The effect of vibration parameters (V—vertical and H—horizontal directions) on UTS and elongation of AZ31 TIG weldment was shown in Figure 2.

Babu et al. [148] studied how welding speed affects the microstructure, hardness, and tensile properties of AZ31 alloy GTA welds. Higher speeds resulted in larger equiaxed zones and improved strength, hardness, and ductility due to reduced heat input. Yürük [155] assessed the impact of welding speed on AZ31 alloy using TIG welding, exploring three distinct speeds. Microstructure and mechanical properties were analyzed through macro and microstructural examinations and tensile, hardness, and bending tests. Higher welding speeds reduced heat input, affecting weld bead width and grain size. Hardness was highest in the weld metal (69.2 HV) and lowest in the HAZ. TS increased with speed, peaking at 175.82 MPa for sample S3. Bending tests showed no defects in the welds. Qin et al. [156] studied TIG and A-TIG welding of AZ61 and AM60 alloys. They also used a mathematical model for the welding pool using a transient moving heat source, with a simulated temperature field. Optimal conditions for TIG welding were identified at 115 A current and 180 mm/min speed, resulting in a defect-free welding seam. Notably, AM60 has higher thermal conductivity than AZ61, leading to an asymmetrical molten pool. A-TIG welding with activating flux produced an ingot-shaped surface with significant penetration, while TIG welding resulted in a broader, level surface with shallow penetration. Assar et al. [157] (Table 3) studied the effect of heat input in GTAW on the microstructure and mechanical properties of AZ91 alloy. They observed a fine microstructure in the weld, with increased α(Mg) phase size and partially melted zone thickness. Welded samples showed lower TS than the BM, decreasing with heat input, with fractures occurring primarily in the partially melted zone.

**Table 3:** Some findings for classical TIG and activating TIG welding of Mg alloys.

TIG Version	Welding Parameters	Materials	Findings	Refs.
TIG welding and activating TIG (A-TIG)	The Ar shielding gas with a flow rate of 7.5 L/min. Welding voltage of 20 V, currents 60–120 A. TiO_2_ as the active agent.	AZ61-AM60, 3 mm thick	Optimal conditions at 115 A and 180 mm/min produced defect-free seams. Thermal conductivity differences caused asymmetric molten pools, with A-TIG creating deep penetration, and TIG yielding a shallower, broader seam, along with two notches on A-TIG weld edges.	[156]
TIG welding	2.4 mm diameter W electrode with 2 wt.% La, 30° angle tip and ceramic nozzle with a diameter of 10 mm. The Ar shielding gas with a flow rate of 10 L/min.	AZ91, 10 mm thick	TIG welding of AZ91 alloy results in a fine microstructure of α(Mg) and Mg_17_Al_12_ phases. Increased heat input enlarges the α(Mg) phase and partially melted zone, reducing TS and promoting failure in the partially melted zone.	[157]
TIG welding, butt	WT20 electrode of the diameter 3.2 mm, welding current 120 A, arc voltage 14 V, linear energy of the arc 3 kJ cm^–1^. Filler wires of a diameter of 2.4 mm, of the composition close to the investigated materials.	Mg alloys, 20 mm thick	Welds of QE22, ZRE1, and WE43 alloys with rare earths showed similar corrosion resistance to the BM in NaCl, but lower than AZ91.	[158]

Jadhav et al. [159] conducted TIG welding on AZ31B alloy joints using 75 A peak current and 50 A bottom current with specific gas flow rates. BM strength was 273.37 MPa, decreasing to 193.70 MPa and 231.13 MPa for samples 1 and 2, respectively, indicating a 22.29% strength reduction. TIG welding is widely used among the current welding techniques because of its flexibility in operation and affordability [160,161,162]. Liu et al. [160] conducted a feasibility study on TIG welding for Mg–8Li–3Al–2Zn–0.5Y alloys, which revealed effective joint quality with minimal defects at various currents. Enhanced phase size improved mechanical properties, with UTS and YS at 224.8 MPa and 177.4 MPa, respectively. Successful Li management confirmed the welding method’s viability for Mg–Li alloys. Wang et al. [161] noted that traditional Mg–RE alloys underwent significant grain coarsening in the heat-affected zone during TIG repair welding, adversely affecting tensile properties. Researchers studied how welding currents influenced the microstructure and mechanics of Mg–4Y–3Nd–1.5Al alloy joints. Currents between 150 A and 225 A produced defect-free, visually appealing joints, with enhanced grain size in the molten pool. Higher currents initially reduced grain size before increasing it. However, semi-continuous eutectic structures near grain boundaries could promote cracking, slightly lowering tensile characteristics. The joint repaired at 200 A recorded UTS, YS, and EL of 238 MPa, 166 MPa, and 8.8%, showing minor reductions compared to the as-cast alloy. As noted in [163,164,165], welding Mg/Ti hybrid structures has faced numerous challenges because of their physical and metallurgical incompatibilities. Various joining techniques have been utilized to connect Mg/Ti, and in many instances, achieving a metallurgical bond between them involved using an intermediary element at the interface or the mutual diffusion of alloying elements from the BMs. The development of a reaction product (as a solid solution or IMC) at the boundary between Mg and Ti leads to the creation of a metallurgical bond. Nonetheless, the performance of the joints and the bonding at the interface relies on the reaction product(s) created. According to [165], commonly employed arc welding methods for creating Mg/Ti welds, including metal inert gas (MIG) and tungsten inert gas (TIG), seem to be controversial. The effectiveness of this joining method in creating a metallurgical bond between Mg and Ti stemmed from the incorporation of alloying elements from the BMs, filler metal, or coating layers. TIG welding is preferred over MIG welding because the TIG arc remains stable at low currents, enhancing the potential for joining thin components. It provides extra advantages like adaptability, high effectiveness, excellent joint quality, and can connect different metals such as Mg/Ti. While arc welding Mg/Ti alloys, various researchers noted that a joint was created that was both welded and brazed. Incorporating an appropriate interlayer may enhance the adhesion and nucleation of Mg on the Ti substrate. Nonetheless, the reaction taking place at the interface has not been completely comprehended. MIG and TIG both appear to face challenges with low welding speeds and high heat input, leading to significant defects in the weldment, including voids, elevated residual stresses, and distortion [165].

Eftekhar et al. [162] studied semisolid AXE622 alloy welded by TIG, finding optimal TS at HI21, larger HAZ grain sizes, and decreased hardness with increased heat input. According to [41], the welding parameters for Mg–RE alloys, such as EV33 alloys, were systematically refined based on their unique material properties. An in-depth study was carried out on the microstructural changes in the EV33 alloy during TIG welding [41]. Grain refinement in the FZ was achieved using a 10 Hz pulse current, resulting in a joint efficiency of 85%. However, the TS of the TIG-welded EV33 alloy joint is still insufficient for aerospace structural applications. Its strength–ductility combination is notable, with UTS at 220 ± 14 MPa and EL at 11.9 ± 1.2%. Current (PWHT) PWHT research has primarily concentrated on processed Mg–Gd–Y [166]. The findings highlight the significance of PWHT; however, it is important to recognize that these results primarily relate to wrought alloys and may not be directly applicable to sand-cast materials. A key issue stems from the notable microstructural distinctions between sand-cast and wrought BMs. Sand-cast BM has a significant amount of coarse, network-shaped eutectics that partially melt and re-solidify during welding, resulting in continuous brittle phases along the grain boundaries in the HAZ, which is also referred to as the partially remelted zone in certain contexts. In comparison, the quickly solidified FZ consists of smaller and more scattered eutectics [162]. This microstructural variability complicates the attainment of a consistent solutionized or aged microstructure across the joint with traditional PWHT schedules. Wang et al. [167] reported that post-weld heat treatments (PWHT) impacted the microstructure of TIG-welded Mg–3Nd–3Gd–0.2Zn–0.5Zr (EV33) alloy joints, showing inadequate thermal stability in the FZ and susceptibility to abnormal grain coarsening (AGC). Increasing the T4 temperature significantly enlarges grain size and reduces residual phases. A novel multi-step T4 process minimizes AGC, optimizing mechanical properties. A model for grain growth and failure mechanisms is proposed. Meng et al. [168] found that laser bionic treatment improved the mechanical properties of AZ31B alloy TIG welded joints, enhancing TS by 23% and elongation by 105%, despite a slight decrease in YS. Zhang et al. [169] created a novel AC/DC hybrid inert gas TIG (MIX-TIG) to enhance the welding quality of AZ31B alloy TIG. Under the same welding conditions, the average grain size and number of β-phases in the MIX-TIG were lower than in the AC-TIG. In comparison to AC-TIG welded joints, the strain of MIX-TIG welded joints rose by approximately 24%. Xu et al. [170] reported that TIG welding of Mg alloys showed that Mg atoms replace Ar in the arc, affecting discharge. The lowest electron temperature occurred near the anode. Wang et al. [171] suggested a new reinforcement method for fusion-welded Mg alloy joints, using fine, uniform grains and particle-reinforced structures. Powder feeding alongside fusion welding and friction stir processing resulted in optimal microstructures. The molten weld grain size decreased from 193.5 μm to 15.9 μm, enhancing TS by 70.2 MPa and elongation by 136%. Jin et al. [172] examined the TIG welding of 2.15 mm LAZ931 Mg–Li alloy, which showed a narrow melt width of 9 mm and formation of θ and γ phases. HAZ had the highest strength due to the θ phase, with maximum hardness at 87.6 HV; joint TS reached 163.2 MPa with 23.9% elongation. Subravel et al. [173] investigated the tensile and microstructural effects of GTA welding techniques on AZ31B Mg joints. Techniques included pulsed current, constant current, and magnetic arc oscillation. Magnetic arc oscillation produced joints with superior tensile properties due to finer grains, increased surface hardness, and evenly distributed precipitates in the transition area. Radović and Marinković [174] studied the sensitization effects on intergranular corrosion of TIG-welded AlMg6Mn. Using SEM and corrosion tests, they found increased IGC susceptibility, with mass losses of 106.7 mg/cm^2^ in welded samples compared to 70.7 mg/cm^2^ in BM. Kumar Maurya et al. [175] investigated FSW and TIG welding on 5 mm Mg alloy plates (AZ31B and ZM21). FSW parameters included 2100 rpm speed and 140 mm/min. Results showed AZ31B was more prone to corrosion than ZM21, with optimized parameters achieving 208 MPa TS without defects. As stated in [176], inadequate weldability of Mg alloys poses a significant obstacle to their potential application in safety-critical areas. The formation of porosity, engineering of grain structure, cracking during solidification, liquation, and liquation cracking are the primary metallurgical challenges to achieve dependable and strong fusion welds in Mg alloys. According to [177], the activated flux has minimal adverse effects on welded joint properties. During TIG welding of AZ91 alloy, applying a longitudinal alternating magnetic field and NiCl_2_-activated flux improved weld penetration and efficiency. Optimal parameters achieved TS of 385 MPa, elongation of 13.3%, and microhardness of 67 HV with 3 mg cm^−2^ flux. The ideal flux was 2 mg cm^−2^, with no significant phase composition impact. The combined effects enhanced crystallization conditions and refined grain size. Cooke et al. [178] highlighted the importance of welding techniques for Mg alloys to ensure strong bonds. Methods include mechanical fastening, adhesive bonding, and fusion welding, with TIG welding favored for its economy, versatility, and ability to achieve 94% shear strength of base Mg alloys. Sik [179] investigated the mechanical properties of Mg TIG and FSW on a 4 mm thick AZ31D alloy. FSW produced smoother weld beads with no distortion due to low heat input. Fractures initiated from the HAZ during bending fatigue tests, showing lower fatigue strength in welded samples compared to BM. TIG joint samples exhibited higher TS than FSW. Discontinuities increased with higher rotational speeds, impacting fatigue strength. FSW parameters can be adjusted for effective welding, particularly for Al alloys challenging for fusion methods. Zhang et al. [180] examined TIG and A-TIG welding on annealed AZ31 alloy, utilizing 2.4 mm AZ61 filler wire. Both techniques produced smooth, consistent welds without defects. Full back penetration was achieved, indicating effective gas protection, while the active agent enhanced back penetration in the welds. Wang et al. [181] studied Al–Mg–Mn–Er alloy sheets welded by TIG with filler wires. Their findings indicated the weld seam consisted of equiaxed crystals, lacking conventional epitaxial solidification. A finely grained area was present near the fusion line, while the heat-affected zone had a recrystallized structure. The weld zone had primary Al3Er precipitates exhibiting smaller, uniform distributions. Microhardness was lower in the weld and heat-affected zones, with maximum TS at a heat input of 218 J/mm. Fractures occurred in the weld region, showing ductile features.

Summarizing, TIG welding of Mg alloys is extensively used in the automotive industry due to its high-quality joints [50,180]. Key principles include using AC polarity to balance oxide cleaning and penetration, with Ar as standard shielding gas and specific filler materials like AZ61A. Pre-weld cleaning is crucial to minimize defects [180]. Process challenges such as oxide inclusion, burn-through, and cracking can be tackled by AC-TIG and using low-melting-point fillers [153,158,180,182]. A-TIG enhances penetration and speed through activating flux [180]. Typical parameters range from 60 to 200 A current and 135–1400 mm/min travel speed [50,158,180]. Post-weld heat treatment improves ductility and reduces stresses [50,180].

#### 3.1.2. MIG/GMAW Welding

The MIG/GMAW technique can be used to join various Mg alloys [40]. According to Tukur Aval et al. [104], achieving a reliable Mg alloy-to-steel connection through MIG welding is crucial. Nonetheless, the joining of Mg to steel via MIG is infrequently documented, such as with AZ31B/Zn-coated steel [40], AZ31B/Q235 featuring a Cu interlayer [40], and AZ31B/Aluminized steel [40]. Typically, incorporating an appropriate interlayer enhances the spreadability and nucleation of Mg on the steel surfaces. Cold Metal Transfer (CMT), a modified version of GMAW joining AZ31B Mg to both galvanized and bare mild steel sheets, demonstrated that welded brazed joints developed in each joint [40]. In general, research on Mg alloy/steel joints created through MIG welding is still at an early stage [40,104].

Nonetheless, GMAW is not widely applied for Mg alloys [50]. The GMAW process is often used to weld very thick Mg pieces [183]. Significant spatter, meaning the ejection of molten filler metal from the arc, has been a considerable problem. GMAW, known as metal inert gas (MIG) welding, utilizes a shielding gas like Ar, He, or a mixture of Ar/He to safeguard the molten metal.

GMAW is widely used for welding Al alloys [184] but less so for Mg alloys [40]. The wire-arc directed energy deposition (DED) process can be used for the fabrication of large and complex Mg alloy components to increase manufacturing efficiency and material utilization. The wire-arc DED processes can be realized using GMAW at a relatively high rate, but this process is less stable and prone to welding fume and spatter [185].

Das Neves [186] examined AZ61A alloy deposition via GMAW-based WAAM, influencing heat input (HI) and revealing internal flaws, deposition inconsistency, consistent grain size (17.49–22.93 μm), and microhardness decline with HI increase amid various challenges. Kossack [187] investigated GMAW for Wire Arc Additive Manufacturing (WAAM) of Mg alloys using AZ61a deposition wire. This technique produced multilayer walls, blocks, and hollow cylinders under varying input-energy rates (IER). The printed structures were evaluated for mechanical properties, microstructure, and porosity, revealing a YS of 120 MPa for multilayer walls and a decreased YS of 106 MPa at higher torch travel speeds (TTS). Fracture stresses ranged from 260 to 270 MPa, while larger defects reduced stress-at-fracture values in multi-row samples. Porosity was low, with some significant flaws noted. According to [188], Mg welding is limited by its unique properties. Shielded metal arc welding (SMAW) is unsuitable due to toxic fume generation and potential distortion. Oxyacetylene welding is also inappropriate, causing oxidation and combustion risks, leading to cracking and distortion. According to [189], surface preparation is crucial for reducing weld defects in Mg alloys, necessitating the removal of contaminants and oxide layers through grinding, brushing, or chemicals. Proper flux application is vital for flame welding. Preheating, determined by alloy thickness and restraint, typically ranges from 260 to 400 °C; thicker, low-restraint pieces may not require preheating. GMAW is common for thick welds, but GTAW is better for thin sections. To prevent cracking, groove welds should start at the center, and suitable preheating and management techniques should be used during welding. Yi et al. [185] explained that wire-arc direct energy deposition (DED) uses a heat source to melt metal wire, depositing it layer by layer on a substrate. A software system manages model slicing, path planning, and process parameters for effective operation. In contrast to the additive manufacturing (AM) method that utilizes metal powder as feedstock, wire-arc DED offers advantages such as improved material efficiency, greater environmental safety, and the elimination of the risk of Mg powder explosion hazards [190,191]. Based on the various types of heat sources, wire-arc DED methods can be classified into three distinct categories: Gas metal arc welding (GMAW)-based, Gas tungsten arc welding (GTAW)-based, and plasma arc welding (PAW)-based [185]. The deposition rate of GMAW exceeds that of plasma arc welding (PAW) and GTAW; however, the process is less stable and more susceptible to welding fumes and spatter. Cold Metal Transfer (CMT) is an advanced heat source created from GMAW, which accomplishes the retraction of the welding wire by reversing the servo motor through digital process management, thereby achieving a current-free transfer. CMT offers benefits such as low heat input, a stable welding process, and no spatter [185]; as a result, it is commonly utilized for wire-arc DED across different materials.

Summarizing, MIG/GMAW for Mg alloys is effective for automated production of structural components. It requires high skill and precise parameter optimization to manage challenges such as reactivity, low melting point, and porosity [40,50]. Optimal welding speeds range from 400 to 1400 mm/min, with higher speeds (above 8.3 mm/s) and consistent filler wire feed rates (over 110 mm/s) recommended [50]. Argon is the preferred shielding gas, while pulsed MIG techniques enhance control and reduce heat input [50]. Preheating (250–350 °C) allows for thicker sections of joined parts [50]. Advantages include productivity and deep penetration, though defects like hot cracking and microporosity are common [40,50]. Best practices involve thorough cleaning and maintaining a short arc length [192].

#### 3.1.3. Controversies Relating to GMAW and GTAW Applied to Mg Alloys and Discussion on the Choice Between Them

Research on the welding of Mg alloys using GMAW and GTAW contains conflicting findings about porosity formation, heat input, and mechanical properties, to some extent, as in the case of Al alloys [193,194,195]. In terms of porosity, some studies assert GMAW leads to more hydrogen and oxide contamination, while consensus suggests that the alloy’s high affinity for oxygen and hydrogen is the main issue, with shielding gas purity being crucial [50,147,196,197]. Regarding heat input, traditional studies favor GTAW for better thermal control, but recent findings indicate that advancements like CMT in GMAW provide lower heat inputs and reduced HAZs [147,185,194]. The debate on mechanical properties shows earlier claims favoring GTAW for defect-free welds; however, optimized GMAW conditions may yield comparable or superior TS and hardness. The introduction of hybrid techniques (GMAW–GTAW) combines advantages from both methods, as discussed further in a separate chapter [195,198,199]. Numerous reports discuss (relative to Mg alloys and to some extent to Al alloys) heat input and bead profiles [40,200,201], spatter [202,203,204], and porosity debates [196,205,206], emphasizing that GMAW’s high deposition rates suit automated production despite its initial spatter issues [145,204]. Lastly, discrepancies in welding parameters—such as WSs [50] and shielding gases—have led to varied claims on mechanical strength [207], with the choice of gases like Argon or Helium affecting heat input and potential hot cracking risks [201,207].

Interestingly, the TIG method is better than the MIG one for welding Mg alloys when high weld quality, precision, and esthetics are crucial. The TIG method excels in heat control and deep penetration, making it suitable for thin gauges, while the MIG method is better for high-volume production, thick sections, and long seams. The choice between these two methods is usually affected by at least four features, including

- Weld Quality and Metallurgy: The TIG method offers better arc stability and precise heat input, reducing burn-through risks on reactive Mg. It produces clean, porosity-free welds with minimal spatter. The MIG method, with a wider HAZ, is more prone to porosity and spatter. For dissimilar joints, the TIG method allows for careful control of brittle intermetallic compound formation.
- Material Thickness: For thin materials (<3 mm), the TIG method is ideal due to its focused arc and lack of continuously fed wire. The MIG method performs better for thicker materials (>6 mm) due to its rapid heat dissipation and deep penetration.
- Production Speed and Efficiency: The MIG method provides significantly higher production speeds, typically four to 10 times faster, reducing labor hours. The TIG method is slower, requiring manual filler rod feeding and inefficiency in long runs.
- Cost Considerations: The MIG method might have higher setup costs but offers lower per-meter weld costs due to speed. Despite requiring skilled operators, who increase labor costs, the TIG method is preferred for smaller, delicate runs to avoid wasting consumables.

Generally, it is recommended to use the TIG method for thin-gauge, esthetics-critical, or complex assemblies, and the MIG method for high-volume needs that emphasize speed over aesthetics.

#### 3.1.4. Cold Metal Transfer Welding of Mg Alloys

Cold Metal Transfer (CMT) welding effectively joins thin Mg sheets, repairs cast structures, and creates dissimilar joints, minimizing grain growth, thermal stress, and cracking with low-heat input [208]. According to [209], repairing surface defects in ZM6 cast alloy using Cold Metal Transfer (CMT) welding is significant due to its advantages over traditional TIG welding, including low heat input and minimal deformation. Cai et al. [208] systematically analyzed CMT welding parameters to eliminate unfused defects, examining variables like preheating temperature (310–450 °C), wire-feeding speed (5–7 m/min), welding speed (8–10 mm/s), stick-out length (12 mm), and shielding gas flow (20 L/min). They evaluated a defect of 11.5 mm depth with five repair paths, finding that continuous linear reciprocating paths provided better repair integrity. Optimal parameters enhanced melt spreading, arc stability, and overall weld quality in Mg alloys. According to [210], fabricating joints between AA 6061 and AZ31B is challenging due to metallurgical differences. Optimizing CMT welding parameters using parametric mathematical modeling led to strong joints. Optimized settings achieved 33 MPa TS and 95.8 HV WMH, with accurate forecasts under 1% error. Zhang et al. [211] used CMT to weld AZ31 alloy to galvanized steel. Optimal joint strength of 198 MPa was at 159 J/mm; excessive heat (181 J/mm) led to Zn volatilization, degrading interface properties. Ramaswamy et al. [212] studied CMT arc welding of 3 mm AA6061 and AZ31B alloys, finding that ER4043 produced defect-free joints but Mg-rich intermetallic reduced TS, highlighting the need to minimize them for better joint integrity. Chen et al. [213] found that traditional WE43 alloy manufacturing faced challenges with large structures. Cold Metal Transfer Wire Arc Additive Manufacturing (CMT-WAAM) offered flexibility and high efficiency. Different CMT modes improved microstructure and mechanical properties, with CMT-pulse achieving the highest YS of 149 MPa, TS of 222.9 MPa, and low porosity. Cao et al. [214] used CMT arc additive manufacturing to produce defect-free WE54 alloy samples, analyzing their microstructure and mechanical properties across different heat treatments. Findings revealed minimal composition changes, grain size increase, and ductile fracture in T4 samples. The T6 state showed superior tensile properties compared to castings. Zhang et al. [215] investigated the droplet transfer and stability in the swing arc additive manufacturing process of AZ91 alloy using CMT. They analyzed CMT parameters, established a relationship between burn phase current and the Vilarinho regularity index for short-circuit transfer IVSC, and developed a model for CMT optimization with a desirability function approach. Liu et al. [216] tested the welding of AZ31B alloy using CMT on 3 mm and 6mm thicknesses. Optimized parameters improved weld quality, with the 3 mm alloy reaching 83% and the 6mm 88.7% of base TS, indicating greater brittleness in thicker joints. Microhardness showed a W-shaped distribution, with the weld core being harder. Fatigue strength was 16.72 MPa, and fractures occurred at a 45° angle in the BM. Hou et al. [217] studied the mechanical behavior of Mg–1Sn–1Ca and Mg–2Sn–0.5Ca alloys under tensile loading. Resistance-spot-welding parameters influenced joint microstructure, nugget size, and TS. Optimized conditions (0.6 kN electrode force, 0.4 s welding time, 16 kA current) achieved a peak strength of 1.001 kN. Wei et al. [218] investigated CMT-WAAM for high-performance AZ31 alloy thin-walled components, enhanced by ultrasonic vibration. Results showed reduced surface roughness, refined equiaxed grains, and improved microstructure with specific phases. Grain diameter decreased from 38.3 μm to 22.5 μm, while mechanical properties improved, including a 9.9% and 21.1% increase in TS, and microhardness rose from 61.31 HV to 69.23 HV. Wang et al. [219] created a Mg/Al dissimilar lap joint using a modified Cold Metal Transfer process with Inconel 625 as filler metal. Microstructural analysis revealed six distinct areas at the weld interface near the Mg substrate. The tensile-shear load reached 1.28 kN, with failure occurring in the Mg_2_Al_3_-based area. Despite the presence of Mg_2_Al_3_ intermetallic, its detrimental impact on the joint’s strength was found to be limited. Wang et al. [220] reported that in wire-arc additive manufacturing (WAAM) of AZ31 alloy, modified Cold Metal Transfer (CMT) heat sources with optimized parameters were used to produce thin-wall components. The CMT process yielded a wide, shallow weld bead exhibiting good wettability and low heat input. Microstructural analysis revealed distinct layer characteristics, with fine columnar dendrite grains in deposited layers and coarse equiaxed grains with pores in the HAZ. The thin-wall component displayed anisotropic tensile properties, showcasing variations in YS and UTS. Strength enhancement requires minimizing the HAZ interlayers’ softening effects and addressing porosity. Bansal et al. [221] examined CMT welding of Al and Mg alloys, emphasizing optimal parameters for improved weld quality. Yang et al. [222] utilized Wire Arc Additive Manufacturing (WAAM) to fabricate Mg–9.54Gd–1.82Y–0.44Zr alloy thin-walled components. The microstructure features fine equiaxed α-Mg grains and various phases. High-temperature solution treatment enhanced elongation to 13.5%, and aging improved strength, achieving a YS of 235 MPa and UTS of 360 MPa, alongside good ductility. Liu et al. [223] optimized Wire Arc Additive Manufacturing of Al–Mg alloy walls using ER5556 wire, finding that lower current or higher speed resulted in finer grains, improved TS, and ductility. Shalomeev et al. [224] developed a scandium-containing filler metal, enhancing Mg–Zr–Nd welding for aircraft castings, improving mechanical properties, plasticity, heat resistance, and extending engine service life with economic benefits. Wu et al. [225] found that low electrode-positive/electrode-negative (EP/EN) ratios destabilize WAAM for Al–Zn–Mg–Cu, while optimal accuracy occurs between 4:16 and 13:7. Characteristics of the wire-arc DED process with different heat sources are shown in Table 4.

Summarizing, CMT welding of Mg alloys provides low-heat-input, stable welding for thin Mg sheets and Mg–Al joints, reducing spatter and brittle IMC formation. It enhances microstructure, yielding refined grains and higher TSs in Mg–Gd–Zn–Mn alloys. CMT optimizes dissimilar welding and utilizes advanced parameters. In additive manufacturing, it is vital for automotive applications focused on lightweight materials. Cold Metal Transfer (CMT) offers significantly lower heat input than traditional pulsed MIG when welding magnesium (Mg) alloys, resulting in reduced hot-cracking and element evaporation. Key parameter comparisons for AZ31/AZ91 alloys show that CMT operates at a lower welding current (80–100 A vs. 120–150 A) and arc voltage (12–14 V vs. 18–22 V), with slower travel and wire feed speeds. CMT’s minimal heat input prevents evaporation of critical elements, resulting in narrower, uniform weld beads, finer grain structure, and improved mechanical properties, including mitigated heat-affected zone (HAZ) softening and enhanced TS [50,208,227,228,229,230].

CMT joint efficiencies typically range from 70% to 90% of the BM’s UTS, and reduced thermal input positively impacts the ductility and tensile YS of the weld metal [50,208,229,231,232]. CMT addresses porosity and defect control, as magnesium is susceptible to hydrogen porosity [233,234,235]. Its pulsed, droplet-on-demand deposition minimizes the molten pool size and peak temperatures, which limits the evaporation of volatile elements and hydrogen solubility [50,232,236,237]. Strategies like using argon shielding gas, pre-weld laser cleaning, and optimized wire-feed rates can further reduce porosity [50,238,239]. Microhardness in CMT-welded Mg alloys shows a localized softening in the HAZ due to dissolution of strengthening precipitates [227,240,241], while the fusion zone may recover hardness through rapid solidification [235,242].

Debates on porosity sources focus on gas entrapment, moisture, and absorbed hydrogen as primary culprits [176,196,243,244]. Challenges include creating defect-free joints between Mg and Al due to brittle intermetallic compounds [212] and potential micro-cracking from high welding speeds [210,229].

#### 3.1.5. Utilization of Plasma During Joining of Mg Alloys

Plasma welding can relate to arc welding and laser welding, as well as plasma electrolytic oxidation and micro-plasma arc welding.

*Plasma for arc and laser welding.*

Belinin et al. [245] studied high-strength light alloy ML5, focusing on plasma surfacing effects on treated surfaces. Findings revealed a finer cast structure with reduced large intermetallics, while oxidized and anodized surfaces maintained quality, improving strength and ductility over cast metal. Nikulin et al. [246] reported that high-strength light alloy ML5 properties were enhanced through plasma surfacing with reverse polarity current. This method effectively repaired casting flaws and avoided issues like incomplete melting or cracks. Optimal parameters yielded a finer metal structure, improving hardness. The cathodic effect removed the oxidized layer without metal contamination. Plasma surfacing significantly improved mechanical properties, achieving 5–9 times higher ductility and 7–10% greater strength, relevant for both heat-treated and non-heat-treated conditions. According to [247], variable polarity plasma arc welding (VPPAW) effectively fuses Al and Mg alloys by eliminating oxide layers and forming keyholes through high energy density, allowing single-pass welding of medium-thick plates. Advancements in detection, modeling, and control, including X-ray systems and deep learning, enhance the understanding and versatility of VPPAW. According to [248], a low-energy pulsed laser can effectively refine the grains in welds of Mg alloys obtained using the TIG method.

Both plasma arc welding (PAW) and plasma–laser hybrid welding (PLHW) are two prominent methods for joining Mg alloys. PAW is known for deep penetration and less contamination sensitivity, while PLHW combines a laser and plasma arc, allowing for faster speeds and more refined weld profiles [249,250,251,252,253].

Wrought alloys, like AZ31 and ZK60, are preferred for such kinds of welding due to their reduced crack sensitivity [254,255] compared to cast alloys such as AM50 and AZ91, which are more prone to defects [249,256,257]. Welding parameters vary widely based on thickness and configuration and need optimization. PAW parameters include a current range of 80 A to 250 A, welding speeds between 100 mm/min and 500 mm/min, and shielding gases like pure He or a He–Ar mix at 15–25 L/min [258,259,260]. For PLHW, laser power ranges from 1.5 kW to 4.0 kW, arc currents of 50 A to 150 A, and travel speeds are between 1.0 m/min and 3.0 m/min, requiring a 1 mm to 3 mm inter-distance from laser to arc [251,253,261,262,263,264]. PAW is best for medium-to-thick materials (3.0 mm to 12.0 mm) [253,258,265], while PLHW excels in thin-to-medium gauges (1.5 mm to 6.0 mm) [251,253,266].

PAW is suited for low-volume, specialized applications, such as aerospace components [267,268,269,270], whereas PLHW supports high-volume automation, common in the automotive industry [50,271,272]. Hydrogen and gas entrapment contribute to porosity in Mg welding [108,271]. Preheating with plasma reduces porosity by degassing the surface [273,274], while hybrid synergy allows gas bubbles time to escape during cooling [256,275,276,277].

Joint efficiency for PAW typically ranges from 70% to 85%, while PLHW achieves 80% to 95% due to improved grain structure and heat input [45,50,108,276].

Key issues include evaporation during welding, altering the alloy’s composition, interference from the stable oxide film, and the complex dynamics of laser and arc integration, which require careful optimization [108,273,275].

*Micro-plasma arc welding of Mg alloys.*

Micro-plasma arc welding (M-PAW) is a precise, low-heat fusion method for Mg alloys, employing a constricted plasma jet with currents ranging widely. It is optimal for ultra-thin, heat-sensitive, or complex aerospace and electronics components [278,279].

Dwivedi and Bag [280] analyzed how input process parameters affect defect-free welds in M-PAW of thin materials, including Mg alloys. Key issues include overheating and grain growth. Heat input, a function of current, voltage, and speed, is identified as critical for achieving high-quality welds. M-PAW process can eventually be applied to similar joining of AZ31 alloy [281,282,283] and to dissimilar joints of Al–Mg [284,285], but further studies are needed.

Wrought alloys like Mg–Al–Zn systems (AZ31, AZ61) and RE alloys (e.g., WE43) exhibit better ability to M-PAW compared to cast variations [26,255,286,287]. Cast alloys such as AM60 and AZ91 are seldom welded via micro-plasma due to high porosity, preferring mechanical or solid-state joining methods instead [18,50]. Key welding parameters include welding current (2 A to 20 A, with a pilot arc of 3 A to 15 A), rapid WSs (5 mm/s to 15 mm/s), use of pure Ar shielding gas to protect molten Mg, and an arc voltage between 10 V and 25 V based on torch standoff and gas flow [279,288,289]. M-PAW excels in very thin sections (0.05 mm to 2.0 mm), particularly for sheets below 1.0 mm, where alternative methods like GTAW may fail [278,290]. M-PAW is typically used for small-batch production and custom engineering, yet its manual nature limits mass-market applications unless used in niche areas such as medical devices and aerospace sensor housings [18,50,278,279]. Challenges include Mg flammability [108,237,250], the potential for brittle intermetallic compounds when joining dissimilar metals [18,40], and the critical balance of heat input to achieve penetration without degrading material [50]. Porosity can be reduced by employing high WSs and strict pre-cleaning techniques [291]. Joint efficiency is impressive, achieving 60% to 90% of BM’s TS, contingent on managing the HAZ [50,292,293].

*Plasma arc weld-bonding.*

Plasma arc weld-bonding (PAWB) combines plasma arc fusion with structural adhesives to effectively join Mg alloys, focusing on lightweight applications in the automotive and aerospace fields. The technique addresses challenges of structural integrity and porosity commonly associated with conventional fusion welding [50,55,273]. Most commonly, PAWB is applied to wrought Mg–Al–Zn alloys such as AZ31B and AZ31, but it is also relevant for Mg-based composites and Al–Mg–Sc alloys [250,294,295]. Unlike traditional TIG welding, PAWB employs a concentrated, cylindrical plasma arc characterized by high energy density and variable polarity, alongside an adhesive layer [250,251]. Key welding parameters vary widely and require optimization. They include current settings from 60 A to 120 A based on thickness [296], variable polarity for oxide removal (electrode-positive and electrode-negative cycles) [247,258,297], a maintained arc length between 1 mm and 2 mm [247], and a WS ranging from 150 to 300 mm/min [50,247]. PAWB excels with medium-to-thin plates, typically joining sheet metals measuring 1.5 mm to 4.0 mm thick [247,298,299,300]. This technique is mainly utilized in low-to-medium-volume production for specialized applications, including automotive body panels and aerospace parts [50,250]. However, mainstream production processes often resort to solid-state methods like FSW for ultra-high-volume needs [50,301]. Challenges include porosity due to fusion welding’s low hydrogen solubility [291] and adhesive decomposition during the welding process, which releases gases potentially leading to porosity in the weld [247,302]. Successful mitigation strategies involve thorough pre-cleaning, effective joint design, and precise arc manipulation [291,302,303]. When optimized, PAWB joints achieve joint efficiencies of 70% to 85%, with enhanced failure loads, shear strength, fatigue life, and corrosion resistance [247,250,294,295].

*Plasma electrolytic oxidation.*

Siachen et al. [304] reported that plasma electrolytic oxidation (PEO), or micro-arc oxidation (MAO), produces oxide-ceramic coatings on metals like Mg, Al, and Ti. It involves electric discharges creating a plasma state, converting substrate material into a compound of substrate and oxygen, along with electrolyte components. The production of PEO has emerged as the most widely used protective technique for Mg alloys, such as Kernite or other coatings [305]. PEO process produces superior hard-ceramic coatings for Mg alloys, enhancing hardness, stability, wear, and corrosion resistance, broadening application possibilities [306]. The PEO system includes an electrolytic bath, a working electrode made of the electrically linked Mg alloy part, and a stainless-steel counter-electrode, which may serve as the wall of the cell housing the bath. The characteristics and composition of the layer are influenced by the properties of the bath and alloy, as well as by processing parameters, including reaction time and potential [306,307]. The voltages used can reach 500 V [308]. During PEO processes, discharges create plasma, forming an oxide layer through melting, movement, solidification, crystallization, and densification. Electric currents generate craters on the surface, with pores typically measuring a few microns in size [309]. Anodized coatings, particularly those created through plasma electrolytic oxidation, exhibit greater stability and provide improved corrosion resistance compared to chemical conversion coatings [310]. PEO layers are durable, wear-resistant, and corrosion-resistant, but they are fragile and electrically insulating, making them unsuitable for electric deposition processing. They are significant for orthopedic implants to reduce corrosion and serve as a pre-treatment, requiring application to protect substrates and improve coating adhesion [311,312].

PEO is effective on various Mg alloys, notably: AZ Series (e.g., AZ31, AZ80, AZ91D) for automotive, AM Series (e.g., AM50, AM60) for impact resistance, WE Series (e.g., WE43) for aerospace, and biodegradable alloys (e.g., Mg–Ca, ZM21) for orthopedic implants [313,314,315,316]. Key operating parameters vary widely and need optimization. They include voltage ranges of 200–700 V [317,318] and current densities of 5–50 A/dm^2^ [316,319,320,321], utilizing AC or bipolar pulsed waveforms to prevent overheating. Alkaline, non-toxic electrolytes are typically employed, with compositions like silicates, phosphates, and aluminates to enhance properties [317,322,323,324]. For welded components, such as those from friction stir welding, precise control over current density and oxidation times is necessary to accommodate microstructural variations [314,316,325]. PEO coatings are generally 5–150 μm thick and adaptable for high-throughput production, requiring minimal preparation [316,326,327,328]. Strategies for reducing porosity include doping the electrolyte with functional particles [329,330,331] and post-treatment sealing methods [316,327,331,332,333]. Joint efficiency remains stable during PEO treatment of welded assemblies, but variances in metal joints can affect coating performance [314,330,334]. Challenges in PEO involve navigating the thickness–porosity trade-off; thicker coatings offer better wear resistance yet develop micro-cracks. Additionally, concerns arise when applying PEO to galvanic couples, as standard operations may induce ignition or over-deposition, necessitating careful process adjustments [334,335,336,337].

Summarizing, plasma arc welding (PAW) and micro-plasma welding are advanced fusion techniques for Mg alloys, addressing limitations of conventional GTAW. PAW’s stable, constricted arc enhances penetration and narrows HAZs, ensuring high-quality joints. Micro-plasma welding operates at low currents for thin sheets, while keyhole welding serves thicker sections. The PAW principle utilizes a water-cooled nozzle for increased arc energy density, resulting in higher precision and reduced distortion. Such welding techniques can be applied in the aerospace and automotive sectors. Their challenges include Mg’s oxidation, porosity issues, and the need for surface preparation, with micro-plasma focusing on delicate components.

#### 3.1.6. Resistance Spot Welding

Resistance spot welding (RSW) of Mg alloys is often discussed in the literature. Welding Mg alloys with such a method is difficult because of their high reactivity and propensity to create hard, insulating oxide layers on the surface, potentially resulting in issues such as expulsion and cracking. Successful welding necessitates precise regulation of surface preparation and welding parameters (current, time, force) to oversee the stages of nugget formation, including incubation, growth, and stabilization, which happen more rapidly than in steel or Al alloys. The resulting welds may exhibit intricate microstructures and can be susceptible to failure [338]. The RSW process faces challenges influenced by factors [49,339] such as the oxide layer on Mg alloy surfaces, requiring thorough cleaning to avoid defects. Mg alloys necessitate unique welding settings, linking electrode design and conditions to microstructure and strength. Low melting temperatures and high thermal expansion lead to rapid solidification issues like cracks. Additionally, welding Mg to steel creates a fragile intermetallic. Common defects include expulsion, liquation cracking, and shrinkage porosity affecting joint integrity. For example, RSW of Mg alloys, such as AZ31, is difficult because of their low melting point, significant thermal expansion, and reactivity, which frequently result in defects such as cracking/porosity (AZ31) or expulsion (AZ91D) in conventional techniques. Nugget creation and microstructure of welds for Mg alloys strongly depend on the welding process [338]. The process of nugget formation consists of three phases: incubation, growth, and stabilization. Incubation is a brief period during which the nugget starts to develop because of melting. Incubation is followed by growth, a phase of swift nugget increase, succeeded by a declining growth rate. In the last phase, namely, stabilization, the nugget attains its ultimate size. The welding process affects microstructure, which can be intricate, occasionally containing elements such as fine dendrite regions and equiaxed dendrite regions. Quality of welds can be improved through various means [49,50], including surface preparation by cleaning to reduce oxide layers, optimizing welding parameters for nugget quality, and considering electrode features for better outcomes. Innovative techniques like resistance element welding (REW) enhance mechanical performance by creating dual-zone nuggets. Inverter DC welding offers better current regulation for uniform Joule heating compared to AC methods.

Zheng et al. [340] employed REW to join Al and Mg, mitigating brittle intermetallic issues. Welding current affects nugget formation and joint performance, with smaller nuggets improving ductility and load. However, excessive current increases brittleness due to melting, impacting performance, as evidenced by varying fracture modes. Wang et al. [341] conducted REW using AZ31B alloy as the upper plate and AA6061 alloy as the lower one, with rivets of 4, 6, 8, and 10 mm diameters. They examined the effects of welding current (WC) and welding time (WT) on the joint’s tensile shear load (TSL). The peak TSL reached approximately 6.99 kN with a 10 mm rivet at a WC of 35 kA, WT of 120 ms, and an electrode pressure of 4.8 kN. A reaction layer formed at the interface, consisting of Al_12_Mg_17_ grains aligned parallel to the interface. Abankar et al. [342] reviewed recent REW research focused on steel and non-ferrous (including Mg) alloy joining, highlighting microstructural and mechanical properties, innovations, process parameters’ influence on joint quality, as well as simulation studies and comparisons with conventional joining techniques.

Generally, RSW faces challenges due to material characteristics, forming undesirable structures. Techniques like electromagnetic stirring improve strength, while REW is effective for joining materials, reducing weight for automotive and aerospace applications [342,343].

RSW of Mg alloys is challenged by high thermal conductivity, rapid oxidation, and chemical reactivity, achieving joint efficiencies of 70–85% [344,345,346,347,348]. However, the typical joint efficiency is lower and ranges from 50% to 72% [349], heavily influenced by optimal nugget diameter linked to welding current, pressure, and weld time [347,350,351,352]. Given fusion limitations, industries adopt solid-state methods like refill Friction Stir Spot Welding (RFSSW) for enhanced mechanical strength and nearly 100% joint efficiency [353,354,355]. Defect management is crucial due to liquid magnesium’s high vapor pressure and cooling rates [18,50,356,357,358]. Porosity results from gas entrapment and can be reduced through shorter welding times, cooling cycle optimization, and solid-state techniques [18,110,346,359,360]. Liquation and solidification cracking occur in alloys like Mg–Zn–Zr, mitigated by precise pressure control and multi-stage current cycles [344,346,361,362,363]. Oxide inclusions and electrode wear create inconsistent contact resistance, and thus cleaning sheets before welding helps reduce these issues [364,365]. The NZ shows high microhardness (70–85 HV) [347,366,367], while the HAZ experiences grain coarsening and the lowest microhardness, marking a common failure zone [50,344,368,369]. The BM retains its original hardness, contrasting with the HAZ’s properties [338].

#### 3.1.7. Laser Beam Welding

Laser welding is an efficient method for joining dissimilar metals, offering controllable heat input and low costs [370]. It enhances production efficiency in automobile manufacturing and minimizes manual defects. Use of laser beam welding (LBW) of Mg alloys plays a significant role in reducing weight in automotive/aerospace applications, delivering high-quality results but encountering issues such as porosity, cracking, spattering, and debris, as well as coarsening of grains and decreased mechanical characteristics in welds. Thus, controlling intricate heat transfer and material movement in the molten pool is needed [26,50]. Researchers tackle these challenges by employing hybrid methods (laser–arc), beam oscillation, rare-earth element additions, and optimizing parameters (power, speed) to create defect-free welds with enhanced strength, concentrating on microstructural management for superior mechanical performance [26]. LBW has interesting characteristics and potential for its improvement [26,50]. Defect mitigation in laser–arc hybrid welding reduces undercuts and pores by optimizing heat input and weld pool dynamics [371]. Beam oscillation is key for microstructure management, converting columnar grains into smaller, equiaxed grains that enhance strength [372]. Optimal laser parameters are essential for fine grains and tensile properties. Rare earth elements improve microstructures and reduce defects [26]. In dissimilar Mg to steel joints, addressing IMCs is crucial, with a focus on IMC regulation and appropriate filler metals to enhance strength. Joint strengths over 6000 N relate to weld interface width and microstructural uniformity, with settings adjustments improving the process and achieving desired microstructures. Low oscillation frequency enhances mechanical properties, while ultrasonic welding reduces porosity and refines grain size to improve joint quality in AZ31B and AZ61 alloys through proper parameter adjustments [373,374].

*Mg alloy/Mg alloy*

Heat input is crucial in laser welding of Mg alloys, as it must be controlled to prevent welding defects [55]. Shi et al. [375] explored welding parameters’ effects on joint performance, identifying peak power as the most significant factor, noting a maximum TS of 142.5 MPa. The likelihood of thermal cracks diminished with increased pulse time, showing zigzag cracks at 5 ms, fewer cracks at 6 ms, and no cracks at 7 ms. Kumar et al. [376] reported that for most Mg alloys (AZ61, AZ31, AZ91, AZ80), an increase in welding current (90 A to 150 A to 210 A) leads to increased depth of penetration, indicating a strong relationship. Additionally, higher laser welding voltage and welding speed contribute to deeper penetration. Optimal parameters for the highest penetration in AZ61 and AZ80 occur at 60 cm/min speed, 26 V voltage, and 210 A current. According to [376], laser welding (Nd: YAG, CO_2_) can be successively applied for various Mg alloys, including AZ61, AZ31, AZ91, and AZ80.

Overall, the studies concluded that heat input influences joint formation and internal structure changes, suggesting that increasing heat input and adding Al enhances grain refinement, extends liquid metal flow time in the weld pool, strengthens joints, and minimizes hot cracks and pits.

Controversies and challenges in the welding of similar Mg alloys include issues such as porosity and blowholes due to low boiling points and high vapor pressure, leading to vapor trapped during rapid solidification. This results in weld pool instability and spatter. Hot cracking occurs from rapid thermal cycles in LBW [55,286,377,378].

*Mg alloy/Al alloy*

Cui et al. [379] joined AZ91 and AA6061 alloys using double-molten-pool laser welding. Two laser beams created independent molten pools with a Ni interlayer, optimizing heat input for Mg and Al alloys. The Ni interlayer prevented the formation of hard Mg–Al intermetallic. The Mg weld microstructure contained Mg_2_Ni, α-Mg, and dispersed Mg3AlNi2, while the Al weld featured AlNi_3_, Al_3_Ni_2_, and α-Al. Optimal welding parameters were 1.0 m/min at 1300 W for Mg and 0.6 m/min at 1200 W for Al, resulting in a TS of 83.58 MPa. Cui et al. [380] joined AZ91 and AA6061 alloys using swing laser welding. The transverse swing of the laser beam stirred the molten pool, optimizing the Mg–Al IMCs and forming a mechanical interlocking structure that mitigates their adverse effects. Microstructural analysis revealed a wave-shaped interlocking structure comprising (α-Mg + Al_12_Mg_17_) eutectic. The laser power greatly influenced joint performance, with 900 W yielding a shear strength of 40.72 MPa and brittle fracture in the interlocking zone. Numerical simulations indicated a uniform temperature distribution due to the swinging heat source, enhancing joint quality.

For Mg/Al alloys, managing brittle intermetallic compounds (IMCs) like γ-Mg_17_Al_12_ is critical, as they can significantly weaken joints. The varying evaporation rates of Mg and Al create composition segregation [378,381,382,383,384].

*Mg alloys/steel*

Laser welding facilitates the joining of Mg alloys and steel but faces challenges due to significant differences in their properties, leading to poor weldability [55]. Interlayers like Cu, Sn, V, Ag, and Cu–Zn enhance joint performance and simplify welding [40]. Direct welding often results in defects such as unmelted areas and cracks, resolved by employing interlayers [55]. According to [40], solid solutions and intermetallic phases facilitate Fe and Mg bonding through interlayers, but brittle IMCs at Mg–steel interfaces can compromise mechanical properties. To address this, interlayer thickness and Fe–Mg intermixing should be minimized. The impact of alloying element diffusion during laser welding is significant, along with Cu, Zn, and Ni interlayers. Research should explore eutectic-forming interlayers like Ag and Al, while combining fusion and solid-state methods may enhance weld quality. Zhou et al. [385] conducted tests on laser fusion welding with Al foil for DP590 steel and AZ31B alloy, showing that the addition of Al foil reduces defects like pores and cracks, enhances bonding strength, and regulates heat transfer. The resulting dual molten pools form beneficial Al–Fe phases, improving metallurgical connections between them.

Generally, choosing the right interlayer material improves the weldability of Mg/steel combinations, with heat input being crucial for joint performance control. In Mg/steel welding, metallurgical immiscibility results in poor fusion, and the low absorptivity of Mg requires high laser energy, often leading to blowholes [40,386].

*Mg alloy/Ti alloy*

Mg alloys and Ti alloys offer good corrosion resistance and are often combined, with laser welding being a vital method for their connection [55]. Al and Cu serve as interlayers to enhance joint properties and microstructure during welding [55]. Zhu et al. [387] explored the use of Cu as an interlayer, finding that it not only improves mechanical properties but also affects interface diffusion and reactions. The Cu interlayer enhances microstructure at the weld–Ti interface and strengthens the bonding of Ti/Mg joints. As the thickness of the Cu interlayer increases, the Ti_2_Cu reaction layer becomes continuous, shifting the fracture location from the interface reaction layer to the weld zone; this leads to a TS of 121 MPa when the interlayer reaches 30 µm. However, brittleness of IMCs and cracking during the welding process remain challenges, limiting the effective combination of Mg alloys with other metals in fields like automotive manufacturing and defense. Zhang et al. [388] studied TC4 alloy surfaces coated with TiB_2_ ceramic particles and bonded with AZ31B alloy. They found that TiB_2_ incorporation led to new ceramic phases (Ti_2_B_5_, AlB_12_, TiB_55_), promoting intergranular nucleation and reducing average grain size from 31.45 μm to 18.37 μm. Nano-sized TiB_2_ particles hindered crystal nucleus growth. The joint’s interface revealed semi-coherent relationships between phases, enhancing TS from 29 MPa to 50.5 MPa and increasing hardness by 79.75%. Apart from grain refinement, mechanisms of strengthening included load-bearing reinforcement and CTE mismatch strengthening. According to [389], essential requirements for a successful joint between Mg and Ti alloys include the use of filler metals or intermediate elements like Al or Zn to facilitate metallurgical bonding by forming IMCs (e.g., Al–Mg, Al–Ti), which enhance bond strength and reduce brittleness. Optimal laser power is critical for effective melting without overheating or causing defects; a stable keyhole is necessary for deep penetration welding. Controlling laser beam offset and oscillation aids in managing heat input and improving mixing, addressing the melting point disparity between Mg and Ti. Interlayers such as Cu–Ni or Ni–Cu promote strong interfacial bonding while minimizing brittle intermetallic formation. Shielding gases like argon are vital to prevent oxidation, while proper surface cleaning ensures a strong joint. Post-weld heat treatment (PWHT) relieves stress and enhances mechanical properties. These factors collectively ensure a uniform weld bead with minimal defects.

Challenges in Mg/Ti welding include a large melting point difference, making it difficult to weld without specialized techniques [45,165]. Moreover, Mg and Ti do not form solid solutions, complicating bonding [165], while debate exists over using various interlayers to create effective metallurgical bonds without brittleness [165,389].

Summarizing, laser welding can be successfully applied to Mg and various Mg alloys. Laser welding Mg/Al faces challenges like brittle intermetallics. Solutions include lap joints with Mg on top, oscillating laser beams that stir molten pools, and interlayers/fillers (Ni, Zn, Ti) to inhibit brittle layers. The optimal result is a wavelike interface achieving TSs around 80 MPa. For LBW Mg/steel, challenges involve immiscibility and porosity. Solutions include laser welding–brazing (LWB) with Zn-coated steel, interlayer coatings, and dual-beam lasers to enhance bonding. This can produce high-strength joints, often failing in the Mg BM. LBW Mg/Ti faces low solubility and melting point differences. Solutions utilize laser keyhole welding and Al/Zn fillers, resulting in joints with high TSs up to 266 MPa. LBW of Mg alloys achieves narrow HAZs and minimal distortion but faces challenges like low joint efficiency, porosity, and grain coarsening [26,176,255,390,391]. Optimizing parameters, such as utilizing high WSs and reduced heat input, is critical for defect-free joints. Typical joint efficiency ranges from 60% to 85% of the BM’s UTS [390,392], with adjustments in laser power (1.5 kW–2.0 kW) promoting finer microstructures that enhance TS [45,360,393]. Failures often occur at the WZ and HAZ boundary due to localized softening [394]. Porosity is primarily caused by keyhole instability, gas entrapment, and Mg evaporation [360,395,396,397]. To minimize defects, higher welding speeds narrow the weld pool and allow gas escape, while laser beam oscillation agitates the pool to suppress porosity. Advanced techniques like ultrasonic-assisted welding can reduce porosity below 1% [26,390,395,398,399]. Hardness distribution reveals that the WZ has the highest microhardness [400,401], while the HAZ shows reduced hardness due to grain coarsening [394,402], with the BM maintaining uniform hardness [402]. Mg’s unique physical properties make its LBW highly controversial in mass production due to challenges including porosity due to Mg’s solidification range [271,403], hot cracking from high thermal stresses [378,404], evaporative loss of alloying elements [55,403,405,406], exacerbation of galvanic corrosion with dissimilar metals [40,407,408,409], and ongoing debate regarding filler materials versus rare-earth modifications against fusion welding [26]. Solid-state welding methods are often proposed as alternatives to mitigate defects in Mg–Al joints [381].

#### 3.1.8. Electron Beam Welding of Mg Alloys

Electron beam welding (EBW), ideal for thick plates, offers controllable heat input and simple operation. The vacuum environment prevents oxidation of active metals like Mg and Al, enhancing joint performance. Its rapid heat input and shorter high-temperature exposure create a uniform interface grain, improving joint strength [55]. Weldability of Mg with the other structural materials, as regards EBW, is shown in Table 5, based on data from [410]. EBW of Mg alloys to Al alloys is challenging, as Mg alloys have poor weldability to Al alloys due to their physical and chemical features [411].

*Mg alloy/Mg alloy*

EBW is effective for Mg alloys due to its lack of restrictions on workpiece size and shape. It offers advantages such as limited deformation, small heat-affected zones, and excellent joint integrity, crucial for the automotive industry [55]. Chen et al. [412] examined the effect of heat input on the microstructure and mechanical properties of EBWed AZ31 alloy. Tensile tests indicated that the welded joints had better strength than the BM, but ductility decreased significantly. Strength and ductility increased initially and then decreased with rising heat input, peaking at 180 J·mm^–1^ and 216 J·mm^–1^, respectively. Microstructural changes, such as increased size and quantity of Mg_17_Al_12_ precipitates and weld shape alterations, influenced these properties. Fracture morphology differed notably between the FZ and BM, with the latter displaying intergranular fracture patterns. Lei et al. [413] studied the microstructure and properties of VEB-welded WE43 alloy. Using parameters of 150 kV acceleration voltage, 120 mA current, and 35 mm/s speed, the weld mainly consists of α-Mg and β-Mg_24_Y_5_ rare earth phase. The weld exhibits higher hardness (27% more), YS (17% increase), TS (14% increase), and elongation (41% increase) than the BM, indicating improved performance. The mechanical properties of these joints are mainly affected by welding current, speed, and accelerating voltage. EBW reduces welding defects and promotes uniform grain structures, enhancing joint performance effectively [55]. Yi et al. [414] studied EBW of dual-phase LAZ832-0.5Y alloy, focusing on joint microstructure and mechanical properties. They found that high thermal efficiency allowed rapid welding and cooling, with effective joints formed under specific conditions (60 kV, 750 mm distance, 500 mm/min speed, 1080 mA focus current, and 7.4 mA beam current). Fractures occurred in BM, maintaining good ductility and mechanical consistency. Microhardness increased in both FZs and HAZs, with fine grain and precipitation strengthening observed, particularly from α-Mg, β-Li, and Mg3(Al, Zn). Sun et al. [415] optimized the joining of Mg–12Li–3Al–2Zn–1Si–1Y alloy plates via EBW by controlling heat input, leading to fewer welding defects and improved surface quality under specific parameters (60 kV, 1180 mA, 133.7–164.6 J/mm). The solidification process results in the precipitation and spheroidization of Mg3(Al, Zn) phases within the β-Li matrix. Increased heat input shifts fracture locations from FZ to BM and then to HAZ, enhancing plasticity but risking grain coarsening at excessive heat levels. Optimal heat input (123.4–164.6 J/mm) yields TS around 280 MPa and joint efficiency > 95%, bolstered by phase strengthening and grain refinement.

*Mg alloy/steel*

According to [55], in EBW of steel and Mg alloy, an interlayer enhances weldability and mechanical properties. Particularly, the Al–Zn coating had an important effect on Q235 steel and AZ31 Mg alloy joints via vacuum electron beam welding. Without an Al coating, an effective interface was unattainable. The Al–Zn coating facilitated Zn evaporation and diffusion, enhancing Mg/steel interface wettability and weldability. An Al_5_Fe_2_ IMC layer of approximately 100 nm formed at the interface, achieving a shear strength of 110 MPa. Fractures occurred at the interlayer/AZ31 interface, highlighting challenges in welding steel to Mg alloy.

Summarizing, EBW of Mg alloys offers numerous advantages, including high-quality, narrow welds with minimal heat-affected zones, making it ideal for thick or complex components. The vacuum environment prevents oxidation, enhancing the weldability of Mg–Al alloys (e.g., AZ31, AZ61, AZ91) and rare-earth alloys (e.g., WE43). EBW achieves a high depth-to-width ratio and refines microstructures, producing stronger welds. For Mg–Al–Zn alloys, increased Al content improves weldability; particularly AZ91 features fine equiaxed grains. WE43 displays fine α-Mg and β-Mg_24_Y_5_ phases, with increased hardness. However, challenges include porosity and keyhole instability, mitigated through optimized beam parameters. Cochleoid scanning paths effectively reduce porosity [416], while the low surface tension of molten Mg may cause spattering issues. EBW employs a high-energy electron beam in a vacuum, achieving joint efficiency of 84–95% [415,417], excellent porosity reduction by eliminating gas-induced issues [42,418], and a narrow fusion zone with hardness similar to the BM, though high heat can cause HAZ grain coarsening [415,418]. Hardness decreases in the HAZ while the fusion zone (FZ) shows variable behavior [50]. Wrought alloys like AZ31 experience significant grain refinement, often leading to hardening [414,419], while cast alloys like AZ91 may have a slight hardness drop due to a fine-grained structure [55]. The HAZ exhibits softening from thermal cycling, making it the weakest point, prone to premature failure [55]. Increasing Al in the AZ series boosts hardness but worsens softening in the HAZ [55]. To improve hardness uniformity, post-welding heat treatments are used to enhance microstructural homogeneity across the weld [50].

#### 3.1.9. Filler Materials

Welding Mg alloys demands meticulous choice of filler metal to reduce weld cracking and manage brittle intermetallic compounds (IMCs). Choosing the appropriate filler—mainly AZ61A or AZ92A for similar alloys—guarantees outstanding crack resistance, robust mechanical properties, and suitable melting ranges [382,420,421].

When welding similar Mg alloys, the filler metal generally matches the BM composition, ensuring comparable corrosion resistance and strength [146,422,423]. Various fillers applicable for welding of similar Mg alloys are shown in Table 6.

**Table 6:** Fillers applicable for welding similar Mg alloys.

Base Metal	Recommended AWS Filler	Key Characteristics	Preferred Process	Refs.
**Mg–Al–Zn Alloys** (AZ10A, AZ31B, AZ31C, AZ61A, AZ80A)	ER AZ61A	Resists crack sensitivity in aluminum-containing wrought alloys.	GTAW/GMAW	[50,167,420,421]
**Mg–Al–Zn/Mg–Al Alloys** (AZ61A, AZ63A, AZ91C)	ER AZ92A	Lowest crack sensitivity for cast magnesium alloys.		
**Mg–Zn–Zr Alloys** (ZK21A, ZK60A)	ER AZ61A or EZ33A	Provides high strength; matching fillers are usually applied based on specific temperature.		
**Mg–RE/Mg–Y Alloys** (WE43, WE54)	WE43 (Specialized)	RE (Y) is used to prevent hot cracking in high-heat creep-resistant alloys.	GTAW/Laser	[42]

Joining Mg alloys to other Mg alloys of different series, or to different metals (e.g., Mg to Al), is highly challenging. Dissimilar combinations risk forming fragile, brittle intermetallic zones. Using specialized fillers and interlayers is crucial to control these microstructures [383,424,425,426]. Various fillers applicable for dissimilar welding of Mg alloys are shown in Table 7.

**Table 7:** Various fillers applicable for dissimilar welding of Mg alloys.

Dissimilar Materials	Recommended Filler/Interlayer Approach	Comments	Preferred Process	Refs.
**Mg Alloy to Mg Alloy** **(e.g., AZ31 to ZK60)**	AZ92A or AZ61A	Recommended fillers should match the lower-melting-point base alloy to allow the weld to freeze properly. Prevents hot-cracking and maintains a homogeneous microstructure.	FSW or LBW	[224,354,420,421,427]
**Mg Alloy to Al Alloy** **(e.g., AZ31B to 6061)**	Zn interlayer, or ER4043/ER5356; Al–Si alloy, Zn–Al alloy, or Al–Mg alloy.	Zn or Al interlayers act as a barrier to prevent the direct reaction between Mg and Al, drastically reducing the formation of brittle phases. Elevates pool fluidity, refines grains, and suppresses brittle Mg–Al phases (e.g., γ-Mg_17_Al_12_).	Laser or Hybrid welding	[48,382,383]
**Mg Alloy to Steel** **(e.g., AZ31 to Galvanized Steel)**	Zn or Al-rich filler metals; Ag, Cu, Sn, Zn, or Zn–Al–Ce.	Zn and Al react to form a thin, ductile reaction layer rather than thick, brittle Mg–Fe IMCs. Interlayers promote interfacial bonding, suppress brittle intermetallics, and avoid pure Mg/Fe immiscibility.	CMT, Laser–arc Hybrid/TIG Brazing	[40,428,429,430,431]
**Mg Alloy to Ti**	Al-based fillers (e.g., Al and its alloys).	Enables low-temperature joining without excessive formation of brittle Mg–Ti intermetallic.	Laser Brazing	[429,432]

For specific aerospace or industrial applications, recent metallurgical developments have introduced new filler formulas:

- RE Fillers: Experimental fillers using compositions like Mg–3Nd–1.5Gd–0.5Zr–0.3Zn enhance TS and reduce porosity in AZ-series joints [57].
- Sc Additions: Mg–Zr–Nd fillers modified with scandium promote fine-grained structures and improved long-term strength at higher temperatures [224].

### 3.2. Solid-State Welding

Solid-state welding includes ultrasonic welding, diffusion welding, explosive welding, friction stir welding, and friction stir processing. Solid-state welding for Mg alloys offers advantages like defect prevention, minimal heat-affected zones, and suitability for dissimilar metal joining. Key parameters include optimized rotation speed, tool design, pressure management, and surface cleaning, making it ideal for aerospace, automotive, and railway applications [381].

#### 3.2.1. Ultrasonic Welding of Mg Alloys

There are several excellent reviews related to ultrasonic welding (USW) of Mg alloys. Zhang et al. [433] highlighted Al and Mg as important light metals in automotive manufacturing, with ultrasonic metal welding (USW) enhancing bonding quality and joint performance through optimized parameters and numerical simulations. Rubino et al. [434] noted that ultrasonic welding quality in Mg alloys relies on processing parameters. Energy affects bonding and fatigue resistance; further research on thermomechanical models is needed. Zhao et al. [435] studied ultrasonic welding of AZ31B alloy and pure Cu, finding a maximum joint strength of 3798 N due to the Mg_2_Cu diffusion layer and significant deformation at edges.

*Mg alloy/Mg alloy*

USW of Mg alloys, particularly AZ31 [436], AZ31B [437], and Mg_96_Zn_2_Y_2_ [438], has seen limited research. Selvaraj et al. [439] noted that longer weld times and pressures improve TS, and interlayers further enhance mechanical properties and fatigue life. Together, these findings emphasize the importance of interlayers and process conditions in ultrasonic welding of Mg alloys. Li et al. [196] proposed a novel approach combining ultrasonic vibration with hot rolling, which significantly enhances fusion welding in die-cast magnesium alloys, addressing severe porosity issues. This method reduced pore content by 85.51% and improved ultimate TS by 244.46% and elongation by 71.70%. The authors analyzed the porosity mechanisms and concluded that N_2_ gas entrapment during die-casting was a primary cause. The cavitation effect during ultrasonic vibration helped refine microstructures and eliminate larger pores. The authors obtained superior weld quality and mechanical properties in USWed die-cast magnesium alloys. Xu et al. [440] reported that ultrasonic vibrations positively affected AZ31B alloy during LBW; 50% assistance eliminated porosity, increased TS to 259.6 MPa, and enhanced elongation to 11.1%, while 90% assistance worsened weld quality and properties.

*Mg alloy/steel*

Welding dissimilar materials such as Mg alloys with steel and Al presents challenges due to differing melting points and reactions at the interface, notably IMC formation that adversely affects joint properties [104,383]. USW utilizing interlayers like Sn and Zn [441] can be applied to improve bonding. Wang et al. [442] used an ultrasonic-laser welding–brazing method to enhance the welding performance of AZ31B alloy on 304 stainless steel. The optimal conditions were at 2000 W laser power and a 45° wetting angle, yielding continuous joints. Ni-plating improved wetting and introduced new phases (AlNi, Mg_2_Ni) in the FZ. The hardness of the FZ was intermediate, with a maximum fracture shear strength of 222 N/mm.

*Mg alloy/Al alloy*

Su et al. [443] used USW to join AZ31B and AA6061 alloys to enhance joint strength. At 1540 J welding energy, evidence of melting appeared, with IMCs forming a 17 μm-thick reaction layer. The IMCs consisted mainly of γ-Mg_17_Al_12_ and β-Mg_2_Al_3_ phases, with hardnesses of 3.4 GPa and 6.5 GPa, respectively. Increasing energy led to rapid β-phase growth, decreased joint performance, and cleavage fracture with secondary cracks. Chen et al. [381] reported that optimizing welding parameters such as current, time, force, tool profile, and travel speed is essential for improving aluminum/Mg dissimilar alloy welding performance. Utilizing an intermediate layer can prevent the formation of Al–Mg IMCs, enabling successful alloying. Hybrid welding methods leverage various techniques, particularly ultrasonic waves, enhancing the welding of dissimilar alloys.

*Mg alloy/Ti alloy*

As reported in [434], USW can be used to join AZ31 and Ti6Al4V alloys, successfully promoting metallurgical reactions by adjusting process parameters. Al emerged from the Mg alloy’s liquid phase, aiding the process and preventing IMC formation due to lower heat input. No microstructural changes occurred on the magnesium side with welding times between 300 and 600 ms, while grain size refined to 3–4 μm at interfaces for longer times. Joint strength, peaking at 3762 N, depended on clamping force, amplitude, and vibration time, with defects arising at 800 ms.

*Mg alloy/Cu alloy*

Abbas et al. [444] analyzed ultrasonically welded Cu–Mg sheets with Ni and Zn interlayers, exploring thermal mechanisms, interface temperatures, and their impact on mechanical properties, demonstrating Ni’s superior performance over Zn in enhancing joint strength and fracture resistance. Peng et al. [445] noted the challenge of USWed Al/Mg/Al three-layer plates in new energy vehicles. They developed and validated a thermal-force coupling analysis model. They found that tensile shear strength increased initially with welding energy but later decreased, with joint failure occurring at the interface and a maximum tensile load of 704 N. The bonding relied on mechanical interlocking without metallurgical reaction, and the maximum welding temperature can reach 550 °C, as determined by the numerical model. Zhao et al. [435] examined the USW of AZ31B alloy and pure Cu. They observed interface-type fracture as the joint’s failure mode, achieving a maximum joint strength of 3798 N. The bond formed via an Mg_2_Cu diffusion layer, growing towards both metals during welding. The interface reached up to 550 °C, with notable plastic deformation occurring after 500 ms, particularly at the solder joint’s edge due to acoustic softening and high temperature.

*Hybrid USW of Mg alloys*

Recent advancements in ultrasonic energy application for joining Mg alloys have shifted focus from traditional USW to integrating ultrasonic waves with other welding methods. Ultrasonic-assisted friction stir welding (UAFSW) and Ultrasonic-Assisted Friction Stir Spot Welding (UAFSSW) are interesting alternatives to RSW. These hybrid techniques reduce energy consumption and enhance joint quality. Conventional FSW addresses issues like cracks and porosity effectively [434]. Ultrasonic assistance promotes beneficial phase formations, enhances material flow, and improves weld integrity [434]. Further research is needed in conjunction with other welding methods.

*Numerical modeling of USW*

USW is complex due to rapid temperature changes, localized plastic strains, and varying contact conditions affecting real-time data acquisition. Finite element simulations [434] focus on temperature evolution and incorporate heat from friction and deformation. Zhao et al. [435] developed the finite element analysis model of the Mg–Cu USW process. Huang et al. [446] used a multiscale simulation approach to optimize ultrasonic welding conditions for AZ31 alloy and dual-phase steel DP590. A mesoscale model linked the friction coefficient to surface roughness, while a macroscopic model showed that increased roughness enhanced heat generation. Using various finishing methods, joint strength improved 10–25% due to better interlocking and heat generation.

In summary, the USW of Mg alloys prevents fusion defects by creating solid-state bonds via localized heat and pressure. Joint quality depends on processing parameters, with temperature influenced by energy and surface conditions, affecting shear resistance and fatigue life, simulated with finite element models. Interestingly, USW is primarily a solid-state process, with conflicting studies on whether melting occurs at the interface, where some suggest bonding is due to solid-state friction and deformation, while others indicate localized melting in Mg alloy [433,447,448]. Increased welding energy may refine grain size and hardness through dynamic recrystallization, but excess energy can cause grain coarsening and cracking [447,449]. In dissimilar welds, thin IMC layers may enhance integrity, but excessive growth leads to brittleness and failure [381,382,450]. The use of ultrasonic vibration (UV) significantly improves the welding efficiency of Mg alloys by enhancing mechanical properties, reducing porosity, and refining hardness distribution. The introduction of ultrasonic waves leads to acoustic streaming and cavitation, which stir the molten pool, refine grain structures, and ensure uniform hardness in welded joints [42,153,154,434,451,452]. This contributes to increased UTS and elongation, often raising UTS by 5 to 20% compared to conventional methods [50,153], while maintaining 95+% of BM’s strength [451]. Ultrasound effectively reduces weld porosity from over 4.3% to less than 0.9% through micro-bubble coalescence and escape [153,434,453]. It also minimizes defects like hot cracking and improves the dispersion of intermetallic compounds (IMCs) [451,454]. Additionally, ultrasonic vibration promotes a fine, equiaxed grain structure in the fusion zone, leading to more uniform hardness profiles (e.g., ~115–200 HV) [50,55,440,455,456].

#### 3.2.2. Diffusion Welding of Mg Alloys

Diffusion welding bonds materials by atom diffusion under specific temperature and pressure. Performance initially improves with longer time and higher temperature, but excessive conditions can form IMCs, degrading joint strength [55].

*Mg alloy/Mg alloy*

The diffusion welding of Mg alloys is affected by welding temperature, holding time, and welding pressure, which, when optimized, enhance joint performance. Adding Cu and Al can improve interface reactions, fostering IMCs that boost mechanical properties and joint quality [55]. Falchenko et al. [457] studied vacuum diffusion welding of MA2-1 magnesium alloy, revealing that unsupported welding without an interlayer at 400 °C fails. Successful joint production occurs with a 250 μm zinc interlayer at 320 °C and 10 MPa for 30 min, yielding specific grain structures. Peng et al. [458] created a double hollow structural component of Mg–8.3Gd–2.9Y–0.8Zn–0.2Zr alloy using superplastic forming and reaction–diffusion bonding. Maximum tensile elongation reached 1276.3% at 400 °C. Bonding quality varied with temperature, affecting shear strength, which peaked at 152 MPa at 460 °C.

*Mg alloy/Al alloy*

Yang et al. [459] bonded AZ31 and AA3003 alloys via diffusion welding using Ag–Cu–Zn under argon, forming a complete metallurgical bond at 420 °C for 120 min. Diffusion layers were developed, with Al diffusing further than Mg. The joint’s hardness reached 194 HV near Al and 111 HV near Mg. The maximum tensile shear strength was 71 MPa. Compound layers included Ag_3_Mg and CuMg_2_, enhancing interface hardness. Chen et al. [460] performed diffusion bonding of AZ31 and AA5083 alloys with a Zn–2Bi interlayer at 335 °C and 4 MPa. The joint’s microstructure comprised Al(Zn) solid solution, Al–Zn eutectoid, Zn–2Bi remnants, and MgZn_2_ IMCs. Maximum hardness reached 255 HV; peak shear strength was 20 MPa. Petrushynets et al. [461] investigated how temperature and the chemical composition of intermediate layers affect the diffusion-welded joint structure of AMg2 and MA2-1 alloys. An 80 μm diffusion zone, with a microhardness of 2160 MPa, forms at 400 °C, leading to cracking. A 30 μm nickel or titanium foil barrier reduces diffusion and prevents brittle intermetallic phases. Application of Ti foil resulted in the strongest joints (σUTS = 138–142 MPa).

*Mg alloy/steel*

Diffusion welding is more effective than fusion welding for joining Mg alloy and steel, optimizing joint performance by adjusting welding temperature and holding time. Using interlayers like Cu and Ni can enhance welding conditions and quality [55]. Shirzadi et al. [462] studied the challenges of bonding Al–Mg alloys to steels, specifically Al-5056/Al-5A06 with stainless steels 1Cr18Ni9Ti and 316. They tested various interlayers, including Ga, Ti, Cu, and Al foils. They developed and assessed multiple-stage processes using up to three interlayers. The highest bond strength achieved was 226 MPa, surpassing the YS of the alloy. A bonding cycle was created, producing joints with strengths of around 90 MPa. The difficulties in brazing Al alloys with high Mg content. Al Badour et al. [463] employed friction stir diffusion bonding (FSDB) to create a solid-state lap joint between ZK 60-T5 alloy and steel ASTM A 516-70. Utilizing an FSW tool without pin penetration, heat from friction and forging initiated diffusion. The study revealed tensile failure in Mg within the NZ at optimum conditions, indicating reduced strength compared to the unprocessed alloy.

*Mg alloy/Ti alloy*

Diffusion welding is a solid-state method suitable for welding difficult metal combinations, such as Mg and Ti alloys. The addition of an interlayer enhances welding efficiency and reduces diffusion challenges. Typically, Al-containing materials serve as interlayers in these processes to optimize joint properties. Studies demonstrate that welding temperature significantly affects joint phase composition and distribution, impacting joint performance.

Cooke [464] studied diffusion bonding of Ti6Al4V and AZ31 alloys, exploring microstructural evolution and intermetallic. They obtained successful bonding with or without an interlayer, featuring TiAl_3_ and Ti_2_Mg_3_Al_18_ compounds that strengthened the bond. Bond strength increased significantly from 15.6 MPa to 116 MPa as the bonding time extended to 60 min, reaching 180 MPa with an interlayer, and confirming high-quality joints without oxide layers. Ohashi et al. [465] developed a phase-replacement method to create new composites by selectively replacing phases through interdiffusion, demonstrated in joining Ti and Mg. The process generated a co-continuous microstructure and a reaction layer that enhanced joint strength, achieving a maximum strength of 116 MPa while suppressing interface fractures.

Overall, Al proves beneficial as a transition element in welding Ti and Mg alloys, enhancing bond quality. Diffusion welding outperforms friction welding for joining Mg alloys with reduced challenges, with key factors being temperature, holding time, and pressure.

#### 3.2.3. Explosive Welding of Mg Alloys

Explosive welding utilizes explosive impact to join materials [383]. It features brief peak pressure and low mutual diffusion between solids, often causing eddy currents and interface waves. The process possesses a complex nature, and thus its control is difficult. Explosive welding (EXW) has been effectively utilized to weld Mg/Mg [466], Mg/Al [467,468,469,470,471], Mg/Cu [472], Mg/steel [473,474], and Mg/Ti [475], resulting in joints with remarkable strength and resistance to corrosion. Nevertheless, its use in Mg–Li alloys is still mostly unexamined, particularly when paired with high-melting-point or refractory metals, as the interfacial behavior and weldability are not well understood [55]. Interestingly, Tian et al. [476] noted that Mg–Li alloys, while lightweight, lack surface hardness and corrosion resistance. A high-throughput explosive welding (HT-EXW) technique was developed to bond different metals to an LZ91 Mg–Li plate using a single detonation. The resulting Ta/LZ91 joints showed refined Ta grains and an asymmetric mixing layer, with joint hardness exceeding 75% of the original flyer’s hardness and surpassing the Mg–Li substrate’s hardness.

*Mg alloy/Mg or Al alloy*

During explosive welding of Mg alloys, the shock wave alters the grain structure and forms a vortex at the interface. Nan et al. [466] demonstrated this technology by connecting five Mg alloy plates, resulting in a wavy laminated structure. The interface experiences large deformations, refining the grain and optimizing joint performance, leading to increased hardness and periodic energy distribution compared to the BM. Inao et al. [477] used explosive welding to successfully bond thin plates made of Mg_96_Zn_2_Y_2_ and AZ31 alloys, enhancing corrosion resistance. Bonds showed wavy interfaces, with superior bonding quality compared to traditional methods. Mihara-Narita et al. [383] stated that welding dissimilar Mg and Al alloys is challenging due to brittle IMCs forming at the interface, weakening bonding strength. Explosive welding was employed to join these alloys, minimizing interlayer development. A thin γ-Mg_17_Al_12_ interlayer formed, affected by alloy composition and annealing, which increased interlayer thickness and led to crack initiation, decreasing shear strength. Corrosion resistance was slightly better than that of mechanically fastened samples. Su et al. [469] studied clad materials made from AA1100P-H24 and AZ31B-H24 via explosive welding as a Whipple shield for space applications. Two thickness ratios, Mg:Al = 0.5:0.5 and Mg:Al = 0.7:0.3, were compared to the AA6061-T6 standard. The clad materials showed 16.8% and 23.7% reductions in area density. Hypervelocity impact tests revealed delamination in the Mg:Al = 0.7:0.3 ratio, while the Mg:Al = 0.5:0.5 ratio demonstrated reduced ejecta production and comparable perforation to monolithic materials.

*Mg alloy/Steel*

According to [55], explosive welding fabricates steel and Mg alloy composite plates, enhancing corrosion resistance and joint performance through a unique wave-shaped structure and broad application potential. Overall, explosive welding enables effective connections between Mg and steel, although safety and procedure complexities necessitate specialized settings for execution [40]. Malakhov et al. [473] stated that Al–Mg alloys are favored in shipbuilding and railcar industries due to their strength and corrosion resistance. However, welding these alloys to steel faces challenges, particularly from IMCs and adiabatic shear bands (ASBs). The authors investigated the impact of ASBs and Mg_2_Al_3_ on explosive-welded bimetal mechanical properties using various microscopy techniques and mechanical tests. Findings show that ASB and Mg_2_Al_3_ zones increase with detonation velocity, enhancing tear strength and plastic properties after heat treatment.

*Mg alloy/Ti alloy*

Explosive welding technology effectively connects Ti alloys with Mg alloys, utilizing unique wave structures. The explosive quantity, specifications, and detonation point placement significantly influence joint morphology and microstructure. Zhao et al. [475] analyzed the microstructure and properties of a Ti/Mg alloy clad plate via explosive welding. The bonding interface featured both straight and wavy areas; straight areas showed element diffusion, while wavy areas contained a melting zone with light particles. Tensile tests indicated an 18% increase in UTS compared to the Ti sheet, and shearing strength ranged from 68 to 87 MPa. Microhardness was greater than that of the original sheets up to 300 μm, with increased hardness in wavy areas. Corrosion resistance was superior, showing a 24% increase in corrosion potential and significantly lower current density than the Mg alloy sheet, enhancing industrial applications.

Summarizing, explosive welding (EXW) of Mg alloys enables solid-state joining of dissimilar materials, particularly Mg/Al. It generates a wavy interface, enhancing bonding strength. The process utilizes a high-velocity flyer plate to remove surface oxides. Challenges include Mg’s low ductility. The use of gelatin media [477] or inert atmospheres [478] allows to reduce defects and the improvement of bonding strength. Contradictory findings in EXW of Mg alloys arise from their non-equilibrium nature [479]. Key contradictions involve interfacial bonding, where early studies indicate adhesive bonding [477,480,481], while others show defined reaction zones due to IMC formation [383,384,481]. Variability in impact parameters affects bonding quality [479], while excessive energy leads to thick IMCs or voids [477,479]. Post-weld processing, like hot-rolling, alters mechanical properties [467,482,483]. Additionally, variations in alloy composition impact performance due to Mg’s anisotropic nature and differing interfacial behaviors [50,476,484]. The EXW process causes high residual stresses and brittle compounds, resulting in low as-welded joint efficiencies (10–30%) [52]. Post-weld treatment can enhance efficiencies to 70–90%+ [470]. EXW of Mg alloys can eliminate hot cracking and porosity [52]. However, improper standoff distances may cause interfacial voids or brittle intermetallic compounds [383,476]. Parameters such as explosive load and detonation speed can be optimized for defect-free bonding, especially with lightweight metals like Aluminum or Titanium [476,485]. Hardness distribution shows peaks at the interface due to recrystallization, with minimal heat-affected zone softening [476,485,486].

#### 3.2.4. Friction Stir Welding

Mg alloys exhibit shortcomings in chemical resistance and weldability [55,487], prompting the development of ternary Mg alloys, particularly those in the AZ series with aluminum and zinc. AZ91 alloy is widely used in the aircraft and automotive industries due to high castability, low density, and favorable mechanical properties [488]. However, joining Mg alloys remains challenging due to their reactivity and flammability [411,489,490], leading to defects in conventional fusion welding, like cracks and porosities. Friction stir welding (FSW) has emerged as a promising solid-state technique, reducing solidification defects and offering advantages such as improved microstructure, high-speed welding, and reduced skill requirements [491], with research focusing on optimal parameters influencing joint properties. Particularly, shoulder diameter and tool rotation speed (TRS) were the most influential parameters, followed by welding speed (WS), in most of the analyzed cases [492]. Rajesh and Rajasekaran [493] examined the fundamental concept of friction stir welding and various elements of this process, including the impacts of different welding parameters on Mg alloy. FSW is primarily affected by various welding parameters such as TRS, WS, and tool geometry, among others. An increase in TRS and axial force (AF) led to a reduction in percentage elongation. Hardness in the ultra-fine grain heat-affected zone (UHAZ) exceeds that in the nugget heat-affected zone (NHAZ). Along with Al alloys, FSW has been effectively employed to connect other metallic substances, including Cu, Ti, steel, Mg, and composites. Due to their high melting point and low ductility, effectively joining high melting temperature materials using FSW has often been restricted to a limited range of FSW parameters. Various authors, i.e., [48,59,492,494], reviewed the FSW processing of Mg alloys. Based on [495], multiple methods exist for processing Mg alloys, with a need for improved joining techniques. Friction stir welding (FSW) offers solid-state joining, overcoming traditional flaws like porosity. The study examines FSW processes, Mg alloy categories, tool effects, welding parameters, and practical applications of FSW, highlighting its environmental and energy efficiency. According to [381,496,497], FSW offers significant enhancements in strength, ductility, fatigue, and fracture toughness compared to conventional welding methods. However, different joint arrangements might be utilized for FSW instead of butt and lap joint designs. Various configurations of tool materials, geometries, and welding variables for various Mg alloys are presented in Table 8. The geometry of the tool is crucial for friction stir welding (FSW), significantly impacting joint quality due to the tool pin profile, shoulder diameter, and material. Friction between the tool shoulder and workpiece primarily generates heat, making these dimensions vital over design characteristics. The scrolled shoulder surface enhances material mixing. FSW process parameters, including spindle angle, tool tilt, target depth, axial force, and insertion depth, along with TRSs and WSs, significantly influence weld strength. Material flow patterns are shaped by the tool pin’s design, welding temperature, flow stress, and axial force, which also affects temperature. Research opportunities remain in joint designs like edge butt, T butt, multiple lap, T lap, and fillet joints. FSW alters the BM microstructure, creating unique zones: stir zone (SZ), thermo-mechanically affected zone (TMAZ), and HAZ, each with distinct features. Residual stress develops, with the advanced side experiencing greater tensile stresses. Mechanical properties are influenced by FSW parameters, with increased hardness and strengthening effects observed in Mg alloys across various industries. The summarized mechanical properties of FSW Mg alloys are shown in Table 9.

**Table 8:** Various tool materials, geometries, and welding variables were used for FSW of several Mg alloys.

Workpiece Material	Tool Material	Tool Shape	Tool Size	Operating Parameters	Refs.
AZ31 pure and with dispersed reinforcement particles SiC, Al_2_O_3_, Cr, and Si powder; 8 mm thick	HSS; HRc 62	Cylindrical flat shoulder, Conical pin	Shoulder length of 80 mm; Shoulder diameter of 50 mm; Pin length of 4 mm; Pin diameter of 5 mm	TRS: 1000, 1200, 1400 rpm; WS: 40 mm/min	[498]
**Remarks**	Mg composites showed increased surface smoothness with higher TRSs. Maximum mechanical strength occurred at a TRS of 1000 rpm. Reinforcements improved the microstructural and mechanical properties over the AZ31 alloy, with a mean hardness of 32 HRB. Hardness increased by up to 50% in the FSPed region, while double-pass processing enhanced hardness and impact strength significantly. Finer microstructures resulted from the Si powder.				
AZ31 with TiC and Al_2_O_3_ NPs; 6 mm thick	NA	Cylindrical flat shoulder, Hexagonal pin	Shoulder diameter of 18 mm	AF: 10 KN; TRS: 1000 rpm; WS: 50 mm/min; Dwell Time: 5 s; Welding Length: 100 mm; Welding Depth: 5.5 mm	[499]
**Remarks**	The addition of TiC and Al_2_O_3_ NPs to AZ31 alloy enhances its mechanical properties, notably TS (241 MPa), YS (188 MPa), and impact energy (90.34 J) at 1.5% concentration, while reducing elongation (8.9%). Increasing the NPs content to 2% slightly decreases strength but improves elongation. Fatigue life improves markedly with 1.5% NSs (110,000 cycles) before declining at 2%, highlighting the need for optimal NPs concentration for performance.				
AZ61A; 6 mm thick	HCHCr	NA	NA	TRS: 1600–2000 rpm; WS: 20 and 40 mm/min	[500]
**Remarks**	At TRS of 2000 rpm and WS of 20 mm/min, the joint showed a weld efficiency of 89%. Such a joint with the highest specific thermal contribution (STC) (100 rpm/mm/min) exhibited the highest TS of 275 MPa, compared to about 310 MPa for the BM.				
AZ91; 6 mm thick	NA	Cylindrical flat shoulder, Cylindrical pin	NA	TRS: 355 rpm; WS: 112 and 140 mm/min; tilt of the tool: 1.5°.	[501]
**Remarks**	FSWed material experiences elevated temperature-induced plastic deformation, leading to dynamic recrystallization and grain refinement. Smaller grain size boosted TS (300 MPa) and microhardness (80 HV), while the affected specimens show ductile fracture. Nugget zone temperatures can exceed 400 °C.				

**Table 9:** The summarized mechanical properties of FSW Mg alloys.

Workpiece Material	Plate Thickness (mm)	BM			FSWed Zone			Joint Efficiency (%)	Refs.
		UTS (MPa)	YS (MPa)	Hardness (Hv)	UTS (MPa)	YS (MPa)	Hardness (Hv)		
AZ31B-O	3.1–5	206–230	104	50–52.2	140.8–216; 232 (AFSW)	94; 97 (AFSW)	56.3–64.77; 80 (AFSW)	68–94; 101 (AFSW)	[502]
Mg–2Nd-0.3Zn–0.4Zr (NZ20K)	2	210	NA	60–65	191	NA	73–85	NA	[503]

AFSW: additive friction stir welding.

**Figure 3:**
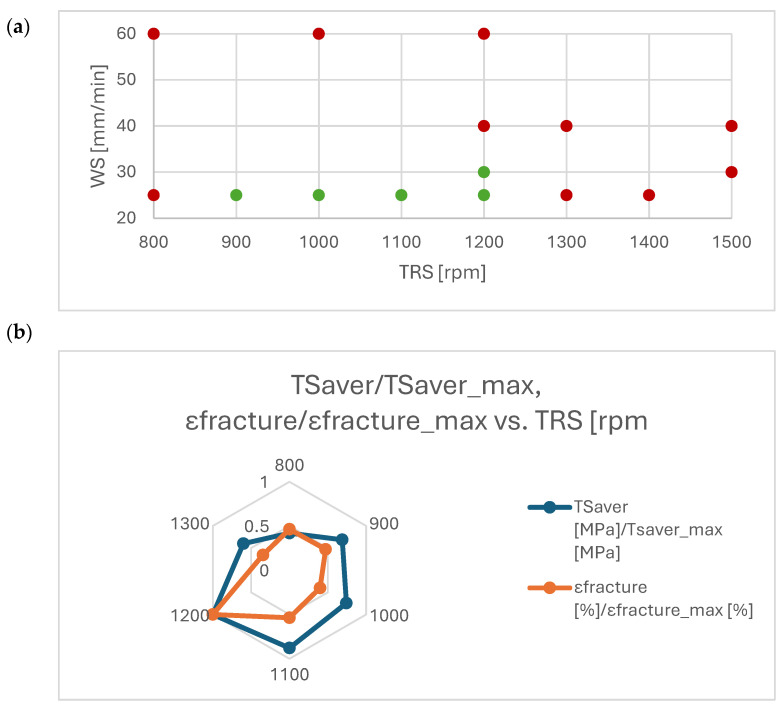
(**a**) Conventional FSW parameters window for joining the dissimilar Mg/Al (based on data from [524]). Red—defective process parameters. Green—defect-free process parameters. (**b**) Influence of TRS on the TS and elongation of the conventional FSWed dissimilar Mg/Al (based on data from [48,524]).

TRS significantly influences material heating, flow, and mixing in FSW, directly affecting joint properties and quality, especially in dissimilar Mg/Al FSW. Kumar et al. [504] noted that for FSW of AZ31B/AA6061-T4 alloys, TS peaked at 130 MPa before dropping to 63 MPa at 1000 rpm. Badkoobeh et al. [492] examined FSW and FSP of Mg-based alloys, highlighting advancements in grain refinement mechanisms, influential parameters, microstructural changes, and mechanical properties. They noted that the treatment parameters and tool geometry notably impact FSW/FSP mechanisms, leading to significant microstructural alterations, including texture changes that can soften or harden the material. FSW eliminates defects associated with fusion welding, making it superior for Mg alloys, which are prone to issues like coarse grains and brittle IMCs. These solid-state methods can transform material surfaces into composite structures, free from flaws related to casting. Enhanced properties, such as fine-grained structures and improved strength, ductility, and toughness, are key advantages. Tool geometry and material selection are crucial for optimizing alloy properties during FSW/FSP. Proper analysis of these aspects can provide insights into their impact. Research should explore various joint designs beyond butt and lap types. The complexities of severe plastic deformation, frictional heat, and material flow necessitate detailed examinations of FSW/FSP mechanisms. Dynamic recrystallization (DRX) is vital for grain refinement, warranting thorough investigation. Due to significant residual stresses from FSW/FSP, post-heat treatment may enhance performance; heat treatment prior to and during processing is recommended. Utilizing different reinforcements can create composite structures in Mg alloys. However, wear, fatigue, creep, and corrosion interactions with mechanical factors in Mg-based alloys and composites formed via FSW/FSP remain underexplored, suggesting areas for further research. Desai et al. [505] conducted a study on the FSW of AZ31 alloy, highlighting that the tool pin’s diameter and profile, tool material, and shoulder diameter significantly affect joint properties. They found that heat generation primarily arises from friction between the workpiece and tool shoulder, making tool dimensions vital over design features. Key factors for high-quality welds included TRS, WS, tilt angle, SF, and target height. AF governs welding temperature, while tool pin geometry and flow stress influence material flow patterns. There is notable research potential for T butt joints, T lap joints, edge butt joints, multiple lap weld joints, and fillet weld joints. The FSW process alters the BM’s microstructure, generating distinct SZs, TMAZs, and HAZs. Residual stresses vary, with TMAZ and SZ showing tensile stresses and HAZ exhibiting compressive stresses, affecting post-weld mechanical characteristics. Derazkola and Kubit [506] compared friction stir welding (FSW) and underwater friction stir welding (UFSW) of AZ31 alloy in T-configuration, highlighting the impacts on heat distribution, material characteristics, and mechanical performance. Simulation outcomes demonstrated a more consistent heat distribution in both welding methods, with the highest temperature located on the advancing side. The highest temperatures observed at the shoulder–workpiece interface were 404 °C for FSW and 349 °C for UFSW, indicating a 13.6% decrease in UFSW. The speed of the material at the trailing edge was 63 mm/s for FSW and 42 mm/s for UFSW, indicating a 34% reduction due to decreased heat production in UFSW. The strain rates were 450 s^−1^ for FSW and 420 s^−1^ for UFSW. Grain size in the SZ measured 26 µm for FSW and 21 µm for UFSW, representing a 19% decrease. UTS rose by 6% in the skin direction and 12.8% in the flange direction for UFSW relative to FSW. Enhanced ductility was noted in fractures from UFSW. These findings showed that UFSW is more effective in enhancing thermal management, microstructural characteristics, and mechanical performance of welded connections. Türkan et al. [507] examined the welds of the 5.2 mm thick AZ31 alloy utilizing conventional friction stir welding at optimal joining efficiency. The experiments resulted in an 88% joining efficiency in TS achieved at 1250 rpm and a welding parameter of 400 mm·min^−1^. For the samples combined with these parameters, the joining was completely achieved. Samples combined with these parameters have been utilized in fatigue tests. Based on the results of the strain-controlled low-cycle fatigue tests conducted on welded and BM specimens, the BM samples surpassed the 50,000-cycle threshold without failure, exhibiting an elongation percentage of 0.3%, while the welded samples displayed an elongation percentage of 0.2%. Parameters for low-cycle fatigue of welded and BM samples have been acquired using the Coffin–Manson–Basquin equation. Hima Silpa et al. [508] examined the impact of FSW parameters, particularly the roles of AF, WS, and TRS, on the hardness and corrosion potential of the AZ31B alloy. They discovered that the best parametric set affecting the responses consisted of a tool tilt angle of 1.5, an AF of 6 kN, a WS of 55 mm/min, and a TRS of 600 rpm. Yan et al. [509] conducted combined numerical simulations and experiments analyzing the thermal, mechanical, and microstructural changes in AZ31 alloy during FSW, focusing on how parameters like TRS and WS affected temperature distribution, grain refinement, and mechanical properties. A notable temperature increase occurred when the tool’s shoulder engaged with the weld, with the advancing side (AS) exhibiting slightly higher temperature, stress, strain, and grain size than the receding side (RS). Adjusting TRS to 1200 rpm and reducing WS to 200 mm/min significantly reduced the weld’s initial grain size from 57.8 μm to 8.2 μm. Akçakale [59] examined numerous advancements in FSW of Mg alloys, focusing on tool design, process optimization, microstructural changes, corrosion resistance, mechanical properties, and post-weld heat treatment (PWHT). They noted that advanced techniques like cryogenic and submerged FSW promote ultrafine microstructures. Key challenges included brittle IMCs, residual stress control, and tool design complexities, while promising solutions involve optimized parameters, advanced tool materials, hybrid joining techniques, and innovative post-processing methods. Scalability and defect prevention remained critical challenges. Singh et al. [491] connected AZ31 alloy plates using FSW, revealing a TS of 145.4 ± 4.9 MPa and elongation of 9.5 ± 0.9%. Post-welding heat treatment (PWHT) enhanced TS by 4.74% and elongation by 15.78%, while reducing microhardness in the SZ by 6.84%. PWHT improved grain homogenization and uniformity in hardness distribution, with toughness measuring 4.5 ± 0.17 J. Ductile failure was observed in both joint types. Kaushal et al. [510] also indicated that post-weld heat treatment (PWHT) is an effective method for eliminating residual stress, reinstating the properties of the FSW joint of parts made of Mg alloy, and enhancing its performance. Karwande and Seeram [511] joined 5 mm thick samples of AZ91 and A31 alloys using FSW with a taper tool. Optimal parameters for AZ31 showed the highest UTS, YS, and elongation at TRS of 1400 rpm and WS of 70 mm/min. Increased TRS enhanced mechanical properties, while WS had a less clear impact. For AZ91, TRS increased UTS and YS but had complex effects on elongation, with the lowest values found in dissimilar joints. Wang et al. [494] investigated the thermal and mechanical effects during FSW of Al–Zn–Mg alloys on second-phase particle development in weld areas. Soluble particles dissolved in the SZ and TMAZ, enhancing toughness, while insoluble particles were redistributed. Dispersoids remained stable, preventing recrystallization and serving as nucleation sites for strengthening precipitates. Fine coherent η′ precipitates significantly strengthened the alloys. FSW created a range of peak temperatures, affecting microstructures across weld zones. The SZ reached the highest temperature, dissolving η′ phases and reducing hardness. However, recrystallization and reprecipitation post-welding might mitigate hardness loss. In the TMAZ, some η′ phases partially dissolved, while the HAZ contained coarsened η phases, leading to softening effects. According to [512], FSW is a reliable method for joining Al–Mg alloys, addressing challenges through optimized parameters and PWHT to enhance joint integrity. Expanding weld configurations and exploring variants like back heating and laser-assisted FSW can improve weld quality. μFSW shows potential for specialized applications. However, further research is needed in this area. Yang et al. [513] employed a CFD simulation using a thermo-mechanical-flow model to predict material behavior during dissimilar FSW of AA6061 alloy and AZ31B alloy. The primary motion involved rotation around the tool pin in the high-velocity zone (HVZ), with a vertical velocity component under 10% of the total. The process caused significant material redistribution, with the Mg alloy migrating to the lower joint and the Al alloy to the upper. Material transfer in the thickness direction led to straight-through movement in the HVZ, while some Mg alloys exhibited a swirling pattern around the tool pin. Kellai et al. [514] explored how high TRS and WS affect the microstructural and mechanical properties in FSW of AZ31-AZ61 alloys. Observations included dynamic recrystallization and grain refinement in the SZ, along with precipitate particles and intergranular compounds. The TMAZs showed larger restored grains, while the HAZs resembled the BMs. Microhardness peaked at 60 HV for AZ31 and 68 HV for AZ61, with UTS increasing significantly. Wang et al. [515] addressed coarse grains and poor mechanical properties in Mg alloy fusion welding by designing microstructures with fine, uniform grains and particle reinforcement. This was achieved through powder feeding with fusion welding and friction stir processing, reducing grain size from 193.5 μm to 15.9 μm (91.8% refinement), increasing TS by 70.2 MPa, and enhancing elongation by 136%. Fine-grain strengthening and particle effects improved the joint’s TS and hardness, while grain refinement enhanced elongation. Temperature distribution in FSW is crucial for the mechanical properties and microstructure of welds. Xu et al. [516] enhanced YS in AZ91 alloy via pre-aging and high-force FSP, observing grain refinement and precipitate strengthening in the SZ. Hassanamraji et al. [517] simulated deformation and heat transfer in FSP of AZ91 alloy, analyzing tool forces with different pin shapes. Hoda et al. [518] investigated tool speed and rotation effects on thermal distribution, achieving a defect-free sample at 1200 rpm and 60 mm/min. The ratio of WS to TRS, known as revolutionary pitch (RP), affects heat input during friction stir welding (FSW), influencing the microstructure and properties of the weld zone. Yousefpour et al. [519] reported the impact of heat input on the microstructure and grain size of AZ 91 alloy. Desai [520] noted that nearly defect-free AZ91 FSW joints could be achieved with TRSs of 765–1500 rpm and WSs of 78–195 mm/min. The TRS significantly impacted peak temperature, grain morphology, intermetallic formation, and mechanical properties, with a direct correlation between defect omission and joint properties. Specimens with a revolutionary pitch of 0.05 to 0.13 displayed finer grain sizes and higher peak temperatures, influencing heat flux during FSW. High TRSs resulted in superior properties due to enhanced dynamic recrystallization, achieving a joint efficiency of 85% at 1500 rpm and 78 mm/min, attributed to fine grain morphology and peak temperatures that dissolved brittle intermetallics. Fractography showed ductile fractures for optimal conditions versus mixed fractures for BM. Low TRSs with high WSs led to defects such as porosity and voids. Microhardness aligned with tensile results, revealing a marginal increase of 20% over BM for the highest speed. Joint strength weakened with compressed air cooling and Cu backing due to material flow defects, while water and CO_2_ cooling yielded defect-free joints but compromised strength, with water cooling achieving low peak temperatures and good grain refinement compared to CO2’s high peak temperatures and coarser grains. Cooling methods generally resulted in lower hardness, with isolated peaks detected in specific areas. Kim et al. [502] developed an additive friction stir-welding (AFSW) process to enhance the mechanical strength and corrosion resistance of AZ31B weld joints using fine Al powder. In this solid-state process, pure Al powders react in a machined groove of the AZ31B matrix. Optimization involved changing TRSs and WSs, the number of passes, and tool types, testing three groove configurations. Optimal parameters were 1400 rpm, 25 mm/min, four passes, and inserted symmetric grooves with a square pin tool. Resulting properties included 80 HV microhardness, 101% joint efficiency, 8.9% elongation, and a 55% improvement in corrosion resistance over FS-welded joints. As stated in [521], FSW of Mg alloys alters microstructures and introduces cracks and oxides, affecting joint strength and efficiency, peaking at 90% of the BM’s strength. The best corrosion rate (0.22 mm/year) occurred at 700 rpm, 30 mm/min, with an 18 mm shoulder diameter. Feng et al. [522] noted that FSW is effective for bonding Mg alloys, with the generated temperature influencing joint properties like residual stress and microstructure. A revised heat source model, considering friction shear stress on the tool’s surfaces, was developed and applied throughout the welding process. Higher TRS or lower WS significantly increases temperature, though this effect diminishes at high TRS and WS. Arya et al. [523] noted that FSW of Al and Mg alloys faced challenges due to distinct crystal structures, leading to IMC formation at weld points. This occurred primarily within the banded structure area, a weak point causing joint failures. High TRS and low WS exacerbated intermetallic phases. However, suitable mechanical properties existed if the phase thickness was under ten μm, with strategies like cooling mediums and barrier layers used to reduce compounds. Ahmed et al. [48] reported that welding dissimilar alloys like Mg and Al faced challenges due to fragile IMCs, particularly Al_3_Mg_2_ and Al_12_Mg_17_ phases. FSW helped mitigate IMC issues by utilizing low heat inputs. Key research focused on weldability, morphology, and mechanical properties while minimizing heat to reduce IMC formation. Techniques like using Zn interlayers, electric current-assisted FSW, and ultrasonic vibration FSW were effective in controlling brittle IMC development. Xiaoqing et al. [524] studied the combined influence of FSW parameters on obtaining free defect welds (Figure 3a). They found that a low FSW speed of 25 mm/min yielded defect-free joints between AZ31B/AA6061. Increasing the tool rotation rate to 1200 rpm enhanced TS to 162 MPa and elongation to 5.5%, but both declined at 1300 rpm (Figure 3b). Conventional FSW parameters window for joining the dissimilar Mg/Al is presented in Figure 3a, based on data from [48,524].

**Figure 4:**
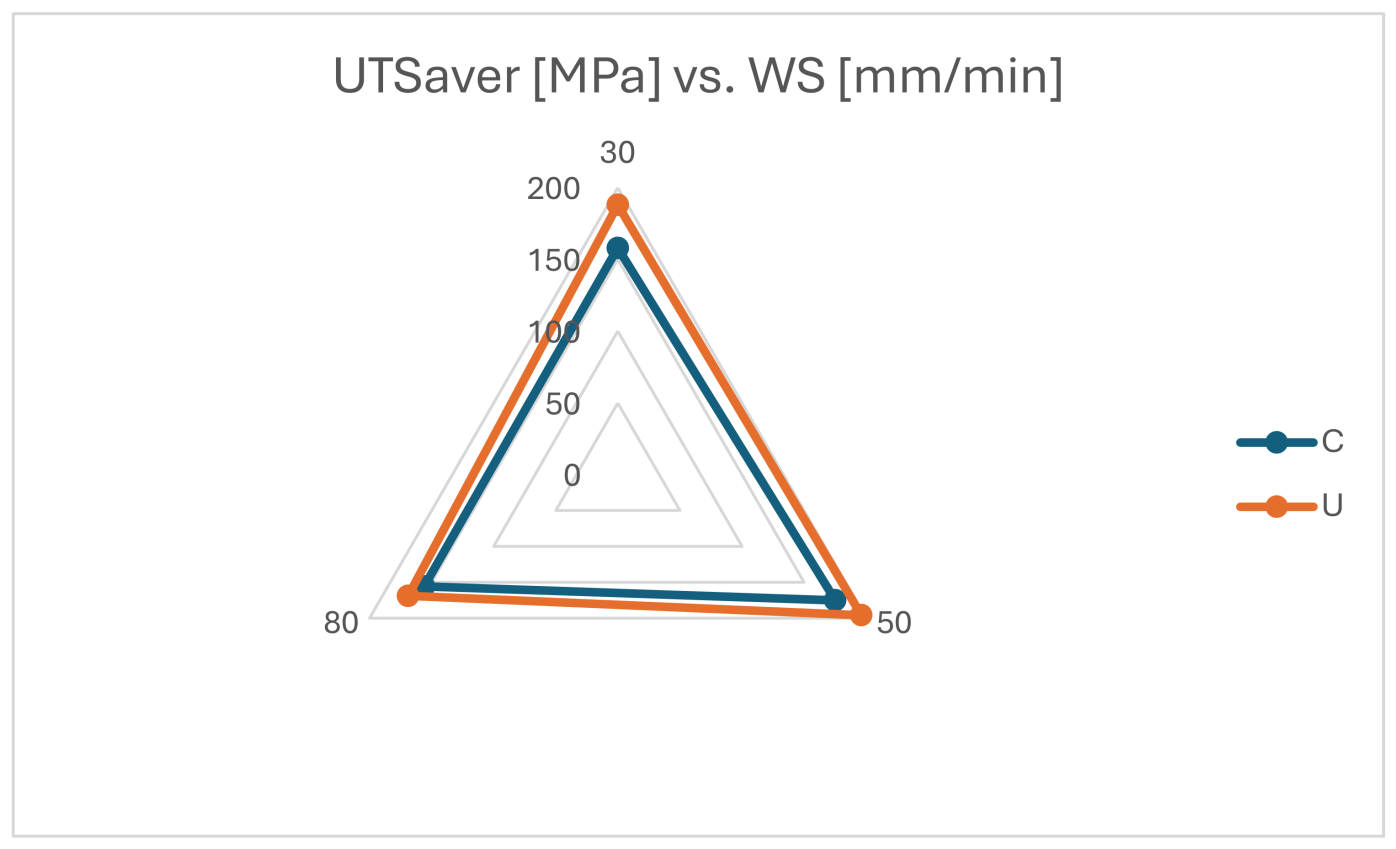
Tensile strength of FSWed and UVaFSWed AZ31B-H24/AA6061-T6 alloys at a constant TRS of 800 rpm at different WSs (based on data from [525]).

Zhao et al. [525] studied the effect of welding speed (30–80 mm/min) at a constant rotation rate of 800 rpm. They found that increasing the speed from 30 mm/min to 50 mm/min improved TS from 158 MPa to 175 MPa, but higher speeds reduced it to 156 MPa due to excessive heat and increased IMCs. The optimal welding speeds for conventional FSW (C—in Figure 4) and UVaFSW (U—in Figure 4) were 50 mm/min, achieving TSs of 175 MPa and 196 MPa, respectively. Kumar et al. [526] noted that lower welding speeds are needed at lower rotation rates (400–600 rpm), whereas higher speeds (100–150 mm/min) are required at higher rates (800–1200 rpm) for acceptable joints. Kumara and Balasubramanian [527] confirmed these findings.

**Figure 5:**
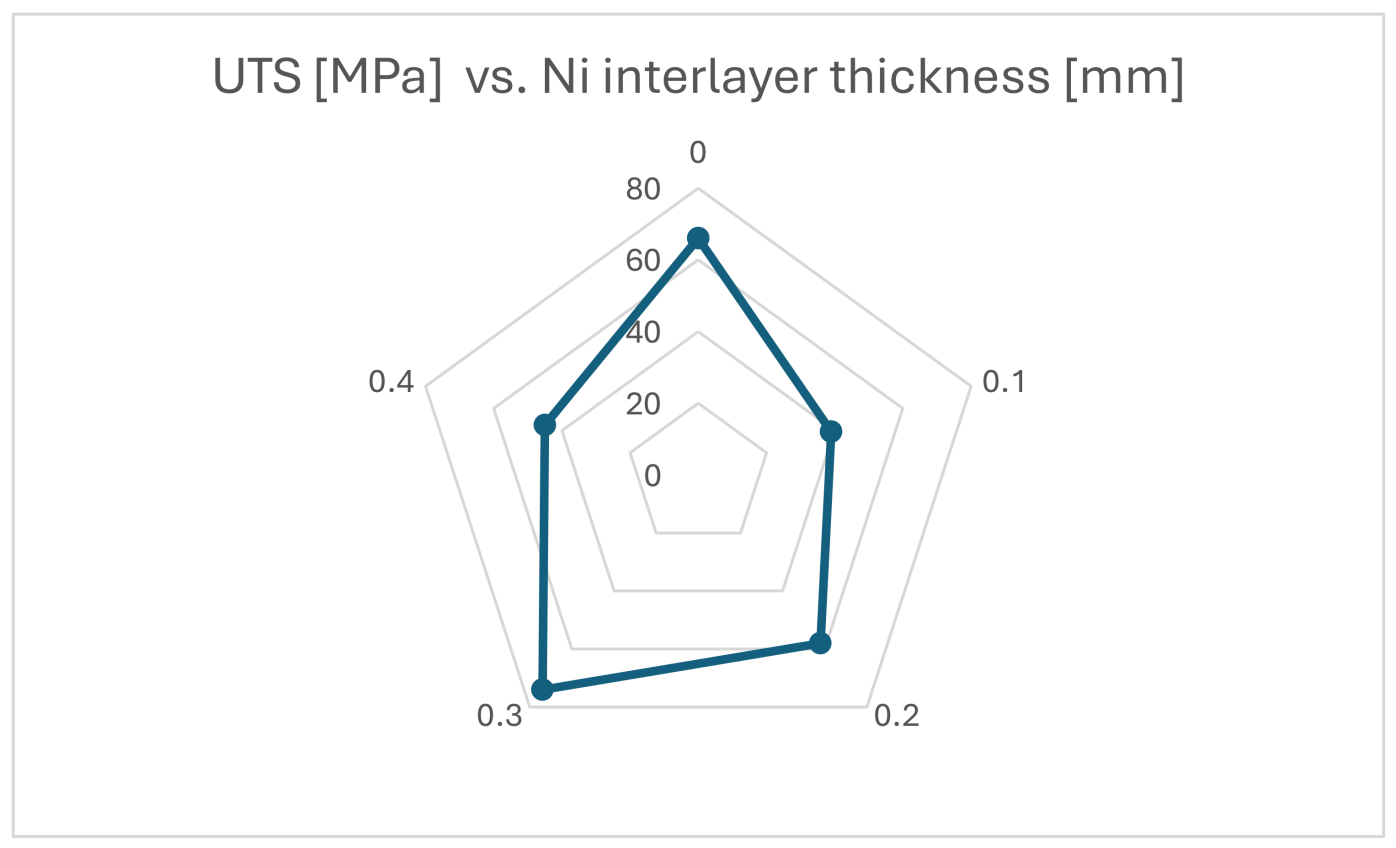
Tensile strength variation with the thickness of the Ni interlayer (based on data from [528]).

Dong et al. [528] studied the effects of a Ni interlayer on the microstructure and properties of FSWed AZ31B and AA6061 joints. The Ni-free joint exhibited a vortex-like structure near the bottom and a banded structure at the top. In contrast, the Ni interlayer joint showed a typical onion ring structure, characterized by a complex banded interlayer. While the Ni interlayer did not prevent the formation of Al-Mg brittle IMCs, it did introduce Mg_2_Ni IMCs. The Ni interlayer improved TS by 13% (Figure 5), elongation by 1%, and hardness by up to 30%.

According to [529], at present, studies on the corrosion of Mg alloy FSW joints primarily concentrate on improving the corrosion resistance of these joints through alterations in compositions, structural changes, and surface coating techniques. The adjustment of welding process parameters can refine the grains, thereby reducing the impact of the second phase on the alloy’s resistance to corrosion. According to [502], the reliability of welds for Mg alloys depends on both their mechanical and corrosion properties. Particularly, the corrosion resistance of AZ31 alloys is influenced by microstructural factors, including grain size, twin boundaries, texture, dislocation density, second-phase particle distribution, and residual stress [118,530,531,532]. Safari et al. [533] studied FSWed AA5052 and AZ31 alloys, optimizing parameters at a TRS/WS ratio ranging from 20 to 38 rpm/mm·min^−1^. Increased TRS resulted in IMC formation and higher corrosion rates in the SZ. At TRS of 750 rpm, the corrosion rate reached 1.6 mA·cm^−2^, significantly higher due to galvanic coupling effects. The corrosion resistance of FSWed AZ61 joints was discussed in [534]. Padmanabham et al. [535] assessed tool pin profiles’ effects on AZ61 alloy’s microstructure and corrosion during friction stir welding, and found that the square pin showed optimal results in refinement and resistance. Lingampalli et al. [536] studied the microstructure and corrosion behavior of FSW butt joints of ZM21 alloy, highlighting the impact of TRS and WS on grain size, stresses, and corrosion resistance in 3.5 wt.% NaCl, revealing complex interdependencies. Nakatsugawa et al. [537] examine the corrosion performance of linear friction-welded AZ91 and AZ91+2%Ca alloys in 1 wt.% NaCl solution. They found that dissimilar welds (AZ91/AZX912) had 10% higher corrosion rates than similar ones. The weld zones remained unchanged, acting as a bridge without affecting corrosion. Sindhu et al. [538] investigated the corrosion behavior of FSWed joints of AZ91 alloy, finding that square pin profiles significantly reduced corrosion rates due to improved material flow and surface morphology modifications, enhancing β-Mg17Al12 distribution. While one study indicates that grain refinement improves corrosion resistance in FSWed AZ31 joints [539], others attribute this improvement to the distribution and size of second-phase particles and texture modification from friction stirring [540]. One report highlights a deterioration in corrosion resistance due to variations in grain size and texture effects [541]. Given the ambiguity regarding the impact of FSW on corrosion resistance, additional coating methods like plasma electrolytic oxidation [318,323,542,543], micro-arc oxidation [544,545,546,547,548,549], and cold spray [550,551] are recommended. According to [529], corrosion resistance of Mg alloy FSW joints can be improved through composition changes, structural modifications, and surface coatings, while refining grain sizes by adjusting welding parameters to minimize second-phase effects.

In summary, during the FSW process, severe plastic deformation occurs due to frictional heat and tool stirring [505], leading to the solid-state joining of softened BM. This unique material flow results in an inhomogeneous microstructure and crystallographic texture in the weld joint [552,553]. The texture’s heterogeneity in FSWed AZ31 joints promotes basal slip in the thermo-mechanically affected zone (TMAZ) and HAZ but restricts it at the weld zone center during transverse tensile tests, leading to strain localization and reduced mechanical strength compared to the BM [505,554]. Weld quality, indicated by joint efficiency, is influenced by process parameters such as tool speeds [52,553], number of passes [505], and tool geometry [52,553]. According to [492], the prevalent parameters affecting weld quality and ductility include TRS, WS, tilt angle, tool offset, AF, tool profiles, pin length, pin diameter, shoulder diameter, shoulder diameter to the pin diameter ratio, shoulder morphology, shoulder angle, pin morphology, pin angle, rotation direction, cooling media, the geometry of shoulder and pin, sheet thickness, and chemical composition. Innovations like bobbin-tool FSW [555,556,557] and stationary shoulder FSW [558] have been introduced for improved joint reliability, particularly in the case of AZ31. Mg alloys typically exhibit low formability, with sheet materials produced through casting or die casting, except for certain wrought alloys like AZ31. Welding these cast alloys poses challenges due to porosity and substantial distortion caused by their high coefficient of expansion, making solid-state welding techniques preferable [381]. FSW has been researched on alloys such as AM50, AM60, ZK60, AZ31, AZ61, and AZ91, using plates between 1.5 mm and 6.5 mm thick for different joint configurations. Tool parameters include shoulder diameters of 6–20 mm, pin diameters of 3.175–7 mm, and probe lengths of 4–5.8 mm. WSs varied from 22 to 2000 mm/min, with TRSs between 400 and 2000 rpm and AFs from 3 to 22 kN. The quality of welded joints is sensitive to TRS and WS, with three distinct microstructural zones identified, leading to superior hardness and mechanical properties in the SZ [50,381].

#### 3.2.5. Friction Stir Spot Welding

Incorporating Al and Mg alloys into multi-material systems necessitates strong joining methods to guarantee structural integrity [559]. Combining these different alloys poses a considerable technical obstacle because of their fundamental metallurgical incompatibility. The main problems arise from the creation of brittle IMCs, like Al_3_Mg_2_ and Mg_17_Al_12_, at the welding joint interface. These IMCs affect mechanical properties, resulting in early failure when subjected to load [384,560]. The significant variations in thermal characteristics, including melting points, thermal expansion coefficients, and thermal conductivity between Al and Mg, worsen residual stresses and cracking. The stable oxide films on the alloy surfaces also function as obstacles to proper metallurgical bonding. As a result, solid-state bonding methods, comprising friction welding, diffusion welding, explosion welding, ultrasonic welding, and cold welding, have become essential alternatives for welding dissimilar Al–Mg, which can alleviate the mentioned problems. Friction Stir Spot Welding (FSSW) has become a significant solid-state joining method for Al and Mg alloys [561]. This method functions beneath the melting temperature of the BMs, utilizing frictional heat and mechanical deformation to form a metallurgical connection [562,563]. This method decreases the creation of fragile IMC, lessens residual stresses, and prevents defects associated with solidification [564]. The FSSW method uses a rotating tool that penetrates the overlapping sheets, creating localized heat and softening the material [565]. The softened substance is subsequently forged under pressure to create a weld nugget. According to [566], current advancements in FSSW highlight the interplay of microstructure, properties, and performance under load. FSSW surpasses traditional welding by effectively joining similar and dissimilar materials. Key parameters like tool design, TRS, AF, and dwell time influence weld quality. Innovations in robotics and numerical simulations enhance FSSW accuracy and efficiency, while nano/microparticle additives improve strength and thermal stability in composite welds.

*Mg alloy/Mg alloy*

Joining Mg alloys is challenging due to their HCP structure and oxidation risks during welding [567]. Golroudbary et al. [568] analyzed Mg’s lifecycle in the automotive sector, noting trends in production and recycling. For lightweight applications, FSSW provides a solid-state joining solution, addressing high reactivity and low ductility issues. Mg alloys are lightweight yet strong, ideal for structural uses, necessitating optimization of FSSW parameters. Recent research has examined the effects of RS and DT on the effectiveness of FSSW for Mg alloys. Sarikavak’s [569] FEA on AZ91 alloy shows high temperature rises with tool speeds, peaking during dwell, and highlights risks of grain-boundary cracking at moderate speeds. Advances in tool design have improved FSSW performance for Mg alloys. Prihajatno et al. [570] found that increasing tool speed enhanced the UTS of AZ31B-H24 alloy, peaking at 222.6 MPa at 2280 rpm. Liu et al. [571] studied refill friction stir spot welds in 1 mm-thick AZ31 alloy sheets, achieving the highest lap-shear strength at 1500 rpm and a 1.4 mm PD. A study by Fu et al. [572] developed a differential-rotation RFSSW technique for cast Mg alloys, leading to discontinuous dynamic recrystallization and a bimodal, weakened microstructure. This process enhanced lap-shear strength by 50%, changing the failure mode to stir-zone pull-out. M. Rakshith’s [573] review on AZ31 alloy deformation emphasizes grain size refinement as crucial for improving the alloy’s low as-cast strength. It compares conventional bulk techniques with severe plastic deformation methods, yielding nanoscale grains. The review also highlights FEA as useful for simulating strain fields and optimizing processes.

*Al alloy/Mg alloy*

Shah [564] reported that the joining of dissimilar Al and Mg alloys through Friction Stir Spot Welding (FSSW) presents challenges, particularly due to the formation of brittle IMCs like Al_3_Mg_2_ and Al_12_Mg_17_ at the weld interface. These IMCs, including Mg_2_Al_3_ and Mg_17_Al_12_, compromise the weld’s strength and ductility. Limited solubility of Mg in Al complicates the nugget zone microstructure, while the inherent brittleness of these IMCs undermines joint integrity and fracture resistance. Additionally, the differing crystal structures and thermal properties of Al and Mg create complexities in achieving uniform material flow and heat distribution, resulting in potential localized weaknesses, with keyhole defects acting as stress concentration sites. Galvanic corrosion at the Al–Mg interface poses further risks to long-term durability. A nuanced understanding of metallurgical and thermomechanical phenomena is essential for effective FSSW of these dissimilar materials. Research by Ahmed et al. [574] highlighted that higher tool rotation speeds refined grains and promoted favorable shear textures in AA5052-H32 welds, indicating sensitivity of Al–Mg alloys to thermomechanical conditions. The initial large, randomly oriented grains undergo recrystallization during FSSW, resulting in smaller, uniform grains; optimizing parameters like tool speed can significantly enhance microstructural control and weld integrity. Recent developments in FSSW for Al–Mg dissimilar joints have centered on refining process parameters and tackling interfacial issues [575]. Crucial factors like tool revolution per minute (RPM), plunge depth (PD), dwell time (DT), and tool shape have been thoroughly analyzed to optimize the balance between heat production and material movement [561,576]. Innovations like interlayer-assisted FSSW have shown potential in addressing problems associated with IMC formation. Slim metallic layers placed between Al and Mg sheets function as diffusion barriers, inhibiting detrimental IMC formation while encouraging the emergence of more ductile interfacial phases [577]. These interlayers improve mechanical strength and resistance to corrosion by modifying the interfacial chemistry. Hybrid methods that integrate FSSW with strategies such as preheating, ultrasonic vibration, or post-weld heat treatment have enhanced joint performance by optimizing microstructures and alleviating residual stresses [578,579]. Significant focus has been directed towards dissimilar Al–Mg welding, where the creation of rigid IMC continues to be a major challenge [580]. Feng et al. [581] examined recent progress in RFSSW for welding Al and Mg alloys, including both similar and different materials. The welding process was examined, covering the joining mechanism, properties, microstructural attributes, and mechanical characteristics of the finished joints. The assessment also included advancements in welding equipment and the uses of this method. Wu et al. [582] utilized two-stage Swing Friction Stir Spot Welding (SFSSW) to join AA5083 with AZ31B alloys, noting improvements in bonding area and material mixing over conventional FSSW. The IMC’s thickness near the original keyhole increased due to higher heat input, with irregular bonding interfaces in SFSSWed joints. IMC configuration was spherical, unlike the dendritic structure in traditional FSSW. Additionally, the lap-shear tensile load for SFSSWed joints was 72.95% higher than for standard FSSWed joints. Beidokthi et al. [583] performed FSSW on AA6061-T6 and AA 7072-T6 alloys with a pinless tool. Microstructural analysis revealed Mg–Si and Fe–Si–Al precipitates in AA6061 and Mg–Si, Mg–Zn (η), and Al–Mg–Zn in AA 7072. A retrogression-like process occurred in AA 7072 welds. Each alloy exhibited an optimal dwell time for maximum weld strength. Hardness decreased in the SZ for AA6061 but increased for AA 7072. The highest tensile-shear loads achieved were 2277 N for AA6061 and 2539 N for AA 7072, respective dwell times being 6 s and 8 s. Rajendran et al. [584] used solid-state Friction Stir Spot Welding (FSSW) to create dissimilar welds between AZ31B and AA6061-T6 alloys. Optimal parameters yielded a maximum tensile shear fracture load (TSFL) of 3.61 kN with a tool rotational speed of 1000 rpm, plunge depth of 16 mm/min, dwell time of 5 s, and a tool diameter ratio of 3.0. The study conducted by Manickam et al. [585] on Friction Stir Spot Welding (FSSW) of AA6061 and AZ31B identified how process variables like rotation speed, plunge rate, dwell time, and shoulder-to-pin diameter ratio affect shear fracture stress. Optimal conditions included 1000 rpm rotational speed, 16 mm/min plunge rate, 5 min dwell time, and a 2.5 ratio, leading to increased shear fracture strain. The SZ showed maximum hardness, while the transition zone had the lowest. Three failure modes—eyelet, partially curved, and nugget pullout—were observed, with nugget pullout joints demonstrating maximum strength due to IMC formation. Sarila et al. [586] created dissimilar friction stir spot welds (FSSW) between AZ31B and AA 6061-T6 alloys using refill Friction Stir Spot Welding. Macrostructural and mechanical properties were evaluated for identical Mg-to-Mg and dissimilar Mg-to-Al joints, with comparisons to conventional methods. Hardness profiles of similar welds showed a W-shape, while the TMAZ and HAZ exhibited lower hardness than the rest of the weld. An IMC layer of Al_12_Mg_17_ was found at the Al–Mg interface, increasing hardness. Static shear strength of refill welds was significantly higher than that of traditional methods, revealing two distinct failure scenarios.

*Refill/reverse FSSW*

Sarila et al. [586] explored refill and conventional Friction Stir Spot Welding (FSSW) of AZ31B Mg and AA6061-T6 Al alloys at tool speeds of 900 to 1800 rpm. They found a thin, hard Al_12_Mg_17_ intermetallic layer at the Al–Mg interface, with RFSSW producing notably stronger joints than conventional methods. Shen et al. [587] examined RFSSW on similar Al–Mg joints at 1000, 1500, and 2000 rpm, reporting a peak lap-shear strength of 3.6 kN at 1000 rpm and a hardness drop in the heat-affected zone. The failure mode shifted from interfacial separation to nugget pull-out as speed increased, highlighting the role of rotating speed (RS) affecting joint durability. Rajendran et al. [588] combined experimental and statistical approaches to FSSW of AA2024-T6 to AZ31B alloys, identifying optimal parameters for maximum tensile shear load, with tool RS emerging as the most significant factor influencing joint strength.

*Interlayer insertion*

Interlayer strategies in Friction Stir Spot Welding (FSSW) show promise in diminishing brittle IMC and enhancing joint performance, though less is known compared to linear FSW. Research has borrowed techniques from linear FSW for FSSW applications. Dewangan et al. [589] discovered that using horizontal Zn interlayers in AA7075 and AZ31 FSSW achieved a TS of 126 MPa, surpassing the 85 MPa from vertical layers. Thin Zn interlayers have been shown to enhance Al–Mg and Al–Cu welds [590,591], while TiC additions improve hardness and TS. Additionally, using a pure Mg foil interlayer in Cu-Al FSSW increased TS by 30%. The use of Ni foil minimized IMC formation, albeit with lower microhardness [592]. Effective interlayer selection is critical for optimizing mechanical properties in welding applications.

*Pilot-hole assisted plunging*

According to [53], pilot-hole pre-drilling in Friction Stir Spot Welding (FSSW) offers benefits similar to those in linear friction stir welding (FSW) but remains underexplored. The pilot hole improves plasticization, controls tool penetration, and reduces brittle IMCs. Research by Sofian & Shah [593] indicates pre-drilling can reduce plunging forces by 50%, avoiding excessive downward pressure and keyhole formation. The other experiments [594] found that a 4 mm pilot-hole diameter at 800 rpm yielded a shear strength of 89.29 MPa. Hardness assessments showed that the pilot-hole size accounted for 97% of strength variations, with the AA7075 alloy enhancing interlocking and bonding. Rahim et al. [53] reported that adding layers of Zn, Ni, and Mg has resulted in increased strength; for instance, a Ni layer boosts joint strength by 0.92 kN, and a Zn–Ti Carbide coating raises TS from 118 MPa to 203 MPa and hardness from 139 HV to 211 HV. Pre-drilled holes improve material mixing and decrease IMC thickness. However, challenges persist regarding fatigue behavior and corrosion studies in Al–Mg welds, alongside insufficient research on thermal-mechanical interactions. While pilot hole techniques prove effective in linear FSW, they are underutilized in FSSW.

*Simulation and modeling approaches in FSSW*

Numerical modeling in Friction Stir Spot Welding (FSSW) is crucial for understanding heat generation, material flow, tool-workpiece interactions, and defects. Developing accurate simulations is complicated due to extensive plastic deformation and boundary conditions. Early simulations used conventional finite element analysis (FEA) with Lagrangian methods, which faced mesh distortion issues, especially during the plunge phase, reducing accuracy. Hannachi et al. [595] compared arbitrary Lagrangian–Eulerian (ALE) and coupled Eulerian–Lagrangian (CEL) methods in ABAQUS/Explicit, finding CEL superior for mesh stability and temperature predictions, while ALE failed due to distortion. Additionally, they created a 3D model [596] analyzing friction’s effects on heat and material movement, highlighting the importance of precise friction conditions. Advanced numerical methods like CEL are essential for accurate FSSW simulation, yet microstructural and IMC integration needs further enhancement.

Rahim et al. [53] reported that predictive models and real-time monitoring could enhance FSSW processes. Yet, issues like material preparation and tool wear, and a lack of tools for their accurate modeling, hinder broader adoption of this technique in automotive and aerospace industries.

Summarizing, FSSW efficiently joins Mg alloys using rotating tools, creating high-strength, low-defect connections. Key benefits include fine microstructures, minimized brittleness, and effective joint performance with ongoing research for process improvements.

#### 3.2.6. Controversies Related to FSW and FSSW of Mg Alloys and Discussion on the Distinct Applications of These Methods in Industry

Literature on FSW and FSSW of Mg alloys reveals contentious mechanisms regarding material flow, intermetallic compound (IMC) formation, and joint strength in similar and dissimilar joining [48,597,598]. Key debates include material transport beneath the tool, with some studies favoring atomic diffusion during dissimilar metal joining, while others emphasize severe plastic deformation [48,53,597]. Texture softening effects are debated, where grain refinement through dynamic recrystallization improves strength, yet localized crystallographic textures may weaken tensile properties [48,492,597,598,599]. The formation of brittle IMCs like Mg_17_Al_12_ during Al–Mg joining raises disputes over controlling their growth [381], with conflicting views on heat input thresholds [48,53,492,600] and interlayer strategies [48,53]. FSSW nugget formation controversies focus on hook formation and joint failure mechanisms [168,601], such as the optimal plunge depth for material consolidation [53] versus preventing thinning [53,602]. Corrosion behavior in Mg alloys remains unpredictable [603,604], with tensions between grain refinement improving resistance and potential galvanic corrosion sensitivity, complicating the understanding of pure Mg’s corrosion mechanisms [50,604,605].

The continuous FSW method and the FSSW technique are advanced solid-state joining techniques that mitigate the challenges of Mg fusion welding, specifically by preventing IMCs and hot-cracking. FSW generates continuous seams while FSSW offers a localized, rivet-free alternative to the RSW method.

FSW employs a rotating tool to create a homogeneous seam along a joint line. Its applications include joining similar Mg alloys for structural components in aerospace and automotive contexts, where dynamic recrystallization enhances tensile strength and fatigue life. Additionally, it facilitates bonding dissimilar materials, such as Mg to Al or steel, while controlling brittle IMC formation through precise tool management. Industries benefit significantly, as FSW allows lightweight multi-material designs essential for aerospace and automotive sectors, contributing to reduced carbon emissions and improved electric vehicle performance.

FSSW, by contrast, is a non-consumable technique that creates individual metallurgical spot bonds via a plunging-and-retracting mechanism. It is effectively used for fastening overlapping Mg sheets in automotive body structures and for assembling multi-material components, including Mg bonded to Al or steel. Successful implementation needs careful tool design and sequencing to respect the melting properties of the materials involved, ensuring effective solid-state bonding. In the automotive sector, FSSW replaces traditional RSW methods, cutting costs associated with self-piercing riveting and adhesives, streamlining assembly processes. In aerospace contexts, FSSW efficiently fastens components where continuous seams are not necessary, minimizing thermal distortion.

Both FSW and FSSW enhance microstructural integrity and corrosion resistance when optimized, each chosen based on structural demands. FSW delivers continuous load-bearing capability for high-stress applications, while FSSW focuses on rapid, targeted fastening, thus decreasing cycle time in manufacturing.

In summary, FSW and FSSW are pivotal for joining Mg alloys and other materials, driving innovation in lightweight design across automotive and aerospace industries while maintaining structural integrity and performance.

### 3.3. Hybrid Welding

Hybrid welding of Mg alloys, especially laser–arc, provides fast speeds, deep penetration, and high-quality joints with low porosity. Techniques like Laser–TIG and Laser–MIG enhance stability, fill undercuts, and achieve high TSs [50,108]. Laser–arc hybrid welding of AZ31B alloy showed improved weld formation, with optimized parameters resulting in no defects and achieving a TS of 252 MPa and elongation of 11.2% [371].

#### 3.3.1. Laser–MIG Welding

Laser–MIG hybrid welding of Mg alloys is a niche research area aimed at joining lightweight structural components for automotive and aerospace uses. Combining the laser’s deep-penetration keyhole with MIG’s filler-metal capabilities, this technique achieves welds that reduce undercut and porosity while enhancing penetration depths significantly beyond MIG alone. The main alloys studied include AZ31B and AZ31, along with some work on AZ91D, AM50, and Mg–Li systems.

Laser–MIG hybrid welding of Mg alloys, such as AZ31, notably enhances weld quality, yielding deeper, narrower, and faster welds—up to 1.2 times quicker—achieving joint strengths of 87.5–97.8% compared to single welding methods. This hybrid approach reduces porosity, minimizes HAZ, and effectively manages evaporation [108,398,606]. Key advantages of this hybrid technique include improved efficiency, higher TS, and reduced defects due to the synergistic interaction between the laser and MIG arc, leading to more regular welds [607,608]. Critical considerations for welding by this technique encompass alloy selection (e.g., AZ31, AM50, AZ91), appropriate shielding gas (Ar or He) to curb oxidation, and managing defect control with lower laser power to negate porosity while ensuring high strength, along with distinctive grain structures in the weld zones [50,360,609,610,611,612]. Wang et al. [108] examined laser–arc hybrid welding of AZ31B alloy, finding optimal parameters of 3.5 kW, 1.8 m/min, and 100 A. This method minimizes undercut defects, enhances arc stability, and produces welds with 190 MPa strength and 12% elongation. Ren et al. [613] studied the effects of beam oscillation amplitude on AZ80 alloy clad with Al–Mg via laser–arc hybrid welding, finding that optimal oscillation of 1 mm improves weld quality while reducing defects and maintaining properties. Lang et al. [614] explored laser–arc hybrid welding of Mg alloys and high-strength steel DP980, achieving joint strength of 295.7 N/mm at PL = 360 W. The Mg/Fe interface includes α-Mg and Fe-based solid solutions. Increased temperatures from the laser enhance interfacial properties, reduce Mg/Fe energy, and improve joint wetting angles. Cracks arise from solidification shrinkage, influenced by reaction layers and reinforcement structures.

According to [372], oscillating laser–arc hybrid welding on AZ31B alloy reduced pores and achieved 13.2% elongation. A reduction of pores was also observed during laser lap-welding of galvanized steel/Mg assisted by ultrasonic vibration [615]. Saifudin et al. [616] found that Al oxide increases hydrogen solubility and porosity in molten Al. Laser–MIG hybrid welding reduces porosity of Al–Mg alloys to under 3% and improves ductility. 

Chinese filings hold most Mg-specific active patents (B23K26/xxx) in alloy processing, with no active US or EP patents found for laser–MIG hybrid welding of Mg alloys; only general welding apparatus or Al-alloy patents exist. Patent [617] described adaptive oscillation trajectory and waveform matching for Mg alloys. Patent [618] addressed a dual-wire configuration that enhances TS over single-wire usage. Patent [619] describes an offset laser and arc for Mg/steel interface temperature control. Patent [620] addressed laser self-fusion for aerospace magnesium castings. Patent [621] detailed a laser scan for Mg–Li, addressing flaws with heat treatment.

Innovation themes and technical development related to laser–MIG welding encompass four key areas:

1. Process parameter Windows focus on independent variables such as laser power (2–5 kW), welding current (80–150 A), welding speed (1–2 m/min), and laser–arc distance (optimal ~3 mm). Key findings demonstrate that laser power influences penetration depth while arc current affects bead width [108].
2. Porosity Mechanisms identify two pore types—keyhole-collapse and pre-existing hydrogen [622]—where hybrid techniques [623] and beam oscillation [372] help reduce porosity.
3. Microstructure discussions reveal a dual-zone architecture with distinct grain sizes influenced primarily by laser power [371].
4. Dissimilar and multi-material joining highlights the adoption of laser–arc hybrid techniques for Mg/steel and Mg/Al joints to meet automotive manufacturing needs, with specific patents and publications supporting these innovations [619,624].
5. Advanced Variants including Oscillation, Dual-Wire, and Ultrasonic Coupling:
  - Oscillating Laser–Arc: Tianjin University papers [371,372] and patent [617] showed that beam wobble introduces CSL boundary engineering (Σ13b, Σ29 boundaries from tensile/compression twins), raising toughness.
  - Dual-wire: Patent [618] enabled dissimilar-alloy joining with non-metallic dopants in the wire to reduce cost and energy.
  - Ultrasonic-coupled Laser–MIG: Patent [625] coupled ultrasonic vibration into the shared melt pool via three routes (workpiece, arc, pulsed laser modulation)—originally for Al alloys but architecturally applicable to Mg.

Sinusoidal power modulation suppressed keyhole bulges in AZ31B alloy welding [626].

There are limited active patents outside China, particularly in the US and EP, for laser–MIG hybrid welding. The oscillating laser–MIG technique is the latest high-performance advancement, highlighted by emerging academic research [372] and a patent [617]. Dissimilar-metal joining (Mg/steel, Mg/Al) is rapidly expanding due to multi-material automotive designs. Process monitoring integrated with AI for real-time pore detection is developing [271], exemplified by one patent’s [617] intelligent trajectory matching. Additionally, die-cast Mg alloys (AM50, AZ91D) are notably underserved in Laser–MIG applications, signaling a research gap crucial for the automotive industry.

The Laser–MIG hybrid welding has high initial equipment costs ($50,000 to $200,000+), offset by increased speed (up to 80% faster than standard MIG) and lower post-weld straightening costs. Suitable applications include shipbuilding for thick panels, automotive manufacturing for strong frame joints, and pipeline welding for large-diameter pipes with joint gaps. Laser–MIG hybrid welding is optimal for automotive, aerospace, and railway components [627], minimizing HAZ to preserve the mechanical properties of wrought or cast Mg alloys. This technique can also be accompanied by plasma metal inert gas droplet deposition (plasma MIG DD) when joining Mg alloys with Al and the latter with Cu [628]. 

#### 3.3.2. Laser–TIG Welding

Laser–TIG hybrid welding for Mg alloys merges laser deep-penetration with TIG’s gap-bridging, arc-stabilizing capabilities. Although research in this area is less than for laser–MIG hybrid welding, interest is rising due to automotive and aerospace lightweighting needs, leveraging magnesium’s low density (~1.74 g/cm^3^). Challenges include high vapor pressure, low boiling point (~1090 °C), and defects like porosity, undercut, hot cracking, and coarse HAZ grains [50]. Hybrid welding using laser–TIG introduces a high-speed technique for Mg alloy welding [108].

In laser–arc hybrid welding of Mg alloys, the laser delivers primary penetration energy while the arc (TIG or MIG) enhances weld pool width and stabilizes the keyhole. This hybrid method offers several advantages over traditional laser welding:

- It suppresses undercut and porosity by preheating the surface, allowing gas bubbles to escape [371];
- It increases welding speed, achieving 1.5× faster rates with significant penetration depth [392];
- It enhances arc stability by utilizing the laser plasma channel to guide the TIG arc, minimizing wandering [108];
- It controls grain morphology, resulting in a bimodal microstructure—columnar grains at the fusion line and finer equiaxed grains at the weld center, influenced mainly by laser power [371].

Li et al. [629] studied AZ31B alloy/Q235 steel welded joints via laser–TIG butt fusion welding, highlighting that controlling Mg combustion loss and improving interfacial reactions are crucial for achieving TS near 165 MPa.

***Process parameter optimization for similar Mg/Mg welds***

The most extensively studied alloy is AZ31B. Tianjin University [371,372] and Hohai University [108] groups have established clear parameter windows (Table 10):

At optimized conditions (3.5 kW/100 A/1.5 m/min), porosity-free and undercut-free AZ31B welds reached 252 MPa TS and 11.2% elongation [371]. A separate study at 3.5 kW/100 A/1.8 m/min achieved 190 MPa and 12% elongation [108].

***Oscillating Beam Laser–Arc Hybrid Welding***

A significant recent advance is the introduction of beam oscillation into the hybrid process. For AZ31B:

- Pore formation is inhibited when oscillation frequency ≥ 75 Hz and radius ≤ 0.5 mm—oscillation agitates the melt pool, promoting bubble escape and breaking columnar grains at the fusion line [372].
- Optimal parameters (radius 0.25 mm, frequency 75 Hz) maximize coincidence-site-lattice (CSL) boundary fraction (mainly Σ13b and Σ29 from {10–12} tensile and {11–22} compression twins), raising elongation to 13.2%—a 12.8% improvement over non-oscillated hybrid welds [372].
- Standalone oscillating laser welding (without arc) on AZ31 showed that low frequency (~50 Hz) and small radius (~0.5 mm) improve bead appearance; excessive frequency (>75 Hz) or radius (>1.5 mm) causes undercut or weld collapse [373].

***Laser–TIG Hybrid Welding for Dissimilar Mg/Al Welds***

This is the most distinctive application of laser–TIG specifically (vs. laser–MIG) for Mg alloys. Dalian University of Technology (Liu Liming group) [630], being the leading contributor, reported that

- AZ31 Mg/6061 Al lap joints were welded by laser–TIG hybrid welding using Zn–xAl alloy filler metals (x = 0–35 wt.%). Al in the filler acts as a “reaction depressant,” inhibiting dissolution of the Mg BM and the Mg–Zn reaction.
- Zn–15Al filler gave the maximum fracture load of 1475.3 N/cm—28% higher than with pure Zn filler. Excessive Al (beyond ~15 wt.%) triggers brittle Mg_32_(Al, Zn)_49_ ternary compounds, degrading performance.
- The TIG arc component in this configuration provides precise thermal control over the reaction layer thickness at the Mg/Al interface—a key advantage over laser–MIG, where droplet transfer can disturb the interface chemistry.

***Porosity Mechanisms and Suppression***

Porosity in Mg alloy welds arises from two sources: hydrogen gas dissolved in the melt (metallurgical porosity) and keyhole collapse (process porosity). The hybrid approach addresses both [50]:

- Arc preheating reduces the cooling rate, allowing hydrogen bubbles to escape before solidification.
- Keyhole stability is improved because the arc widens the keyhole opening, reducing the aspect ratio and the probability of keyhole collapse.
- Beam oscillation further agitates the melt pool to enhance bubble escape [372].

Patents relevant to Laser–TIG welding fall into three areas:

1. Laser–TIG Narrow-Gap Hybrid Welding

Patent [631] described a narrow-gap laser–TIG arc hybrid welding apparatus with a rotating tungsten electrode for thick-section joints. Patent [632] detailed laser/arc hybrid welding for stainless steel, titanium, and alloys at ~20 m/min, extendable to Mg-family light alloys.

2. Laser + Variable-Polarity Arc Hybrid Welding for Light Alloys (Mg relevant)

Patents [633,634] addressed laser hybrid welding, reducing porosity in light-alloy train structures using defocusing. Patent [635] described a two-step hybrid laser arc welding process for Ni/Fe alloys, extendable to Mg.

3. Laser–Arc Hybrid Process (general)

Patent [636] detailed hybrid welding using primary wire and cold wire for enhanced deposition efficiency. Patent [637] addressed a laser-leading hybrid welding apparatus with a trailing arc reheat weld pool. Patent [638] described laser–arc welding, controlling droplet size to minimize spatter.

No active patents claim laser–TIG hybrid welding of Mg alloys with specific compositions. The Dalian University of Technology’s work on Zn–Al filler welding of Mg/Al joints is mainly documented in open literature, not patents.

Technology maturity for various welding methods includes Mg/Mg laser–arc hybrid welding (TRL 5–6), optimized for lab use but nearing pilot scale, primarily using laser–MIG. Laser–TIG for Mg/Al dissimilar joints (TRL 4–5) has been shown with Zn–Al fillers, facing challenges in IMC control. Oscillating beam laser–arc hybrid welding shows TRL 4 but lacks commercial equipment. Narrow-gap laser–TIG (TRL 3–4) is patented, with no published Mg-specific trials. Post-weld corrosion performance is under-studied, notably regarding galvanic corrosion risks [639].

Emerging directions in laser–TIG welding include

1. Combining beam oscillation with TIG arc to reduce columnar grains and porosity, with improved stability for thin sheets.
2. Designing Zn–Al and Zn–RE filler wires for Mg/Al laser–TIG joints using “reaction depressant” concepts to minimize intermetallic compounds (IMCs) [630].
3. Utilizing activated flux TIG with laser assistance to achieve high TS and elongation via grain refinement [177].
4. Implementing oscillating laser–GMAW for thick sections, demonstrating scalability for industrial applications [640].
5. Adopting CNN-based porosity detection in in situ monitoring, which is applicable for Mg hybrid welding to address quality risks associated with porosity [641].

Laser–TIG hybrid welding offers a moderate to high cost–benefit ratio, combining laser heat with TIG precision for superior weld control. It is ideal for aerospace and titanium components, and it excels in high-strength alloys. It produces oxidation-controlled welds for specialty pressure vessels in demanding environments, despite slower travel speeds than laser–MIG hybrid welding. It is excellent for heat-input control, reducing the risk of Mg evaporation and defects. It is best suited for applications requiring high aesthetic quality, exact dimensional tolerances, and minimal distortion, such as aerospace panel assemblies. Additionally, laser–MIG/laser–TIG hybrid welding is worthwhile when it is necessary to join lightweight automobile body panels (mixing Mg and Al). The intense, focused laser heat combined with the arc creates narrow, manageable intermetallic compound (IMC) layers, preventing joint embrittlement.

#### 3.3.3. GMAW–GTAW Hybrid Welding

Mg alloys face high porosity, oxide film disruption, and narrow welding windows in arc welding. GMAW offers high deposition but has arc instability. GTAW provides stability and oxide cleaning, but is slow. Hybrid methods merge GTAW’s advantages with GMAW’s efficiency, similarly shown in steel [642], noted for Mg alloys in a 2021 review [50].

GMAW–GTAW hybrid arc welding of magnesium alloys is an emerging technique, as noted in [50]. However, dedicated experimental studies on this method for Mg alloys are limited. The technique’s existence and recent use were confirmed, highlighting laser welding as the best fusion method and FSW as the leading solid-state route, with key defects being microporosity, coarse grains, and HAZ softening. According to [50], for AZ31B alloy processed in GMAW–GTAW, an optimal weld was achieved with 225 A total current, 140 A bypass current, 21 V voltage, 2.8 m/min speed, and 5 mm tungsten-workpiece distance. TIG–MIG hybrid joint surpassed standalone TIG and MIG in UTS and YS, and elongation. Despite a larger HAZ, acicular ferrite microstructure exhibited ductile fracture with fine dimples, establishing a benchmark for GMAW–GTAW hybrid welding [642]. Liu et al. [643] joined 304SS with AZ31B alloy using MIG–TIG double-sided arc welding–brazing (DSAWB) with AZ31 alloy wire. The brazing interface formed through mutual diffusion of Al, Fe, and Mg, yielding α-Fe(Mg, Al) solid solution and FeAl IMC. The maximum joint TS was 235 MPa (93.98% of Mg alloy). Strength depended on joint forming and interface strength. Low heat input improved interface strength, while excessive heat led to uneven formation and decreased TS due to brittle IMCs.

Existing patents for GTAW-GMAW hybrid systems focus on stainless steel, high-strength steel, and aluminum, not Mg alloys [644].

Authors of [644] presented an innovative welding method using synchronized GTAW and GMAW torches on a robotic arm, enhancing narrow-gap welding in thick stainless and high-strength steel by preheating sidewalls and preventing lack-of-fusion defects.

According to [645], arc-sensing control in dual-gun welding optimizes filler position, oscillation, and inter-gun spacing, enhancing sidewall fusion in Al alloys near Mg alloys for lightweight applications.

As noted by [646], MIG arc creates a primary weld pool, while TIG arc re-melts it, achieving high-quality surfaces with efficient deposition, reducing tungsten erosion, and enhancing stability for Al and Mg.

There is a research gap in dedicated experimental studies on GMAW–GTAW hybrid weld microstructure, porosity suppression, and mechanical properties of Mg alloys (AZ31, AZ61, AZ91, WE43). This gap suggests potential for patent opportunities and further investigation.

GMAW–GTAW offers a stable dual-arc environment, providing high-quality welding with deep penetration and efficient deposition. However, it requires complex hardware, increases heat input, incurs higher shielding gas costs, and is preferred over lasers for advanced arc control.

Generally, as an economical alternative to lasers, it leverages the control of GTAW with GMAW’s efficiency. Thus, it is ideal for dissimilar metal joining, like Ti to Al, and specialized fabrications. This method avoids costly optics, enhancing gap bridging, microstructure quality, and reducing spatter while remaining cost-effective. It is viable for commercial applications such as hybrid automotive frames and bicycle manufacturing, where laser integration costs are prohibitive.

#### 3.3.4. Laser–USW Hybrid Welding

Laser-ultrasonic (laser–USW) hybrid welding of Mg alloys integrates laser beam welding and ultrasonic vibration to combat magnesium’s volatility and porosity through acoustic streaming and cavitation. This technique is essential for lightweight structural applications [440,647,648]. Applicable alloys include wrought magnesium alloys like AZ series (AZ31) and ZK series (ZK60), along with cast alloys (AM and AS series). It also enables dissimilar welding between Mg and Al or galvanized steel, often needing additional plating to avert brittle intermetallic [376,434,441,442,647]. Meng et al. [649] developed an effective method using a Ti interlayer to minimize Al–Mg interface reactions and enhance layer uniformity through laser–arc hybrid welding with 90 Hz oscillation, forming an Al–Mg–Ti system that improves shear force and weld strength. Fei and Lang [454] elaborated a novel method for reducing porosity in laser welding of Mg/Al dissimilar metals involving ultrasonic waves and dual magnetic fields. High-speed camera observations of the molten pool revealed that alternating magnetic fields enhance fluidity and refine grain size, while stable magnetic fields lower cooling rates and aid bubble escape, effectively minimizing porosity. Laser–USW process parameters are critical for weld quality, and laser power typically ranges from 1.5 kW to 4.0 kW, and welding speeds vary from 1.0 m/min to 3.0 m/min. Ultrasonic frequency is usually set between 20 kHz and 40 kHz, with amplitude monitored to avoid defects. He or high-purity Ar is recommended as shielding gas to limit oxidation [370,440,442,650]. Laser–USW is predicted to be used in the production of target thin-to-medium sheets 1.5 mm to 5.0 mm [440,651] and can support high-volume manufacturing, especially in automotive and electronics [434]. Joint efficiency is good, confirmed by 85% to 98% recovery of BM UTS [360,399,647] and improved YS by 5% to 7% [650], alongside preserving ductility [647]. Limitations of its method include residual porosity risks [370], brittle intermetallic [47,55,376,652], and complex equipment needs [454]. Current technology readiness stands at TRL 4–6, focusing on prototype demonstrations in aerospace and transportation applications [379,454,647,653].

#### 3.3.5. Plasma–MIG

Plasma–MIG hybrid welding is an advanced joining technique for Mg alloys, merging a constricted plasma arc with a consumable MIG wire arc. Its application focuses on automotive uses, offering controlled deep penetration while minimizing heat input, which reduces magnesium evaporation and distortion [628,654,655]. 

Hui et al. [250] examined Mg welding characteristics, focusing on plasma arc welding technology advancements, challenges, and trends. Key research areas include interface formation in Mg alloys and composites, process-structure-performance relationships, and the need for advanced welding methods to address existing issues effectively.

The plasma–MIG system employs two power sources, including the plasma arc achieving deep penetration (keyhole effect), while the MIG arc fills the weld seam [656]. The synergy ensures preheating with the plasma arc, while the MIG arc enhances gap bridging. The setup lowers electrode wire consumption by 10–25% and overall heat input by up to 25%, safeguarding against heat degradation in Mg alloys [261,654,656,657].

The method is effective for both wrought (AZ31, AZ91) [611,658,659,660] and cast magnesium alloys (AM50, NZ30K) [256,661,662,663,664], suitable for specific applications like high-pressure die-castings or thermal resistance.

Tight control over process parameters is essential due to magnesium’s characteristics. Typical ranges include plasma arc currents of 40–60 A, gas flow for plasma and shielding at 0.2–0.3 L/min and 15–25 L/min, respectively, MIG arc currents of 80–120 A, and a WS of 0.5–1.0 m/min [250,665,666]. This technique is effective for plates from 1.5 mm to 10 mm. It excels in thicker plates (over 3 mm) by allowing deep single-pass welding with minimal root spatter [42,156,169,654,667,668,669,670,671].

The Plasma–MIG system is proper for medium- to high-volume production. This technique is suited for automated mass production of lightweight automotive components, like seat frames and panels [250,291]. However, this welding method of Mg alloys is currently a low-volume, specialized process primarily limited to aerospace, automotive prototype development, and academic research. Due to high production costs and welding defects, industries typically use alternative techniques like FSW or TIG/MIG welding for mass manufacturing. Plasma–MIG welding minimizes grain coarsening and preserves excellent structural integrity [50], achieving joint TSs of 85% to 98% of the BM’s strength [250,654]. Addressing porosity and spatter during the plasma–MIG process of Mg alloys is critical, similarly to the case of Al alloys [672]. Precise control of plasma gas flow and speed can limit porosity to trace levels (<0.5%) [271,395,455]. Pre-weld cleaning to remove oxides and optimizing droplet transfer modes helps mitigate defects [50,271,291,673,674]. The process faces challenges, including magnesium’s low boiling point, leading to vaporization and potential instability [675,676,677], distortion risk due to thermal expansion [271,673,678], and complexity in equipment operation [656]. While plasma–MIG is a mature commercial technology (TRL 8–9) for joining thick aluminum alloys and steels, its application to Mg alloys presents significant technical hurdles that prevent large-scale industrial adoption [250].

Summarizing, hybrid welding of Mg alloys, particularly laser–TIG and laser–MIG, combines deep penetration with low heat input, enhancing joint quality and minimizing defects like porosity and cracking. This technique yields strong joints, achieving over 90% of BM strength. The synergy of high-power-density lasers and arc welding’s gap-bridging capabilities addresses issues of laser sensitivity to gaps and arc welding’s high heat input. Fast cooling and stirring in the hybrid molten pool improve microstructure. Optimal parameters like 3.5 kW laser power and 100 A arc current boost strength. However, challenges include high equipment costs, complex setups, and the necessity for shielding gas. GMAW–GTAW of Mg alloys combines the high deposition rate of GMAW with the superior arc stability of GTAW. This approach overcomes common Mg welding issues like porosity, cracking, and surface oxides, delivering high-speed, high-quality, and deeper penetration welds, particularly effective for Mg alloys such as AZ31B.

### 3.4. Brazing

Brazing involves using a lower-melting-point filler metal, heated alongside the BM, allowing the filler to melt without exceeding the BM’s melting point. This technique is suitable for welding dissimilar metals while minimizing IMC formation [55]. Brazing Mg alloys faces significant challenges, primarily due to the MgO surface oxide layer that inhibits filler wetting, the limited processing temperature range (~650 °C for AZ alloys), and magnesium’s high reactivity with atmospheric elements. Since around 2010, the field has progressed, driven by a need for lightweight multi-material applications in industries like automotive and aerospace. The patent landscape is primarily influenced by Chinese research entities, with academic investigations focusing on ultrasonic-assisted fluxless methods and dissimilar-metal joints. Four main available strategies for oxide management include

- Flux-assisted brazing, a traditional approach, using chloride or fluoride-based fluxes to chemically remove MgO. However, this approach carries corrosion risks due to flux residues.
- Ultrasonic-assisted (fluxless) brazing, which employs acoustic cavitation to disrupt the oxide layer, has proven effective, achieving notable joint strengths and discovering a new quasicrystal phase Mg_32_(Al, Zn)_49_ in recent studies [679].
- Scraped/mechanical brazing, where a solid filler rod is mechanically pressed against the BM, is another viable fluxless technique [680].
- Transient Liquid Phase (TLP) brazing is applicable for dissimilar joints (Mg/Ti) and shows promise, although prolonged bonding times can weaken joint strength due to intermetallic formation [681].

During the brazing process, very important is the choice of the proper filler material is very important. Filler systems and their brazing temperatures vary: the Mg–Cd–Al system (396–402 °C) achieves 53–71 MPa shear strength, but strength decreases with higher Al content [680]. Mg–Zn–Al–Ni–Ag–Ti–Cu–Mn system operates at 425–475 °C, enhancing diffusion and wetting CN105618955A. Mg–Nd–Cu–Ni–Zn system (465–490 °C) yields 136–145 MPa TS, suitable for solidus >550 °C CN105618955A. Zn–12Cd system operates at 365–390 °C, useful for Al/Mg dissimilar junctures CN104439590B. Al/Zn/Cu/Zn multi-interlayer (~400 °C) provides 85.26 MPa shear strength [679], while Sn–Al alloys are low-temperature, ultrasonic-assisted CN115415627B. Ni/Cu sandwiches (~500–520 °C) achieve 57 MPa shear strength [681]. Mg–Cd fillers possess low melting points but raise toxicity concerns. Patent [682] involves ultrasonic-assisted brazing of Mg and W alloys using an Sn–Al filler, with fluxless, low-temperature, and no vacuum methods. Patent [683] details a two-stage brazing process for AA6061 and AZ31B alloys using Zn–12Cd foil in a vacuum atmosphere at 310–390 °C. Patent [684] presents an Mg-based filler for RE Mg alloy brazing.

Many papers discuss brazing of Mg alloys, including similar, Mg/Al, Mg/steel, and Mg/Ti joints.

*Mg alloy/Mg alloy*

Brazing of similar Mg alloys minimizes melting losses and prevents welding defects such as dents. The brazing filler metal’s melting point must be lower than that of the Mg alloy, with commonly used fillers including Al, Mg–Al, and Al–Zn [55]. Diffusion welding was explored by Yang et al. [685] with Al–Si–Cu as an interlayer. They noted that varying welding times affected the joint’s microstructure, with longer times leading to a continuous interface and changes in crystal structure. The BM remains unmelted; however, insufficient heat input can result in defects in the area surrounding the interface. Yang et al. [680] simplified the brazing process of AZ31 alloy using the phase diagram method to create Cd22.8Mg9.2Al and Cd26.3Mg15.3Al filler metals. AZ31 alloy was brazed under flame heating. The filler metal’s microstructure comprised a Cd–Mg matrix, granular Al_12_Mg_17_, and massive (Al). Increased Al content raised hardness from 183.6 HV to 252 HV, with melting points between 396.1 °C and 401.5 °C. Fracture morphology shifted with Al increase, impacting shear strength from 71 MPa to 53 MPa.

*Mg alloy/Al alloy*

Jadav et al. [384] summarized the literature for Al–Mg joints by diffusion bonding, brazing, and soldering processes, with IMCs generated, tensile/shear strength obtained, and findings. Brazing Mg alloys to Al ones involves challenges due to reactive oxide layers (MgO and Al_2_O_3_), low Mg melting point (~650 °C), and a narrow temperature range. Issues include the formation of brittle intermetallic compounds (IMCs) like Al_12_Mg_17_, Al_3_Mg_2_, and Mg_2_Al_3_ at the joint interface. The Anhui Science and Technology University team developed Cd–Mg–Al fillers (Cd22.8Mg9.2Al and Cd26.3Mg15.3Al) for AZ31 Mg alloy brazing using low melting points (396–401.5 °C). Key findings show that Al content impacts joint strength (71 MPa at 9.2 wt.% vs. 53 MPa at 15.3 wt.%) and ductility, highlighting effective oxide removal without corrosive fluxes [680]. Qingdao University explored ultrasonic-assisted brazing of MB8 Mg alloy to 2024 Al alloy with a multi-layer interlayer. Al foil addition transformed Al_3_Mg_2_ IMCs into stable AlMg_4_Zn_11_ IMCs. Maximum shear strength reached 85.26 MPa. Ultrasonic cavitation enabled oxide-free wetting, establishing a viable flux-free method for Mg–Al brazing [679]. Ming et al. identified Mg_17_Al_12_ as a crucial interfacial IMC in Mg–steel joints with Al filler, highlighting the importance of controlling laser offset for joint strength, applicable to Mg–Al brazing [686]. The review by Way, Willingham and Goodall discusses filler metal design in Al–Mg joining, emphasizing oxide disruption, wettability, and IMC control essential for Mg–Al brazing in automotive lightweighting [429]. Solvay has active patents for a fluoroaluminate-based flux designed for brazing Al–Mg alloys (≥0.5 wt.% Mg), highlighting its significance for Mg + Al brazing. The flux comprises ≥ 65 wt.% KAlF_4_ or CsAlF_4_, with additives like Li_3_AlF_6_ and achieves brazing in controlled atmospheres at temperatures of ≥560 °C (KAlF_4_) or down to 450 °C (CsAlF_4_). It targets heat exchangers and structural components made of 5xxx-series Al alloys [687]. Asahi Kasei patents a method for joining flame-retardant Mg alloy to Al, or other metals, using high-speed bonding techniques, achieving at least 70% of the weaker metal’s shear strength and creating a solid-phase bond [688]. UACJ/Furukawa-Sky patent fluxless brazing processes in argon atmospheres, utilizing Mg to reduce oxide layers [689]. Kobe Steel/DENSO’s patent outlines a fluxless filler of Al–Si–Bi–Mg for heat exchangers, enhancing oxide disruption and ensuring effective bonding in inert gas at 560–620 °C [690].

Core technical challenges include oxide removal, with MgO being more stable than Al_2_O_3_ and resistant to fluxes. Literature identifies three approaches: mechanical scraping/ultrasonic cavitation, reactive flux chemistry, and using Mg-in-filler as a reductant. Control of intermetallic compounds (IMCs) like Al_12_Mg_17_ and Al_3_Mg_2_ is vital [680], while Zn additions transform them to AlMg_4_Zn_11_, enhancing toughness [679]. There is a patent gap for filler metal compositions for Mg to Al brazing, presenting IP opportunities. Additionally, the overlapping temperature ranges of Mg and Al complicate processes, favoring ultrasonic-assisted techniques over traditional methods.

*Mg alloy/steel*

**Figure 6:**
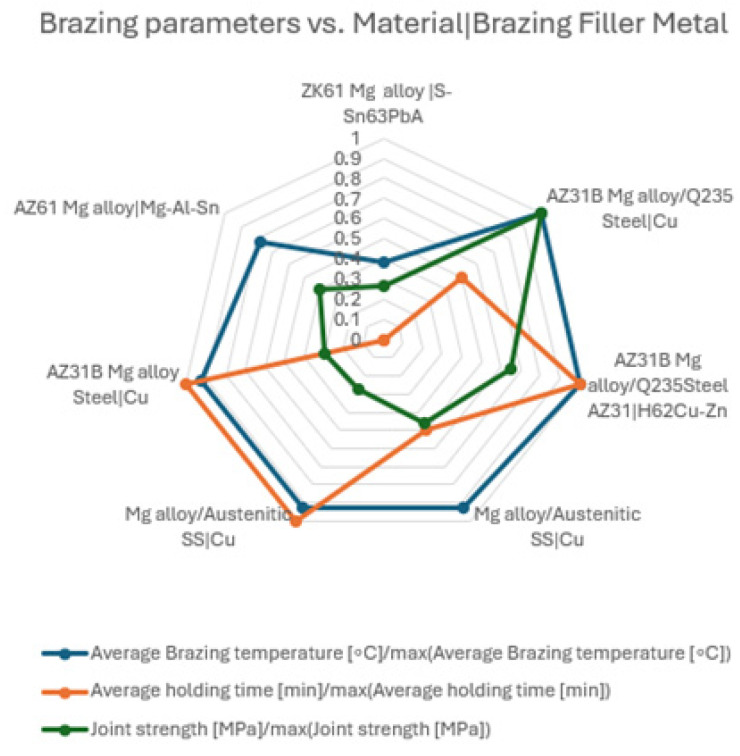
The brazing temperature and holding time during joining various Mg alloys with the same Mg alloys or various steels, accompanied by the use of different filler materials (based on data from [55]).

Mg alloys, specifically AZ31B and AZ91C, are commonly studied in connection with stainless steel (SS) due to automotive lightweighting demands. Key findings reveal various welding and brazing techniques that yield significant strength outcomes. For instance, Ming et al. [686] reported that laser welding–brazing of AZ31B alloy and AISI304 stainless steel allowed achieving 211 MPa joint strength, with intermetallic compounds Mg_2_Ni and Mg_17_Al_12_ affecting joint interface characteristics and fracture behavior. In diffusion-brazed AZ91C Mg with 316L SS, a two-stage process utilizing a copper interlayer resulted in shear strengths improving over 20% with increased temperature [691]. Interlayer-assisted laser welding of AZ31B with DP590 showed that a Sn interlayer outperformed Al, achieving a maximum load of 730 N [692]. Refill FSSW of AZ31 with galvanized DP600 produced high-strength, defect-free welds by forming Mg–Zn eutectic and IMCs. Zn coating acts as a brazing-like mediator enabling high-quality solid-state spot joining [693]. Ni-interlayer RSW techniques revealed better joint efficiency with austenitic SS interlayers than with Ni [694]. Contact reactive brazing is a method that utilizes metallurgical reactions, such as eutectic reactions, for joining materials. This technique offers advantages like varied configurations, low connection temperatures, minimal pressure, high strength, toughness, and compactness. The inclusion of RE elements in the brazing filler can enhance the microstructure in Mg alloy and steel brazing [55]. Jia et al. [695] found that adding rare earth La refined the interface grains, with optimal effects at 0.05 wt.%. Overall, enhancements from Al, graphene, and rare earth elements improve joint properties [40]. Rong et al. [696] applied laser welding–brazing with an alternating magnetic field to Mg/steel using a Ni interlayer. This method improved weld width, quality, and mechanical properties. Enhanced structures in the weld zones contributed to an optimal joint, boosting tensile and shear strength by approximately 15%. The brazing temperature and holding time during joining various Mg alloys with various steels, accompanied by the use of different filler material is shown in Figure 6 (based on data from [55]). The primary challenges in Mg/steel brazing are the immiscibility of Mg and Fe, oxide layers on Mg surfaces affecting wetting, and the differences in melting points and thermal expansion, necessitating careful design of the interlayer or processes to manage IMC thickness.

*Mg alloy/Ti alloy*

Laser, electron beam, and electric arc brazing of Mg alloys struggle with low melting points, causing melting issues. Diffusion welding avoids these problems, enabling effective brazing of Mg/Ti alloys. The joints’ performance and microstructure depend heavily on welding temperature and holding time [55]. Direct Mg/Ti brazing remains largely unexplored, with no specific studies on conventional filler metal brazing. Existing research [694] includes friction welding of AZ31B Mg to CP-Ti Grade 2, focusing on the Mg/Ti interface and intermetallic compounds (IMCs) from Al diffusion. Limited mutual solubility complicates conventional brazing, leading to a preference for friction welding or diffusion bonding.

*Mg alloy/Cu alloy*

Direct Mg/Cu brazing is underrepresented in the literature, primarily appearing as a part of Mg-to-steel TLP bonding studies. Ataei et al. [691], demonstrated diffusion-brazed AZ91C/316L SS with a Cu interlayer, forming Mg_2_Cu IMC and improved shear strength at higher temperatures. Yang et al. [680] introduced a low-melting Cd–Mg–Al filler for AZ31 that produces CdMg_3_ and Al_12_Mg_17_ phases, achieving shear strengths of 53–71 MPa. Brazing flux contained KCl, LiCl, CaCl_2_, CuCl_2_, and NH_4_HF_2_ for membrane breaking. Jiang et al. [697] demonstrated excellent weldability of Cu-plated AZ31 alloy using furnace brazing with Sn-based filler metal. Yang et al. [698] showed that the joint strength between AA5182Al and ZEK100Mg, using Ni as an interlayer with laser welding/brazing, depends on the Mg–Ni compound layer width. Wu and Liu [699,700] combined AZ31 with Ti–6Al–4V through laser brazing using Ni and Cu coatings. The optimal joint strength occurs with a 1.5 μm Ni coating, while a 19.7 μm Cu coating achieves 85.35% of Mg’s load capacity. These findings indicate that coatings can enhance BM properties and facilitate connections with other metals [680]. A significant challenge in Mg/Cu brazing is the formation of Mg_2_Cu and MgCu_2_ intermetallics and the risk of galvanic corrosion, with few dedicated studies in the existing literature on this topic [701,702,703].

In the area of brazing applied to various Mg alloys, some actual trends occur:

- The shifting towards ultrasonic-assisted brazing, which eliminates toxic fluxes, as seen in HIT Weihai’s Mg/W patent [684].
- Dissimilar joining of multi-materials such as Mg/Al, Mg/Ti, and Mg/steel is rising, driven by lightweight demands in EVs and aerospace. Laser welding–brazing is noted for Mg/steel joints, achieving high strength [686].
- Researchers focus on IMC engineering to convert brittle binary IMCs into tougher ternary phases, leveraging thermodynamic advantages [679].
- The development of RE-containing fillers for high-performance Mg alloys aligns with the rise in aerospace-grade magnesium alloys requiring matching fillers [684].
- A low density of active patents in the Mg alloy brazing domain suggests significant IP opportunities, particularly in fluxless ultrasonic Mg brazing, controlled-atmosphere processes for RE Mg alloys, and hybrid joints involving Mg/CFRP or Mg/ceramics. This indicates potential growth areas in technology, though it should be noted as an indication, not an exhaustive analysis of global patent activity.

Summarizing, Mg alloy brazing utilizes laser, electron beam, and arc as heat sources, alongside diffusion welding, enhancing brazing efficiency and simplifying the brazing process between Mg alloys and other metals.

### 3.5. Welding of Mg HEAs

Babenko et al. [704] examined the thermodynamic and physical parameters, synthesis methods, and phases of Mg (Mg)-containing high-entropy alloys (HEAs), noting that existing thermodynamic parameters fail to predict a single-phase solid solution accurately. The physical properties of these HEAs vary widely, featuring a complex multiphase structure with common elements like Al, Zn, and Cu. Phase formation prediction accuracy stands at 76%. The Mg addition typically reduces density, hardness, and strength, while increasing atomic mismatches and intermetallic phase likelihood; however, minor Mg additions may enhance properties. Synthesis methods were evaluated, highlighting mechanical alloying’s effectiveness for mixing immiscible elements, while melting methods expedite synthesis. The choice of synthesis technique and conditions has a considerable influence on the resulting phase structure of Mg-containing HEAs. Welding Mg High-Entropy Alloys (Mg-HEAs) is difficult yet achievable, with FSW commonly favored over fusion techniques to uphold strength and prevent issues like cracking and elemental separation. Fusion techniques like TIG/GTAW can be successful with optimized parameters, especially for non-ferrous Mg, requiring careful observation to manage unique microstructures and properties that vary from standard alloys [50,705,706,707]. Useful welding approaches for alloys include friction stir welding (FSW), which avoids melting, enhancing grain structure and hardness; TIG (GTAW), effective for Mg with precise AC/DC adjustments needed to manage oxide layers; and laser welding (LW), applicable to HEAs but requiring further research for Mg-HEAs. Each method addresses challenges in maintaining mechanical properties during the welding process [50,339,705,706,708,709,710]. Welding Mg alloys faces obstacles like microstructural complexity leading to brittle intermetallic, property deterioration from fusion welding, reducing strength and hardness, and tool wear causing fractures in HEA joints, requiring better tool material optimization [704,706,711,712,713]. Particularly, possess distinctive multi-element compositions that result in intricate microstructural changes during welding, which may result in the development of brittle intermetallic or segregation. Fusion welding frequently lowers strength and hardness in both the HAZ and FZ of Mg-HEAs. In FSW, wear debris (e.g., tungsten from WC-Co tools) can trigger fractures in HEA joints, necessitating the optimization of tool materials. Mg-HEA welding requires parameter optimization to reduce flaws and customize weld joints, with solid-state techniques like FSW and FSP enhancing microstructure [50,705,707,708,711]. Shangguan et al. [714] used a high-entropy alloy interlayer in resistance spot welding of Mg/steel dissimilar materials. The interlayer led to a distinct transition zone, free from defects. The interface featured various chemical bonds, including α-Mg and Fe_4_Al_13_. Joints with Al–Cr–Cu–Fe–Ni and Fe–Co–Ni–Cr–Mn interlayers showed improved mechanical properties by 24.4% and 51%, respectively, with a ductile-brittle mixed fracture mode. Additional research on optimizing parameters, examining novel methods such as Rotary Friction Welding (RFW), and creating Mg-HEA compositions tailored for welding is necessary [50,705,707,708,711].

Welding high-entropy alloys (HEAs) presents significant microstructural challenges primarily due to sluggish diffusion and lattice distortion characteristics. These issues often lead to defects such as elemental segregation and cracking. The complex elemental chemistries and rapid cooling rates during welding prompt non-equilibrium phase transformations, which compromise the stability assured by the high configurational entropy of these materials [715,716,717,718,719,720].

Welding of HEAs meets several key challenges:

1. Elemental Segregation: The non-equilibrium solidification in the fusion zone causes element segregation based on melting points and mixing enthalpies. This segregation results in interdendritic regions enriched with low-melting-point elements, such as copper or aluminum. Such variations lead to significant differences in microstructure and mechanical properties, affecting the overall performance of the welded material [721,722]. Mg-containing HEAs, including Al–Mg–Li–Zn–Ti and Mg–RE-based alloys, face segregation issues due to the large atomic size mismatch with transition metals, causing strong partitioning during solidification. Low melting point leads to concentration at boundaries, high vapor pressure limits homogenization temperatures, and low-melting intermetallic forms at grain boundaries, resisting dissolution [723,724].

Possible mitigating strategies include

- Rapid Solidification (water-cooled Cu mold; twin-roll casting (TRC); melt spinning) limits dendrite arm spacing; fine Mg_5_Gd dispersed uniformly at 27–200 °C/s vs. coarse segregates at 4.6–6.9 °C/s [725].
- Powder Metallurgy (PM) + SPS/HIP (mechanical alloying → spark plasma sintering or hot isostatic pressing) bypasses solidification entirely and exhibits near-net-shape capability [726].
- Multi-stage Homogenization Annealing (low-T pre-soak to dissolve fine precipitates → high-T soak for Mg diffusion) as the two-stage protocol showed to reduce residual eutectic to <1% in Mg-containing Al–Zn–Mg–Cu alloys [723,727].
- CALPHAD-guided Composition Design (high-throughput thermodynamic screening to select compositions with narrow solidification range (ΔT_sol–liq_ < 50 °C) and high Mg solid solubility) identifies single-phase BCC/FCC windows where Mg stays in solution [728,729].
- Equimolar or Near-equimolar Composition Tuning (avoiding Mg excess beyond the solid solubility limit and using VEC and ΔH_mix_ criteria to stay in single-phase field). DFT-screened LWHEAs (Al–Be–Mg–Ti-based) exhibited negative formation energy when Mg was kept within the solubility window [730].
- Thermo-mechanical Processing (TMP) (hot extrusion/rolling → dynamic recrystallization (DRX) → annealing). DRX redistributed solute while post-extrusion annealing dissolved residual boundary phases [731].
- Melt Stirring + Controlled Solidification Time (stir melt before casting; use high-gradient directional solidification or water-cooled molds; target solidification time ≤ 200 s) was validated for Mg–Y alloys, as stirring with rapid solidification yielded homogeneous Y distribution [732]. Such a strategy can also be beneficial for MRI 230 alloy [733].
- Micro-alloying with Grain Boundary Segregation Modifiers (adding Ce, Nd, or Zr at ≤1 wt.% to promote beneficial co-segregation and solute drag). Ce co-segregation with Zn at grain boundaries increases solute drag and refines grain size while improving ductility [734]

2. Phase Instability: High thermal inputs from fusion welding processes, such as GTAW or LBW, disrupt stable solid-solution phases, often producing brittle intermetallic phases (e.g., sigma phases). This phase instability compromises the toughness of joints, which are typically envisioned to retain simple FCC or BCC solid-solution structures [713,717,720,735,736,737]. Phase decomposition in HEAs is well-documented: nanocrystalline Co–Cr–Fe–Mn–Ni decomposes into Ni–Mn, Cr-rich, and Fe–Co nano-phases after only 5 min at 450 °C, driven by grain-boundary fast-diffusion pathways [738]. In eutectic Al–Co–Cr–Fe–Ni_2_._1_, short-time annealing at 800–1000 °C drives primary FCC dendrite fragmentation and needle-shaped B2 ordered phase formation through elemental partitioning [739]. For Mg-bearing systems, the additional risk is the formation of Mg_17_Al_12_ (γ), MgZn_2_ (Laves), and Mg_2_Cu-type intermetallics at weld interfaces and grain boundaries [740].

Possible mitigating strategies include

- Friction Stir Welding (FSW): This solid-state method uses a rotary tool without melting, preserving the base High Entropy Alloy (HEA) composition. Tool rotation is between 800 and 1400 rpm with a traverse speed of 25–100 mm/min, effective at maintaining Mg alloy integrity [741].
- Pulsed/High-speed Laser Welding: Utilizes a keyhole-mode laser with high power density (>10^6^ W/cm^2^) for low heat input, although it poses risks of porosity due to Mg vapor [50].
- Use of HEA Filler Metal: Employing an HEA filler wire helps suppress intermetallic compound formation through chemical matching and control of dilution [742].
- Post-Weld Heat Treatment (PWHT): This method stabilizes phases through solution annealing and aging, minimizing grain size and redistributing solutes [743].
- Vibration-Assisted FSW: Introduces mechanical vibrations to refine grain structure, enhancing nucleation rates [744].
- Composition Optimization: Utilizing CALPHAD thermodynamic screening to adjust elemental ratios is critical for reducing hot-cracking susceptibility [728].
- Grain Boundary Engineering: Adding nanoparticles to the weld pool can refine grains, increasing boundary density to reduce cracking risk [745].

3. Cracking Susceptibility: Solidification cracking arises when low-melting-point liquid films within interdendritic channels rupture due to solidification shrinkage stresses. Additionally, liquation cracking occurs in the HAZ, where remelting happens in the brittle phases. The high thermal expansion coefficients and sharp temperature gradients in HEA weldments introduce residual tensile stresses, increasing the risk of crack propagation, further exacerbated where elemental segregation induces eutectic liquid formation [721,736,746]. Mg-based HEAs (e.g., Mg–Al–Zn–Li–Sn, Mg–Al–Zn–Cu–Mn, density < 2.0 g/cm^3^) utilize Mg’s lightweight benefits alongside solid-solution strengthening. However, welding challenges arise from Mg’s weldability issues—high thermal expansion, low melting point, solidification range, and HCP texture sensitivity—exacerbated by HEA complexity. Success requires crack-free welds with above 80% BM TS, controlled HAZ microstructure, and no liquation films. Observed issues include solidification cracking from a wide temperature range [747], liquation cracking from low-melting phases [748], porosity from gas entrapment [50], coarse grain growth from thermal gradients, residual stress cracking from thermal mismatch [749], and IMC embrittlement from multi-element interactions. The brittleness temperature range dictates solidification cracking susceptibility, with multi-element Mg-HEAs generally showing broader BTRs than binary or ternary alloys [750].

Potential mitigation strategies include

- Solid-state welding: FSW effectively addresses Mg alloy issues by avoiding liquid phase interactions, thus preventing cracking. The dynamic recrystallization leads to finer grains, enhancing ductility and joint efficiency [741,751]. For cast alloys, differential-rotation refill FSSW improves lap shear strength by 50% through dynamic recrystallization [571]. Key parameters include TRS (400–1200 rpm) and WS (50–300 mm/min) [493].
- Grain Refinement in Fusion Welding: It is vital to reduce hot cracking in fusion welding [745]. Techniques like the addition of inoculants, magnetic field-assisted TIG welding [177], and hybrid strategies like laser beam oscillation [752] improve grain characteristics and reduce cracks.
- Welding Process Parameter Optimization for Fusion Welding: Adjusting parameters such as welding speed [753], heat input [749], and shielding gas [639] can enhance the fusion welding outcomes. Optimizing these factors maintains the weld pool’s integrity, avoiding cracking and ensuring a durable joint.
- Filler Metal/Interlayer Design: Utilizing composition-matched HEA fillers prevents brittle intermetallic compound formation [742]. Introducing interlayers, like Sn, improves joint load capacity [692], while using HEA as a joining intermediate can prevent liquid metal embrittlement in joints [754].
- Alloy Composition Design Against Cracking: Controlling the solidification range and avoiding low-melting elements at grain boundaries can mitigate cracking. Grain refiners like Zr may enhance mechanical stability.
- Post-Weld Heat Treatment: PWHT relieves residual stresses but must be carefully managed to avoid strain-age cracking during reheating [755,756].
- WAAM with in situ FSP: WAAM combined with FSP helps mitigate residual stress and cracks during the fabrication of Mg-HEA components, overcoming conventional welding limitations [757].

4. Hardening/Softening: The management of drastic hardness gradients within HEAs is crucial. Brittle BCC zones can exceed extreme hardness levels, necessitating mitigation of uneven thermal histories and residual stresses, typically achievable through targeted post-weld heat treatments, deformation processing, or controlled-atmosphere annealing [758,759,760,761]. The interplay of phase transitions, grain growth, and residual stress contributes to localized mechanical inconsistencies [762,763]. The BCC phase is harder and more brittle, while the FCC phase offers ductility. Rapid thermal cycles induce uneven phase transformations, causing volume and hardness variations [762,763]. High-temperature processing leads to localized grain coarsening, softening the material due to fewer grain boundaries [735,764,765]. Additionally, thermal gradients and phase changes generate intense internal stress fields, heightening the risk of brittle fracture in the hard BCC matrix [761,766,767].

Mitigation strategies can include

- Post-weld heat treatment (PWHT) is utilized to enhance strength and reduce brittleness by promoting coherent precipitates within the welded material [713,768,769].
- Advanced processing techniques, like Laser Powder Bed Fusion (LPBF), leverage compositional tuning to ensure a dual-phase matrix that provides improved mechanical behavior under strain [770,771,772,773,774].
- Severe plastic deformation methods refine grain structure, thereby potentially increasing YS and hardness uniformity [759,775].

HEAs need advancements to transition from labs to practical industrial applications effectively. Current research gaps related to welding of HEAs include

1. Filler Metal Design: A need for specialized HEA filler materials arises to modify weld chemistry, suppress intermetallic precipitation, and control segregation effectively [735,776,777].
2. Advanced Process Optimization: Research is ongoing to explore high-density energy sources and advanced control techniques (e.g., laser beam oscillation), aiming to improve grain structures while minimizing defects during the welding process [776,777].
3. Predictive Modeling: Integration of CALPHAD modeling with welding simulations is necessary to predict phase stability and cracking within HEA systems, necessitating the expansion of databases for accurate phase transformation forecasts [778,779].
4. Solid-state Joining: Solid-state welding processes, like friction stir welding (FSW) and Rotary Friction Welding (RFW), can be advantageous for HEAs as they mitigate the common defects associated with fusion welding [705,711,735,780,781].
5. Process Dissimilar Welding: Utilizing HEAs as transitional buttering layers remains a promising avenue for addressing welding challenges between incompatible materials [706,782,783,784]. Shangguan et al. [714] introduced a high-entropy alloy interlayer in resistance spot welding of Mg/steel, resulting in defect-free transition zones and enhanced mechanical properties by 24.4% and 51% with specific interlayers.

Regarding the current level of advancement in the use of welding for Mg-containing HEAs, it can be noted that such a process is still particularly challenging due to their complex microstructures. Babenko et al. [704] evaluated the thermodynamics, synthesis methods, and phase behavior of Mg-containing high-entropy alloys (HEAs), noting that current thermodynamic models inadequately predict single-phase solid solutions. The physical properties of these HEAs exhibit significant variation, often characterized by complex multiphase structures with elements like Al, Zn, and Cu. The precision of phase formation prediction is 76%. Mg addition generally lowers density, hardness, and strength, while increasing atomic mismatches and the probability of forming intermetallic phases; however, small Mg additions may enhance certain properties. Mechanical alloying was found to be effective for mixing immiscible elements, while melting methods offer faster synthesis. Welding Mg-HEAs poses challenges, with friction stir welding (FSW) preferred to prevent cracking and elemental separation. Fusion welding methods like TIG/GTAW are beneficial for non-ferrous Mg, requiring careful monitoring for unique microstructures compared to traditional alloys [50,705,706,707]. Effective techniques for alloys include friction stir welding (FSW), which enhances grain structure without melting; TIG welding, which necessitates specific AC/DC settings for Mg; and laser welding (LW), promising for HEAs but needing more investigation for Mg-HEAs [50,705,706,708,709,710]. Challenges in welding Mg alloys involve microstructural complexities that generate brittle intermetallic phases, reduced mechanical properties from fusion welding, and tool wear affecting HEA joints [705,706,711,712,713]. Due to multi-element compositions, welding can lead to intricate microstructural changes, causing brittleness or segregation, while fusion processes often decrease strength and hardness in Mg-HEAs. FSW can produce wear debris that contributes to fractures, indicating a need for improved tool material optimization [50,705,707,708,711]. Further studies on parameter optimization and novel methods like Rotary Friction Welding (RFW) are essential for developing Mg-HEA welding [50,705,707,708,711].

To summarize, the welding of Mg-HEAs presents difficulties due to potential cracking and elemental loss issues despite their desirable properties, such as low density and high strength. Effective welding strategies include utilizing methods like Laser Beam Welding for refined microstructures and ensuring proper heat management to improve weld integrity. However, issues like porosity and hot cracking persist [785] and require ongoing research focused on process optimization [706] and innovative techniques, including the use of precise heat control, filler metal optimization, and stringent pre-cleaning [735] to enhance weld quality and overall material properties.

## 4. Summary Observations, Recommendations, and Conclusions

The conducted review of various methods used for welding Mg alloys allowed us to formulate several conclusions:

The Mg alloys that are most frequently welded usually belong to the AZ series (Mg–Al–Zn), valued for their outstanding weldability, decent strength, and accessibility. AZ31B is the most commonly welded wrought alloy, whereas AZ61A and AZ91D are favored for casting and structural applications, which is in agreement with information from [50].

The primary welding methods for Mg alloys include GTAW, GMAW, LBW, and FSW, with TIG being the most popular for quality and FSW as the leading solid-state option.

TIG and MIG are effective for welding Mg alloys, serving different purposes based on material thickness and production speed. TIG is ideal for high-quality, precise work on thin materials, while MIG is suitable for faster work on thicker sections. Both require thorough cleaning and use 100% Ar as shielding gas, with He–Ar blends for deeper penetration. Common filler metals include AZ61A, AZ101A, AZ92A, and EZ33A, available as TIG rods or MIG wire spools. MIG may result in higher porosity. MIG offers high deposition rates ideal for mass production but comes with higher heat input, leading to grain coarsening and increased porosity. TIG is best for precision, esthetic repairs, and producing the cleanest welds despite being slower and requiring skilled operators.

GMAW is employed for AZ (Mg–Al–Zn) and ZK (Mg–Zn–Zr) alloy series, particularly suited for medium to thick plate sections in high-volume automated manufacturing [291,786,787]. Ideal for wrought and cast magnesium alloys with moderate weldability [291], GMAW works well with AZ31B, AZ61A, AZ91C/D, and ZK60A, with considerations regarding solidification cracking and heat control [50]. The recommended thickness range is 3.0 mm to 12 mm, accommodating greater thicknesses using multi-pass setups and proper joint preparation [788], and similarly to [789]. GMAW is preferred in transitioning from prototyping to mass production [291,790,791], benefiting from sustained continuous runs and robotic integration, while achieving high deposition rates through pulsed-spray transfer [291,792,793]. Key welding parameters [790,794] include a current range of 80 A to 200 A, arc voltage of 18 V to 28 V, travel speeds from 30 cm/min to 80 cm/min, and wire feed speeds of 10 m/min to 20 m/min. Pure A or Ar + He shielding gases are recommended, ensuring an effective gas flow rate of 15 L/min to 25 L/min. Wire diameter generally ranges from 0.8 mm to 1.6 mm, with 1.2 mm standard for structural applications [50,291,788,795,796], while meticulous pre-weld cleaning and proper fixturing are essential to avoid defects [291,673].

Gas Tungsten Arc Welding (GTAW/TIG) of magnesium alloys employs alternating current (AC) to eliminate surface oxide layers [176,291,797]. Key parameters include the following: Current Type: AC with high-frequency start is essential for cathodic cleaning; Current Range: 60 A–250 A [798,799,800]; Voltage: 12 V–22 V [50,668,798]; Shielding Gas: Ar or He at (7–12 L/min) [798,801,802,803,804]; Tungsten Electrode: Zirconated or Ceriated, sized 1.6 mm to 3.2 mm [798,802]; Filler Material: AZ61A or AZ92A, sized 1.6 mm to 3.2 mm [801,802]. GTAW excels in joining Mg thicknesses from 1.0 mm to 6.0 mm, similarly to Al alloys [805]. For under 2.0 mm, single sheared edges are acceptable; for over 2.0 mm, double-bevel or V-groove joints are preferred to reduce distortion [806]. Due to its slow travel speeds (0.2 m/min to 0.5 m/min), it is suitable for low-volume production and custom prototyping [110,291,797,805], while high-volume needs shift to processes like GMAW, laser–GTAW, or resistance spot welding [50,176].

CMT, similarly to MIG and TIG welding techniques, caters to different requirements for Mg alloys. CMT excels with thin-gauge alloys due to its low heat input, minimizing porosity and alloying element loss, while producing finer microstructures. Mg’s reactivity necessitates effective shielding gases, while all methods cause softening in the HAZ; CMT performs best in minimizing this. For repairs, TIG is preferred, and for new lightweight structures, CMT is optimal. Common filler rods include AZ61A or AZ92A to mitigate hot cracking.

Cold Metal Transfer (CMT) for Al and, to some extent, for Mg is predominantly utilized in prototyping, custom repairs, and dissimilar metal joining, especially between magnesium and aluminum or steel. Its low heat input minimizes vaporization and thermal deformation, making it preferable [232,807,808,809]. CMT is optimized for both wrought and cast magnesium alloys such as the AZ-series (AZ31, AZ61, AZ91), ZM-series (ZM6 for casting repairs), and Rare-Earth series (Mg–Gd–Y–Zr for aerospace applications) [209,659,674]. Key welding parameters include a current range of 85 A to 170 A, arc voltage of 11 V to 14 V, and wire feed speed from 3 m/min to 7 m/min, using 100% Ar or Ar-He mixtures for shielding [208,227,232,238,674]. While ideal for joining parts from 0.5 mm to 3 mm thick, modified CMT can handle up to 8 mm [232,810,811,812]. CMT is primarily a low-volume process suited for specialty applications like aerospace and automotive prototyping, with high-pressure die casting or friction stir welding being preferred for mass production [50,238,674,808,813].

Plasma arc welding and micro-plasma welding enhance precision in Mg alloys, addressing GTAW limitations by ensuring deeper penetration, reduced heat-affected zones, and suitability for varying thicknesses.

Plasma electrolytic oxidation (PEO) transforms Mg alloy surfaces into dense ceramic layers, enhancing corrosion and wear resistance through electrochemical processes. Plasma arc weld-bonding (PAWB) merges welding and adhesive bonding for strong joints. Combined, PEO post-treatment provides additional protection for welds, addressing corrosion issues in aerospace and automotive applications, ensuring robust, durable assemblies in challenging environments.

Resistance welding of Mg alloys faces challenges due to material characteristics, requiring CET for better properties. Techniques like electromagnetic stirring improve joint strength, while REW offers robust solutions for joining various materials.

RSW of Mg alloys demands precise control to avoid weld cracking, expulsion, and electrode wear. Due to magnesium’s high electrical and thermal conductivity, it requires a welding current 2.5 to 3 times that of steel, paired with short weld durations [346,357]. Common alloys include AZ31B (≈3% Al, 1% Zn), AZ61 (prone to liquation cracks), AZ91 (mainly cast but usable as filler), and some Al alloys. Dissimilar joint welding encompasses Mg–Al and Mg–steel, using interlayers like Ni or Cu [40,346,814,815,816,817]. Key welding parameters involve a current range of 15 kA to 25 kA, short weld times (50 ms to 200 ms), and electrode forces of 3 kN to 9 kN, with pre-treatment using 2.5% chromic acid recommended [346,357,818]. RSW is efficient for sheet metals of 1.0 mm to 3.0 mm thickness [346,819,820,821], primarily applied in low-to-medium-volume production within automotive and aerospace sectors [819,822].

Laser welding of Mg alloys offers high energy density, low heat input, and rapid welding speeds, making it ideal for automotive applications. Key advantages include precision, minimized heat-affected zones, and deep penetration. Commonly used Nd: YAG and fiber lasers provide a needed very high efficiency. Challenges like porosity and thermal expansion can be mitigated through surface preparation and optimized parameters, resulting in strong, refined weld microstructures with high UTS. LBWable alloys include wrought AZ31 and AZ61, and cast AZ91, AM60, and WE54 [45,50,823]. Standard parameters involve laser power from 1.0 kW to 3.0 kW and speeds of 1.5 m/min to 3.0 m/min, with He preferred as shielding gas [45,50,824]. Effective for thicknesses of 1.0 mm to 5.0 mm, deep penetration up to 10 mm requires specialized methods [823,824,825]. Laser welding scales from medium to mass production, ideal for automotive components and specialized aerospace applications [826].

EBW of Mg alloys achieves high-quality, narrow welds with minimal thermal damage, especially effective for Mg–Al alloys (AZ31, AZ61, AZ91) and rare-earth alloys (WE43). Advantages include reduced defects, high depth-to-width ratios, microstructural refinement, and retained joint strength. Key challenges are porosity and keyhole stability, with cochleoid scanning improving outcomes and managing defects effectively. EBW of Mg alloys enables minimized oxidation and distortion through deep penetration and rapid travel speeds [50,413]. Common alloys suitable for EBW include AZ series (AZ31, AZ61, AZ91), ZK60, and rare-earth alloys like WE43, with Mg–Al-based alloys welding more smoothly [827,828]. Welding parameters vary, with typical accelerating voltage of 30–175 kV, beam current of 50–1000 mA, and speeds of 10–25 mm/s, necessitating careful control due to lower thermal properties [50,194,413]. Effective weld thickness ranges from 1 mm to 15 mm, with full penetration possible in thicker plates [50]. EBW is primarily used for specialized, mission-critical components, rarely suitable for high-volume production due to equipment costs [40,50].

Diffusion bonding of Mg alloys allows them to join below their melting point but requires high pressure and a vacuum to prevent oxidation. Ni or Cu interlayers enhance joint strength. Key factors include temperature, holding time, and pressure, with Al interlayers improving interface microstructure and reducing difficulty [55]. Diffusion bonding of Mg alloys creates atomic-level bonds below melting points [829], and thus is ideal for joining Mg alloys to dissimilar metals like Al and steel, minimizing brittle intermetallic compounds [462,830]. Common alloys include AZ31, AZ61, AZ80, and ZM51, with interlayers such as Zn, Sn, Cu, or Ag aiding atomic diffusion [458,462,831]. Mg alloys are frequently bonded to Al alloys (e.g., 6061, 7075, 1060), Cu, and Stainless Steel to create lightweight bimetallic structures [462,832,833,834,835]. Key parameters include temperatures of 350 °C to 450 °C, pressures of 5 MPa to 15 MPa, and holding times of 30 min to 2 h, typically in vacuum or inert gas [458,462,829,836,837,838,839,840]. Mainly suited for thin foils (0.5 mm to 5.0 mm), diffusion bonding is a low-to-medium volume batch process, fitting high-value aerospace and automotive applications, but unsuitable for mass production [841,842,843].

USW of Mg alloys necessitates enhancements focusing on corrosion resistance, USW parameter calibration, texture evolution, residual stresses, chemical composition impacts, and materials flow. Limited research exists on US welded joints, particularly regarding dissimilar joints; microstructural modifications and established models need further exploration for better outcomes and understanding.

USW or its variant USSW is commonly applied to Mg alloys, including AZ31, AZ91, ZEK100, and Mg–Zn–Y, also for joints with steel and Al [50,435,441,844]. USW employs high-frequency vibrations and pressure instead of external heat, with typical parameters of 500 to 2000 J energy, 0.5 to 3.0 s welding time, and 0.2 to 0.6 MPa clamping pressure [447,448,844,845,846]. Welded sheets are usually 0.5 to 3.0 mm thick [650,847]. USW excels in low-to-medium production volumes, especially for dissimilar material joints [438,447,845].

EXW of Mg alloys efficiently bonds dissimilar materials like Mg and Al, avoiding melting. The high-speed collision creates a wavy interface, enhancing strength. Key aspects include minimizing brittle IMCs, using gelatin mediums and inert gas to reduce defects, followed by hot rolling to improve properties. Challenges include Mg’s low ductility. EXW of Magnesium alloys is used for bi-metallic clad plates in automotive and aerospace applications. Preferred alloys include AZ31B, AZ61 [827,848], and AZX611 [470,849], clad to A6005C aluminum or mild steels [383,470,476]. Key welding parameters include Collision Point Velocity, targeted below 2400 m/s; Flyer Plate Velocity optimal between 250 m/s and 400 m/s for effective ejection without melting; and Collision Angle ranging between 2° and 15° [850]. Stand-off distance should be 0.5 to 1.5 times the flyer thickness [482,485]. Low-velocity explosives, such as Ammonium Nitrate/Fuel Oil (AN–FO), are used to prevent overspeeding, with a detonation velocity of 1900 to 3100 m/s [477,851,852]. Thickness ranges for the flyer plate are 1.0–5.0 mm, and for the base plate, 3.0 mm to 20.0 mm [470]. EXW of Mg alloys is a niche, laboratory-scale process with no commercial production, mainly focused on steel-clad plates [40,476,853].

FSW is an effective solid-state technique for joining Mg alloys, eliminating fusion-related defects. It generates heat via a rotating tool, refining microstructure to equiaxed grains. Key challenges include addressing texture-induced softening and optimizing parameters like speed and rotation to prevent defects. Commonly used AZ-series alloys benefit from FSW, enhancing mechanical performance while managing IMCs in dissimilar joints. Advantages include reduced distortion, high efficiency, and strong, ductile joints ideal for transportation.

FSSW of Mg alloys produces solid-state welds via localized heat and deformation, minimizing IMCs. Key parameters include TRS and depth, affecting microstructure and mechanical properties, especially in dissimilar Mg–Al welding.

Both FSW and FSSW are solid-state joining techniques for Mg alloys, avoiding solidification defects. FSW is a continuous linear welding process using a rotating tool, whereas FSSW is a spot-welding technique forming localized weld spots quickly. FSSW is ideal for lap joints, commonly used as a replacement for RSW. Both FSW and FSSW create fine microstructures through dynamic recrystallization, enhancing mechanical performance. FSW offers high-quality joints, while FSSW provides speed and low distortion, though each has limitations regarding clamping and load-bearing capabilities. Recently, the corrosion resistance of various Mg alloys [531,532,534] has been more intensively studied.

Welding parameters widely vary for both FSW and FSSW processes applied for Mg alloys, similarly to the case of Al alloys. Optimal welding parameters include TRSs of 800–2000 rpm, with 1200–1500 rpm minimizing micro-structural issues [18,53,741,854]. WSs for linear FSW range from 25 to 100 mm/min [50,741,855,856], while AFs typically lie between 3 kN and 7 kN [741]. Plunge depths for FSSW are 0.1–0.2 mm, with dwell times of 2–8 s [53,741,857]. FSW suits thicknesses from 1.0 to 12 mm (up to 25 mm with control), and FSSW operates best on thin sheets (0.8–4.0 mm) [53,858]. Joint efficiency is high (75–98% UTS) [18,53,859,860,861,862,863], with TRL ratings of 8–9 in automotive and 6–7 in aerospace applications, similarly to the case of Al alloys [48,864,865]. FSW/FSSW is primarily used for customized, lightweight components [492,866]. FSW is significant in experimental automotive, aerospace, and rail industries for weight reduction [48,53,492,505,866,867,868], while FSSW addresses Mg alloy limitations in research and localized assemblies [53,869].

Hybrid welding of Mg alloys using laser–TIG and laser–MIG offers deep penetration and low heat input, resulting in strong joints with over 90% BM strength. The combination of lasers and arc welding addresses gap-related issues and reduces defects like porosity and cracking. While optimal parameters enhance strength, challenges include high costs and complex setups. GMAW–GTAW integrates high deposition rates and stable arcs, overcoming common Mg welding issues effectively. Methods like laser–USW and Plasma–MIG exhibit low levels of TRL and need further research.

Mg alloy brazing uses laser, electron beam, and arc as heat sources, alongside diffusion welding, enhancing brazing efficiency and simplifying the brazing process between Mg alloys and other alloys.

Some welding methods, e.g., GMAW, whose use for welding Mg alloys is limited, are gaining popularity in additive manufacturing [110].

Welding of Mg HEAs offers low density and high strength, but struggles with cracking and elemental loss. Techniques like LBW, EBW, and FSW help, though issues like porosity and hot cracking remain.

Future welding trends for Mg alloys focus on solid-state techniques like FSW for joint integrity. Key areas include high-power fiber laser welding, AI optimization, and advanced dissimilar metal joining for structural designs [50,104,381,866,870].

Additional research on comparable Mg alloy joints is crucial for diminishing weight in the aviation and automotive industries. Important focus areas encompass enhancing additive manufacturing, managing combustion hazards, regulating microstructure, boosting crashworthiness, advancing mass production methods, and guaranteeing regulatory compliance for aerospace uses.

Furthermore, different joints of Mg with metals such as steel, Al, Ti, Cu, and Li are essential in the automotive, aviation, and aerospace industries, especially for their structures made of multiple materials. Important research domains associated with them encompass managing brittle intermetallic compounds, defects, and thermal expansion discrepancies, tackling corrosion challenges, improving additive manufacturing techniques, and utilizing machine learning for predictive modeling to boost joint efficacy and reduce defects. Additional advancement of solid-state methods is also required.

Furthermore, optimizing welding parameters for Mg-HEAs is essential in the automotive and aerospace industries to improve fuel efficiency and lower emissions. Precise control methods must be developed to tackle issues such as hot cracking and porosity. Research efforts must be enhanced on essential methods like FSW, GTAW, LBW, and hybrid laser–GTAW applied to Mg-HEAs. Research on factors such as heat input and the composition of shielding gas is crucial, together with the application of AI and modeling to enhance welding efficiency.

## Figures and Tables

**Table 4:** Characteristics of wire-arc DED process with different heat sources [226].

Wire-Arc DED System		Feature	Stability	Thermal Efficiency	Deposit Rate
GMAW-based	GMAW	Consumable wire electrode spatter	Poor	0.75–0.86	3–4 kg/h
	CMT	Reciprocating consumable wire electrode	Good		2–3 kg/h
GTAW-based	GTAW	Non-consumable electrode	Good	0.60–0.80	1–2 kg/h
		Separate wire feed process			
PAW-based	PAW	Non-consumable electrode	Good	0.47–0.75	2–4 kg/h
		Separate wire feed process			

**Table 5:** Weldability of Mg with the other materials as regards EBW (based on data from [410]).

Chemical Element	Mg	Ag	Cd	Al	Ti	Fe	Zr	Mo	W	V	Nb	Ta	Re
**Weldability with Mg regarding EBW**	Fully possible			With a special approved beam management method	Can be possible					Hardly possible			

**Table 10:** Parameter windows for laser–TIG welding of AZ31B.

Parameter	Optimized Range	Effect
Laser power	3.0–3.5 kW	Governs penetration depth; primary driver of grain-size difference between arc/laser zones
Welding current	80–100 A (TIG/arc)	Controls bead width; limited effect on penetration
Welding speed	1.5–1.8 m/min	Higher speed → less heat input → finer grains but risk of undercut
Laser–arc distance	~3 mm (optimal synergy)	Too large → independent heat sources; too small → arc instability

## Data Availability

No new data were created or analyzed in this study. Data sharing is not applicable to this article.
